# FASLG Derived from Fibroblasts in Hydroxyapatite‐Rich Microenvironment Induces Urothelial Anoikis to Trigger Randall's Plaque Exposure

**DOI:** 10.1002/advs.202521605

**Published:** 2026-04-02

**Authors:** Minghui Liu, Maolan Wu, Meng Gao, Yongchao Li, Miao Yuan, Zhangcheng Liao, Zhi Liu, Yizhou Wang, Yu Cui, Jinbo Chen, Cheng He, Zhiyong Chen, Feng Zeng, Hequn Chen, Zewu Zhu

**Affiliations:** ^1^ Department of Urology Xiangya Hospital Central South University Changsha Hunan China; ^2^ National Clinical Research Center for Geriatric Disorders Xiangya Hospital Central South University Changsha Hunan China; ^3^ Institute of Physiology University of Zurich Zurich Switzerland; ^4^ Department of Urology Peking Union Medical College Hospital Chinese Academy of Medical Science and Peking Union Medical College Beijing China; ^5^ Department of Urology Hunan University of Medicine General Hospital Huaihua Hunan China; ^6^ Department of Internal Medicine Section Endocrinology Yale University School of Medicine New Haven Connecticut USA

**Keywords:** Anoikis, CaOx stones, FASLG, Randall's Plaques, THY1

## Abstract

Calcium Oxalate (CaOx) kidney stones with high recurrence are a major clinical and economic health burden. Randall's Plaques (RP) serve as a nidus for CaOx stones, but it remains poorly understood how renal interstitial hydroxyapatite (HAP) deposition erodes through the papillary urothelium to create sites for urinary CaOx crystal adherence. Here, it is observed loss of urothelium above interstitial HAP deposition, and revealed that Fas Ligand (FASLG) derived from renal interstitial fibroblasts (RIFs) in HAP‐rich microenvironment induced anoikis of urothelium to trigger RP exposure to urine. Mechanistically, HAP interacted with membrane protein THY1 of RIFs, which increased the affinity of THY1 to SFRP1 but suppressed its affinity to NDP, leading to activation of GSK3α/β–β‐catenin pathway and thus upregulating FASLG. Moreover, the upregulated FASLG is identified as the predictor for recurrence in patients with CaOx stones following lithotripsy. Furthermore, Benarthin, a small compound binding to THY1, is found to inhibit HAP‐induced FASLG and thus attenuate the anoikis of urothelium in RP mice. It is anticipated that investigations of urothelial anoikis caused by FASLG from HAP‐induced fibroblasts will offer novel insights into RP exposure, enabling preventive strategies for CaOx stone formation.

## Graphical abstract

Randall's plaques (RP) serve as the nidus for calcium oxalate (CaOx) kidney stones. The current study reveals that hydroxyapatite (HAP) crystals activate the THY1–GSK3α/β–β‐catenin axis in renal interstitial fibroblasts (hRIFs), inducing FASLG secretion. This paracrine signal drives urothelial (hRPSECs) anoikis, disrupting the epithelial barrier and exposing the underlying RP. This discovery paves a new way for preventing stone recurrence using the small molecule Benarthin.

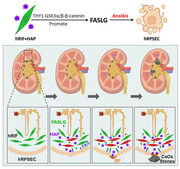

## 1 Introduction

Nephrolithiasis is a common and increasingly prevalent urological disease, affecting around 10% of the population globally [1, 2]. Calcium oxalate (CaOx) stones are the most common type, comprising about 80% of cases, with a recurrence rate of up to 50% within 5–10 years [3]. Complications can include severe pain, infections, renal impairment, and in severe cases, sepsis or death [4]. However, the pathophysiology of CaOx stone formation remains poorly understood, and effective prevention strategies are lacking [5], highlighting the need for further research.

Randall's Plaques (RP), first identified and named by Alexander Randall in 1937, were shown to play a critical role in the formation of CaOx stones. Endoscopic evaluations demonstrated that most CaOx stones were anchored to the renal papillary surface at sites of subepithelial calcium phosphate (CaP) deposition [6, 7, 8]; even spontaneously detached stones often retained adhesion points corresponding to these CaP‐rich areas [9]. Coronal micro‐computed tomography (micro‐CT) analyses of CaOx stones frequently revealed a ring‐shaped CaP structure at the apex [10], at least one internal CaP crystal deposit even in non‐attached stones [9], and a structural continuity between RP and the stone anchoring sites on the renal papillae [11, 12]. Fourier‐transform infrared microspectroscopy, X‐ray diffraction, and energy‐dispersive spectroscopy analyses indicated that the principal component of RP was hydroxyapatite (HAP), a crystalline form of CaP [8, 13, 14]. Furthermore, previous studies suggested that the presence of RP was often associated with an earlier onset of kidney stone formation and might indicate a higher risk of recurrence [15]. The percentage of papillary surface area covered by RP was found to correlate directly with the number of stones formed [16].

RP typically originated from the subepithelial deposition of HAP crystals beneath the basement membrane of the thin limbs of Henle's loop [8, 17]. These crystals grew within the collagen‐ and glycosaminoglycan‐rich renal interstitium [18], eventually coalescing and breaching the urothelium to become exposed to urine, thereby providing ideal adhesion sites for CaOx crystals and initiating CaOx stone formation [19]. Previous studies have mainly focused on the potential biological mechanisms underlying RP formation [20, 21, 22, 23, 24], such as oxidative stress, inflammatory responses, osteogenic‐like differentiation, and immune regulation. Calcium salt deposition in the renal interstitium was considered a normal aging process, and interstitial plaques had been commonly observed in both stone formers and non‐stone formers. However, not all plaques were associated with or led to kidney stone formation [25], indicating that the critical factor might be whether these calcium salts breached the urothelium and became exposed to urine. Only exposed HAP crystals can serve as adhesion sites for urinary CaOx crystals, thereby initiating stone formation. Nevertheless, it remains unclear how HAP crystals breach the urothelium and are exposed to urine.

In this work, we obtained rare renal papillary samples from CaOx stone formers who underwent nephrectomy for renal cell carcinoma. Von‐Kossa staining revealed the detachment and loss of epithelium in the mucosal layer surrounding sites of HAP crystal deposition. Using laser capture microdissection (LCM), we precisely isolated these regions and performed mass spectrometry (MS) and gene enrichment analyses, which identified Fas Ligand (FASLG), a gene associated with apoptosis, as a potential key molecule. Further experimental validation demonstrated that FASLG was primarily derived from human renal interstitial fibroblasts (hRIFs) treated with HAP crystals. Our findings revealed that HAP crystals acted on the membrane protein THY1 of hRIFs and activated the β‐catenin signaling pathway, leading to upregulation of FASLG expression. The hRIFs‐derived FASLG induced the anoikis of human renal papillary surface epithelial cells (hRPSECs), leading to the detachment and loss of urothelium. This disruption allowed renal interstitial RP to breach the urothelium and become exposed to urine, thereby providing anchoring sites for CaOx crystal adherence and facilitating stone formation.

## 2 Results

### 2.1 FASLG‐Mediated Anoikis of hRPSECs

**FIGURE 1:**
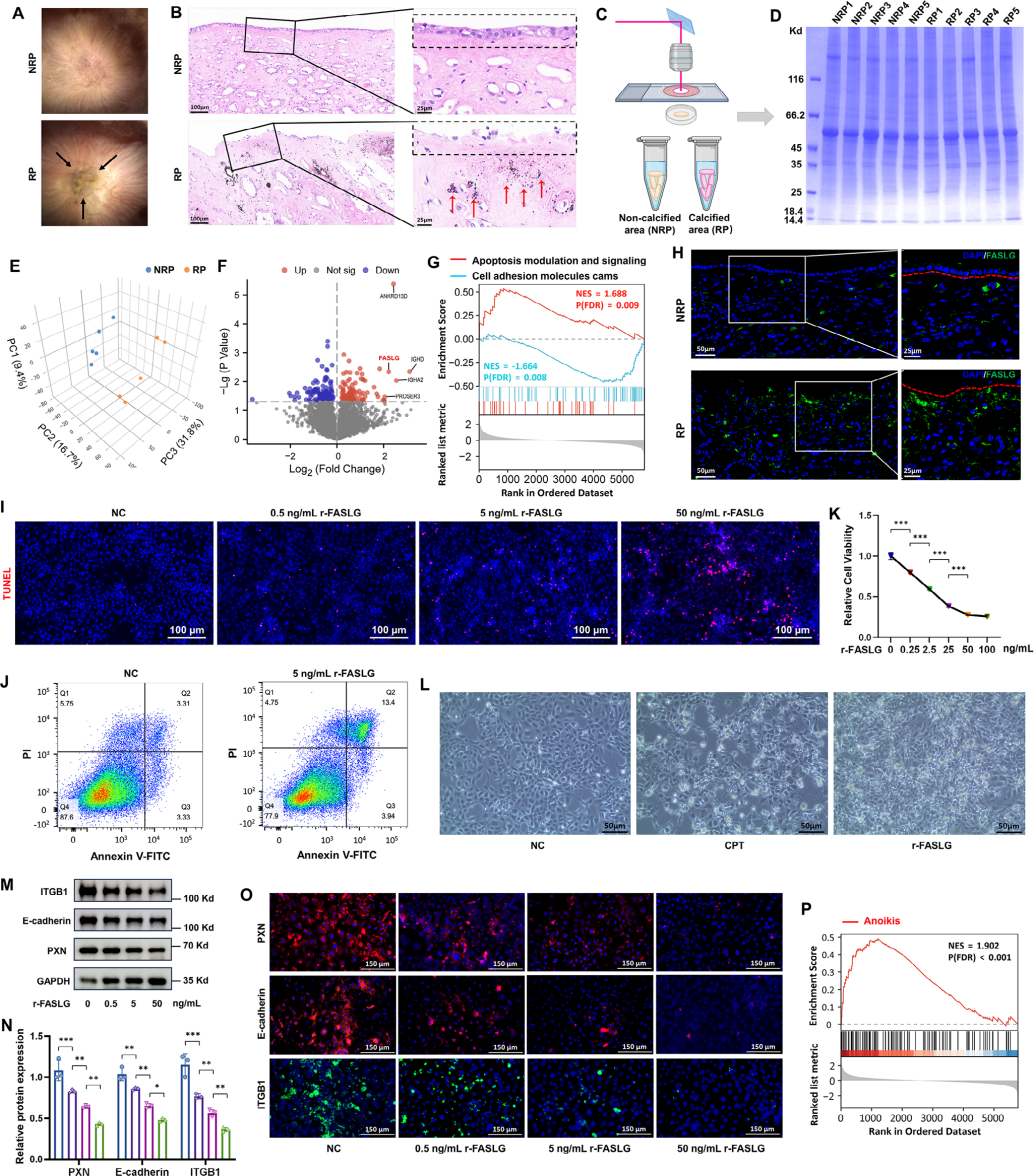
FASLG was upregulated in RP tissue and induced anoikis of hRPSECs. (A) Representative ureteroscopic images of normal renal papillae (NRP) and RP with CaOx stones (black arrows). (B) Representative Von‐Kossa staining of NRP and renal papillae with RP (red arrows) beneath human renal papillary transitional epithelial cells (hRPSECs). (C) Schematic representation of laser capture microdissection (LCM) applied to precisely isolate regions from paraffin‐embedded sections of NRP and RP renal papillae; *n* = 5 per group. (D) Proteins in samples isolated by LCM were resolved by 12% SDS‐PAGE and visualized by Coomassie Blue staining. (E) Principal component analysis (PCA) of proteins identified by mass spectrometry. (F) Volcano plot of mass spectrometry (MS) analysis of NRP and RP regions following LCM; *n* = 5 per group. Differently expressed proteins (DEPs) with fold change (FC) ranking top 5 were annotated. (G) Gene Set Enrichment Analysis (GSEA) of DEPs between micro‐dissected mucosal and submucosal regions of NRP and RP tissue. (H) Immunofluorescence (IF) staining for FASLG in NRP and RP tissues; *n* = 5 per group. (I) TUNEL staining showing apoptosis of hRPSECs treated with r‐FASLG at concentrations of 0, 0.5, 5, and 50 ng mL^−1^ for 3 days; *n*  =  3. (J) Flow cytometric analysis showing apoptosis of hRPSECs cultured for 3 days: in normal medium (NC), and in medium with 5 ng mL^−1^ recombinant FASLG protein (r‐FASLG); n  =  3. (K) CCK‐8 assay assessing cell viability of hRPSECs treated with r‐FASLG at concentrations of 0, 0.25, 2.5, 25, 50, and 100 ng mL^−1^ for 3 days; *n*  =  5. (L) Light microscopy showing the morphology of hRPSECs treated with CPT (20 umol L^−1^) or r‐FASLG (5 ng mL^−1^) for 3 days; *n*  =  3. (M‐O) hRPSECs were treated for 3 days with increasing concentrations of r‐FASLG: 0, 0.5, 5, and 50 ng mL^−1^, and adhesion molecules (PXN, E‐cadherin, and ITGB1) were determined by IF staining and Western Blot (WB); *n*  =  3. (P) GSEA of a custom anoikis‐related gene set integrating apoptosis, cell–cell adhesion, and focal adhesion genes between micro‐dissected mucosal and submucosal regions of RP renal papillae and NRP (*n* = 5 per group).

As visualized by flexible ureteroscopy, RP served as the nidus for CaOx kidney stones (Figure 1A; Figure S1A). We further obtained the renal papillae with RP from a nephrectomy specimen of a renal cancer patient (Figure S1B,C). Von‐Kossa staining showed the pathological feature of RP with renal interstitial HAP crystal deposition. Of note, the absence of the hRPSECs layer was frequently observed above the HAP crystal deposits in the renal interstitium of RP (Figure 1B). To further investigate this novel pathological phenomenon, we used LCM (Figure 1C,D) to precisely isolate the region of interest within RP (Figure S1D), with the non‐calcified area of NRP as the control group (Figure S1E), followed by MS analysis (Figure S2A–J). Principal component analysis of MS‐identified proteins showed the similarities within groups and differences between NRP and RP groups (Figure 1E), and we identified 229 differentially expressed proteins (DEPs) (RP = 5; NRP = 5) (Figure 1F; Table S1). Among these, FASLG, ranking fourth in the fold change (FC) of DEPs (FC = 4.7), caught our attention, since it has been reported to induce cell apoptosis [26, 27]. Corroboratively, Gene Set Enrichment Analysis (GSEA) (Figure 1G) revealed significant enrichment of apoptosis modulation and signaling (NES = 1.688, *P_FDR_* = 0.009) and cell adhesion molecules (NES = ‐1.664, *P_FDR_* = 0.008), suggesting enhanced apoptotic activity and reduced cell adhesion in RP tissue. Furthermore, immunofluorescence (IF) staining confirmed markedly upregulated FASLG beneath the sloughed hRPSEC layer in the RP tissue (Figure 1H; Figure S2K). To further clarify the effect of FASLG on hRPSECs, we co‐incubated recombinant human FASLG protein (r‐FASLG) with hRPSECs in vitro. We observed apoptosis (Figure 1I,J; Figure S2L) and decreased cell viability of hRPSECs in a r‐FASLG concentration‐dependent manner (Figure 1K). Notably, there was significant detachment of hRPSECs following r‐FASLG treatment, which was distinct from the cell shrinkage induced by the apoptosis inducer Camptothecin (CPT) (Figure 1L). Meanwhile, r‐FASLG suppressed adhesion molecules (PXN, E‐cadherin, and ITGB1) of hRPSECs in a concentration‐dependent manner (Figure 1M–O; Figure S2M), indicating that FASLG induces anoikis of hRPSECs. Moreover, GSEA showed that calcified region of RP was closely associated with anoikis (NES = 1.902, *P_FDR_* < 0.001) (Figure 1P; Table S2), supporting the role of FASLG in promoting anoikis in hRPSECs.

### 2.2 FASLG was Upregulated in hRIFs Upon HAP Crystal Stimulation

**FIGURE 2:**
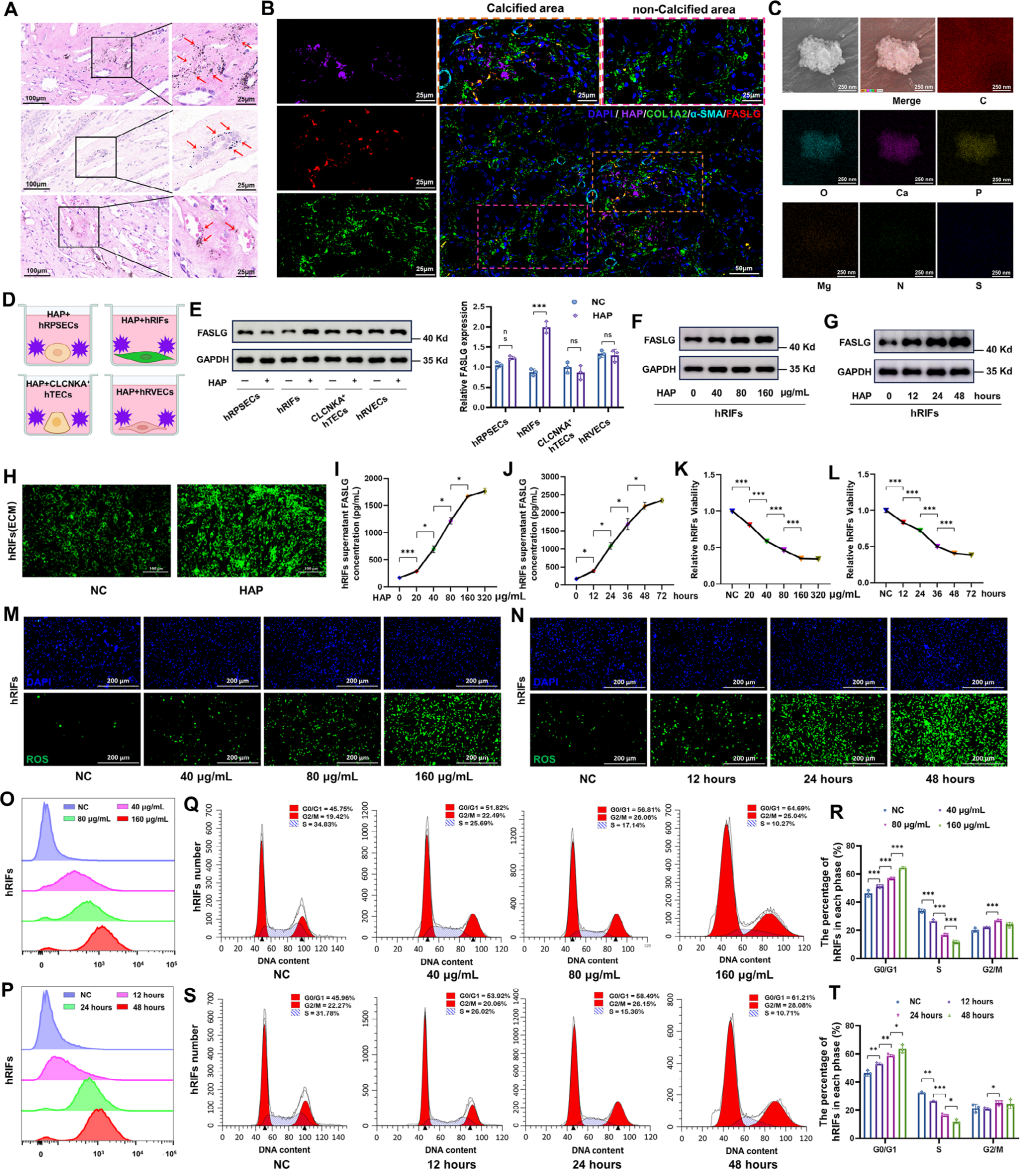
HAP crystals induced FASLG upregulation in hRIFs. (A) Von‐Kossa staining of RP, with calcium deposits around renal interstitium, renal tubules, and peritubular blood vessels. Red arrows indicated the location of calcium deposits. (B) Representative IF staining of a RP tissue section comparing a calcified area (orange dashed box) with an adjacent non‐calcified area (pink dashed box). Staining shows DAPI (blue), HAP crystals (OSTEO680, purple), COL1A2‐labeled human renal interstitial fibroblasts (hRIFs) (green), α‐SMA‐labeled vascular smooth muscle cells (VSMCs) (cyan), and FASLG (red); *n* = 5. (C) Scanning electron microscopy (SEM) showing the morphology of HAP, with energy dispersive X‐ray spectroscopy (EDS) determining the elemental composition of HAP. (D) Schematic illustration of the co‐culture system. (E) HAP crystals (80 µg mL^−1^, 500 nm) were co‐culture with hRPSECs, hRIFs, CLCNKA^+^ human renal tubular epithelial cells (hTECs), or human renal vascular endothelial cells (hRVECs) for 3 days, and FASLG was determined by WB; *n*  =  3. (F) FASLG expression in hRIFs was analyzed after 3‐day treatment with HAP crystals at concentrations of 0, 40, 80, and 160 µg mL^−1^; *n*  =  3. (G) hRIFs were treated with 80 µg mL^−1^ HAP crystals for 0, 12, 24, and 48 h, followed by analysis of FASLG expression; *n*  =  3. (H) IF staining detection of FASLG in the ECM of hRIFs following treatment with control medium or 80 µg mL^−1^ HAP crystals for 3 days; *n*  =  5. (I) hRIFs were treated with HAP crystals at concentrations of 0, 20, 40, 80, 160, and 320 µg mL^−1^ for 3 days, and FASLG levels in the culture supernatants were measured by enzyme‐linked immunosorbent assay (ELISA); *n*  =  3. (J) hRIFs were treated with 80 µg/mL HAP crystals for 0, 12, 24, 36, 48, and 72 h, and FASLG levels in the culture supernatants were measured by ELISA; *n*  =  3. (K) CCK‐8 assay assessing cell viability of hRIFs treated with HAP crystals at concentrations of 0, 20, 40, 80, 160, and 320 µg mL^−1^ for 3 days; *n*  =  5. (L) CCK‐8 assay assessing cell viability of hRIFs treated with 80 µg mL^−1^ HAP crystals for 0, 12, 24, 36, 48, and 72 h; *n*  =  5. (M, O) Intracellular reactive oxygen species (ROS) levels of hRIFs treated with HAP crystals at concentrations of 0, 40, 80, and 160 µg mL^−1^ for 3 days were evaluated through (M) qualitative fluorescence imaging and (O) quantitative flow cytometry; *n*  =  3. (N, P) Intracellular ROS levels of hRIFs treated with 80 µg mL^−1^ HAP crystals for 0, 12, 24, and 48 h were evaluated through (N) qualitative fluorescence imaging and (P) quantitative flow cytometry; *n*  =  3. (Q, R) Flow cytometry analysis of cell cycle distribution in hRIFs treated with HAP crystals at concentrations of 0, 40, 80, and 160 µg mL^−1^ for 3 days; *n*  =  3. (S, T) Flow cytometry analysis of cell cycle distribution in hRIFs treated with 80 µg mL^−1^ HAP crystals for 0, 12, 24, and 48 h; *n*  =  3.

As there was a loss of hRPSECs without direct contact with HAP crystals within RP tissues (Figure 1B), we speculated that HAP might induce other cells to upregulate FASLG of the extracellular matrix (ECM) and induce anoikis of hRPSECs. Our Von‐Kossa staining showed that HAP crystals deposited around renal interstitium, renal tubules, and peritubular blood vessels (Figure 2A). Meanwhile, the ascending thin limb of Henle loop, identified by chloride voltage‐gated channel Ka (CLCNKA), was reported to be the origin of partial HAP crystal depositions within RP tissues [28]. Therefore, IF co‐staining was utilized to determine whether FASLG were altered in these cells within RP tissues. FASLG was significantly upregulated in hRIFs surrounded with HAP deposits compared to the non‐calcified area within RP tissues, where RIFs were identified by the positive COL1A2 and negative α‐SMA to distinguish from vascular smooth muscle cells (Figure 2B) [23]. In contrast, there was no significant colocalization of upregulated FASLG and CLCNKA‐marked tubular epithelial cells (CLCNKA^+^hTECs) (Figure S3A) or CD34‐marked renal vascular endothelial cells (hRVECs) (Figure S3B) within RP tissues. To further explore whether HAP crystals regulate FASLG expression in the above cell types associated with their deposition sites, we isolated hRIFs, CLCNKA^+^hTECs, hRVECs, and hRPSECs from NRP tissues (Figure S3C–F). These cells were then induced with HAP crystals (Figure 2C,D; Figure S3H–K) with similar diameter ranges (the mode = 500 nm; Figure S3G) observed in RP tissues. Consistently, HAP crystals treatment significantly upregulated FASLG in hRIFs, but not in other cells (Figure 2E). Notably, HAP crystals induced FASLG of hRIFs in a dose‐ and time‐dependent manner (Figure 2F,G; Figure S3L,M). Crucially, this response was specific to HAP biochemical signaling, as SiO_2_ failed to trigger similar FASLG upregulation (Figure S3N,O). Furthermore, we observed a significant increase of FASLG within ECM of hRIFs following HAP treatment (Figure 2H, Figure S3R–T), which was further confirmed by the elevated FASLG in the supernatant of HAP‐treated hRIFs (Figure 2I,J). Conversely, ELISA analysis revealed that HAP stimulation failed to trigger FASLG secretion in hRPSECs (Figure S3U), thereby excluding the possibility of an autocrine regulatory loop in urothelial cells. Additionally, with increasing concentrations of HAP crystals and exposure duration, the viability of hRIFs progressively declined (Figure 2K,L). The intracellular ROS levels serve as a key indicator of the oxidative toxicity exerted by HAP crystals [29]. Qualitative fluorescence detection using the DCFH‐DA probe, coupled with quantitative flow cytometry analysis, demonstrated a marked increase in ROS levels with escalating concentrations of HAP crystals and prolonged exposure duration (Figure 2M–P; Figure S3P,Q). However, scavenging ROS with NAC did not attenuate FASLG upregulation (Figure S3V,W), indicating that FASLG induction is independent of oxidative stress. Moreover, flow cytometric cell cycle analysis revealed that HAP treatment led to an accumulation of hRIFs in the G0/G1 phase, accompanied by a decrease in the S‐phase cell population, in a dose‐ and time‐dependent manner (Figure 2Q–T). Notably, silencing FASLG failed to rescue HAP‐induced cell viability reduction and ROS generation (Figure S3X,Y), suggesting that FASLG does not mediate these cytotoxic effects in an autocrine manner.

### 2.3 HAP Crystals Upregulated FASLG of hRIFs to Induce hRPSEC Anoikis

**FIGURE 3:**
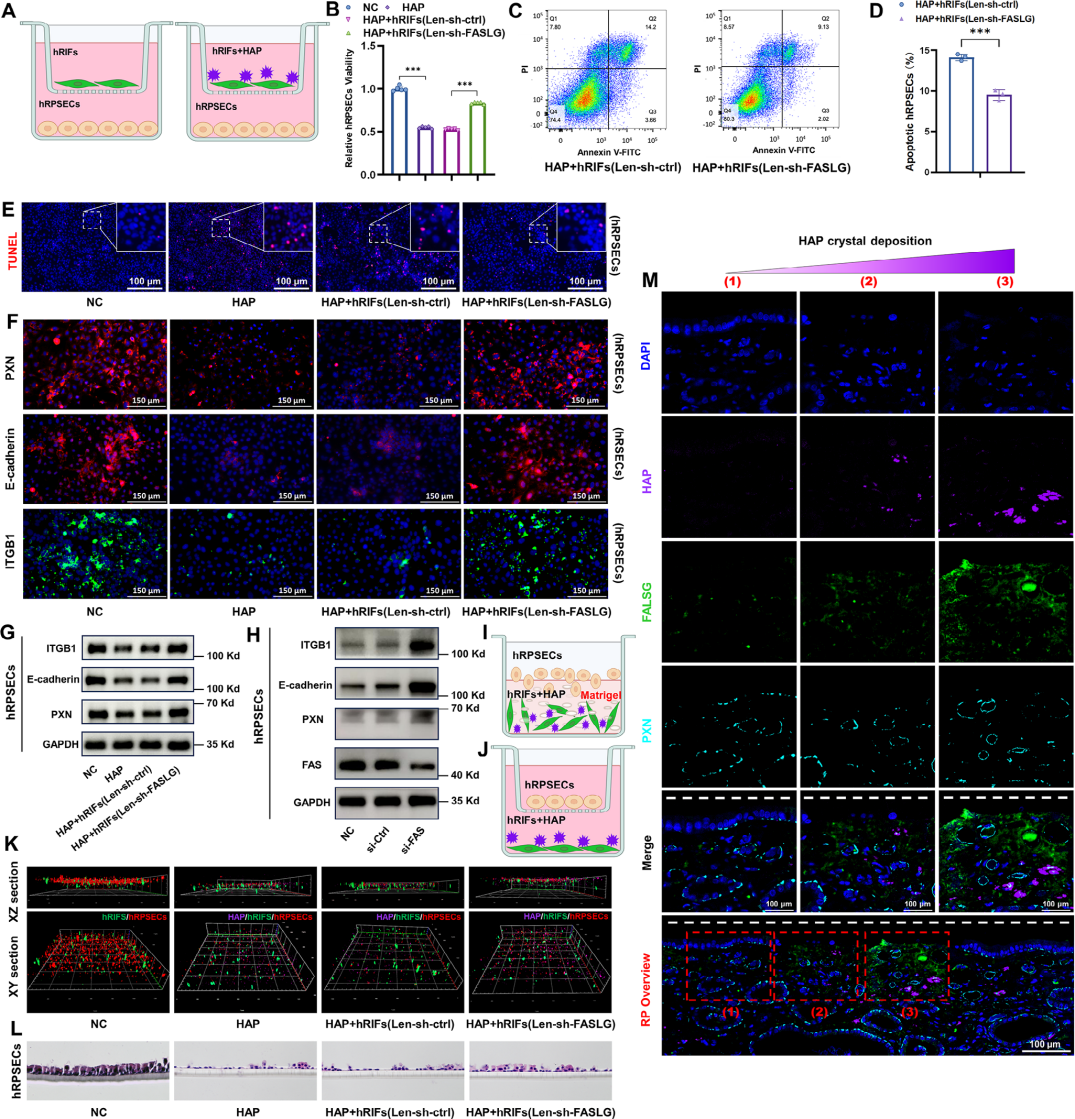
HAP crystals upregulated FASLG in hRIFs to trigger the anoikis of hRPSECs. (A) Schematic representation of the co‐culture model of hRIFs and hRPSECs. (B) CCK‐8 assay was performed to determine the viability of hRPSECs co‐cultured for 3 days with hRIFs, HAP‐treated hRIFs, HAP‐treated hRIFs transduced with lentiviral control shRNA (Len‐sh‐ctrl), or HAP‐treated hRIFs transduced with lentiviral shRNA targeting FASLG (Len‐sh‐FASLG); *n*  =  5. (C, D) Flow cytometric analysis showing apoptosis of hRPSECs co‐cultured for 3 days with HAP+hRIFs (Len‐sh‐ctrl), or HAP+hRIFs (Len‐sh‐FASLG); *n* = 3. (E) TUNEL staining showing apoptosis of hRPSECs co‐cultured for 3 days with hRIFs, HAP‐treated hRIFs, HAP+hRIFs (Len‐sh‐ctrl), or HAP+hRIFs (Len‐sh‐FASLG); *n* = 3. (F, G) hRPSECs were co‐cultured for 3 days with hRIFs, HAP‐treated hRIFs, HAP+hRIFs (Len‐sh‐ctrl), or HAP+hRIFs (Len‐sh‐FASLG), and adhesion molecules (PXN, E‐cadherin, and ITGB1) were determined by IF staining and WB; *n* = 3. (H) Adhesion molecules (PXN, E‐cadherin, and ITGB1) were assessed by WB in hRPSECs transfected with si‐FAS, co‐cultured for 3 days with HAP‐treated hRIFs; *n* = 3. (I) Schematic representation of the three‐dimensional (3D) co‐culture model of hRIFs and hRPSECs in Matrigel. (J) Schematic co‐culture model of hRIFs and hRPSECs with Transwell for HE staining of hRPSECs. (K) The 3D co‐culture of HAP‐treated (OSTEO680, purple) hRIFs (GFP, green) and hRPSECs (Cherry, red) in Matrigel was subjected to 3D confocal scanning after 3 days; *n* = 3. (L) H&E staining of paraffin embedded film with hRPSECs in the co‐culture system, with hRIFs in the lower chamber; *n* = 3. (M) Multi‐color IF staining of HAP (OSTEO680, purple), FASLG (green), and PXN (cyan) within RP tissues; *n* = 5.

To further investigate whether HAP‐treated hRIFs can induce anoikis of hRPSECs, we established a co‐culture system of HAP‐treated hRIFs and hRPSECs (Figure 3A). We found that HAP‐treated hRIFs significantly decreased the viability of hRPSECs (Figure 3B), and markedly increased apoptosis of hRPSECs (Figure 3C–E) with downregulated adhesion molecules (PXN, E‐cadherin, and ITGB1) (Figure 3F,G), which was attenuated by silencing FASLG of hRIFs (Figure 3B–G; Figure S4A–D) or FAS (FASLG receptor) of hRPSECs (Figure 3H; Figure S4E). In contrast, direct exposure of hRPSECs to HAP crystals failed to alter these adhesion molecules (Figure S4F,G), ruling out direct crystal toxicity. These results indicated that HAP crystals upregulated FASLG of hRIFs to induce anoikis of hRPSECs. Additionally, we established a 3D co‐culture system of hRIFs and hRPSECs by embedding HAP crystals and hRIFs in Matrigel, followed by seeding hRPSECs on Matrigel (Figure 3I), to mimic the microenvironment surrounding hRPSECs within RP tissue. There was suppressed adhesion of hRPSECs to the Matrigel with embedded HAP crystals and hRIFs, which was also attenuated by silencing FASLG of hRIFs (Figure 3K). Consistently, HE staining visualized significant hRPSEC detachment in the presence of HAP‐treated hRIFs (Figure 3J,L), a morphological finding quantitatively corroborated by a compromised epithelial barrier in transepithelial electrical resistance (TEER) assays (Figure S4H); notably, both morphological and functional defects were significantly attenuated by FASLG silencing. Interestingly, in a continuous field of view from the multi‐color IF staining of RP tissue, we observed a negative association of hRPSECs adhesion and HAP deposition within RP (Figure 3M). In areas with minimal renal interstitial HAP deposition (Figure 3M; left panel), hRPSECs remained attached, exhibiting low FASLG expression in the renal interstitium and high PXN expression in hRPSECs. In regions with moderate calcium deposition (Figure 3M; middle panel), partial detachment occurred, with elevated FASLG expression in the renal interstitium and nearly absent PXN in hRPSECs. In areas with extensive calcium deposition (Figure 3M; right panel), hRPSECs were absent, with high FASLG expression in the renal interstitium and undetectable PXN in hRPSECs. These findings confirm the crucial role of hRIFs‐derived FASLG in mediating the sloughing and loss of hRPSECs induced by renal interstitial HAP crystals.

### 2.4 Conditional Faslg Deletion in RIFs Attenuated RPSECs Anoikis in RP Mouse Models

**FIGURE 4:**
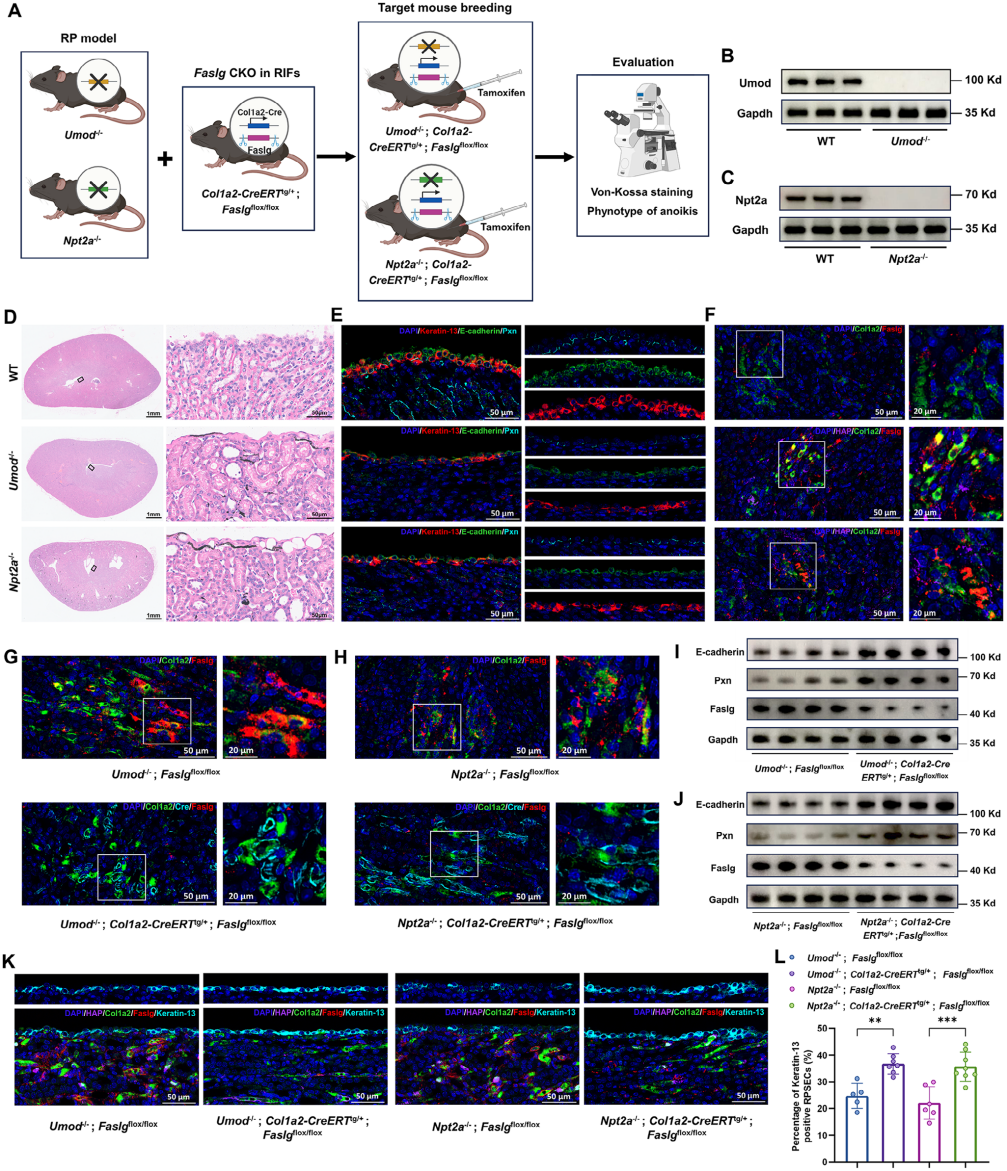
Conditional deletion of Faslg in RIFs attenuated hRPSECs’ anoikis in RP models of *Umod* ^−/−^ mice or *Npt2a* ^−/−^ mice. (A) Schematic diagram of breeding strategy for RP mouse models (*Umod* ^−/−^ or *Npt2a* ^−/−^) and mice with a conditional knockout (CKO) of Faslg specifically in RIFs (*Faslg^Col1a2^* CKO), mediated by inducible Col1a2‐CreERT recombinase. (B) WB analysis of Umod expression in wild‐type (WT) and *Umod* ^−/−^ mice; *n* = 3 per group. (C) WB analysis of Npt2a expression in wild‐type (WT) and *Npt2a* ^−/−^ mice; *n* = 3. (D) Von‐Kossa staining of renal papillary sections from WT, *Umod* ^−/−^, and *Npt2a* ^−/−^ mice; *n* = 6. (E) IF staining of renal papillary sections for Keratin‐13 (red), E‐cadherin (green), and Pxn (cyan) in WT, *Umod* ^−/−^, and *Npt2a* ^−/−^ mice; *n* = 6. (F) IF staining of HAP crystals distribution (OSTEO680, purple), Col1a2‐labeled RIFs (green), and Faslg (red) in renal papillary sections of WT, *Umod* ^−/−^, and *Npt2a* ^−/−^ mice; *n* = 6. (G, H) IF staining of renal papillary sections for Col1a2‐labeled RIFs (green), Faslg (red), and Cre (cyan) in *Faslg^Col1a2^* CKO mice with *Umod* ^−/−^ or *Npt2a* ^−/−^ background, and the corresponding controls without Cre; *n* = 5. (I, J) WB analysis of Faslg and adhesion molecules in renal papillary sections from *Faslg^Col1a2^* CKO mice with *Umod* ^−/−^ or *Npt2a* ^−/−^ background, and the corresponding controls without Cre; *n* = 4. (K‐L) IF staining of renal papillary sections for HAP crystals distribution (OSTEO680, purple), Col1a2‐labeled RIFs (green), Faslg (red), and Keratin‐13 (cyan), and quantification of Keratin‐13 positive RPSECs (%) in renal papillary sections of each group. For IF staining, there were 5 mice for *Umod* ^−/−^; *Faslg* ^flox/flox^ group; 7 mice for *Umod* ^−/−^; *Col1a2‐CreERT* ^tg/+^; *Faslg* ^flox/flox^ group; 6 mice for *Npt2a* ^−/−^group; 8 mice for *Npt2a* ^−/−^; *Col1a2‐CreERT* ^tg/+^; *Faslg* ^flox/flox^ group.

To investigate the pathogenic role of FASLG in renal interstitial calcium deposition‐associated anoikis, we generated two mouse models of RP (Figure 4A; Figure S5A,B) by knocking out *Umod* [30, 31] or *Npt2a* [32, 33], exhibiting renal interstitium HAP deposits akin to the pathogenesis of human RP formation. Western Blot (WB) analysis confirmed the absence of Umod in *Umod* ^−/−^ mice and Npt2a in *Npt2a* ^−/−^ mice (Figure 4B,C). Von‐Kossa staining revealed the interstitial CaP deposition in the renal papillae of both *Umod* ^−/−^ and *Npt2a* ^−/−^ mice (Figure 4D), indicating successful establishment of RP mouse models. *Umod* ^−/−^ and *Npt2a* ^−/−^ mice exhibited notable loss of RPSECs identified by Keratin‐13 and compromised epithelial adhesion characterized by down‐regulated Pxn and E‐cadherin in RPSECs (Figure 4E; Figure S5B,C), indicating an anoikis phenotype. To further assess whether Faslg derived from RIFs contributes to RPSECs detachment and anoikis, we generated RIFs‐specific conditional knockout (CKO) of Faslg mice (*Faslg^Col1a2^* CKO) with *Umod* ^−/−^ or *Npt2a* ^−/−^ background (*Umod* ^−/−^; *Col1a2‐CreERT* ^tg/+^; *Faslg* ^flox/flox^ and *Npt2a* ^−/−^; *Col1a2‐CreERT* ^tg/+^; *Faslg* ^flox/flox^) (Figure 4A; Figure S6A,B). IF staining demonstrated markedly increased Faslg expression in Col1a2‐marked RIFs of *Umod* ^−/−^ and *Npt2a* ^−/−^ mice (Figure 4F), which was significantly reduced in *Faslg^Col1a2^* CKO mice (Figure 4G,H; Figure S6C), and WB of mouse renal tissues showed the consistent results (Figure 4I,J). Meanwhile, *Faslg^Col1a2^* CKO mice showed restored expression of adhesion molecules (Figure 4I,J; Figure S6D,E) and improved retention of RPSECs (Figure 4K,L). Collectively, these findings demonstrated that Faslg expression in RIFs played a critical role in mediating RPSECs detachment and anoikis induced by interstitial HAP crystal accumulation in vivo.

### 2.5 HAP Crystals Upregulated FASLG of hRIFs via the THY1–GSK3α/β–β‐Catenin Axis

**FIGURE 5:**
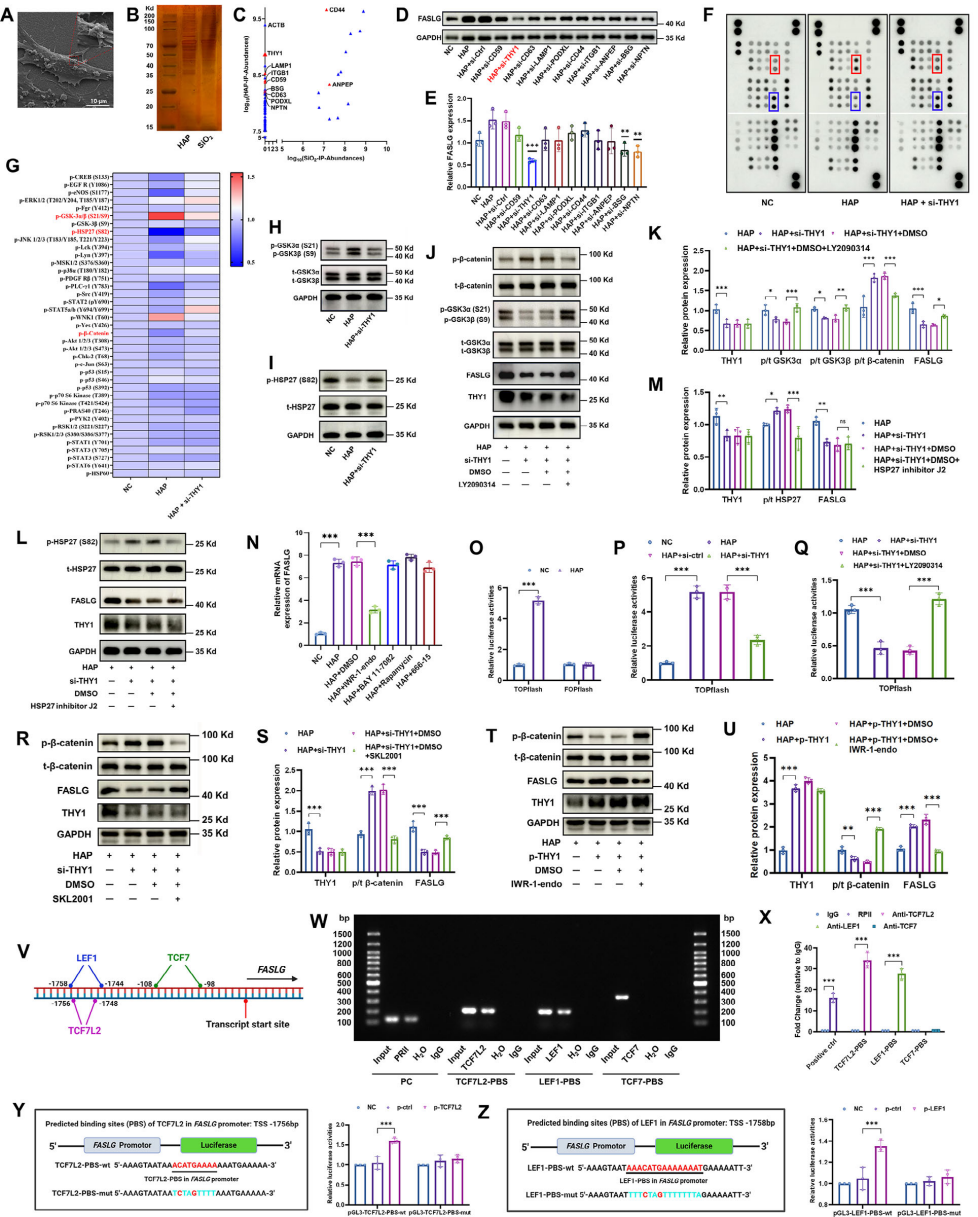
HAP crystals upregulated FASLG in hRIFs through activating THY1–GSK3α/β–β‐catenin pathway. (A) SEM observation of the hRIFs co‐cultured with HAP crystals; *n*  =  3. (B) hRIFs membrane proteins were co‐precipitated with HAP crystals, with the same diameter SiO_2_ crystals as a control, followed by SDS‐PAGE separation and silver staining. (C) MS analysis of proteins bound to HAP or SiO_2_ crystals, with a scatter plot showing protein abundance. All triangles in the plot represent proteins with an abundance greater than 10 million. The red triangles specifically denote the top 10 differentially expressed membrane proteins, which are labeled. Proteins plotted on the Y‐axis were below the detection limit in the SiO_2_ group. (D, E) A siRNA library was utilized to knockdown the top 10 membrane proteins of hRIFs binding to HAP crystals, followed by WB analysis of FASLG expression; *n*  =  3, **p < 0.01, ***p < 0.001 vs. the HAP+si‐Ctrl group. (F, G) Immunoblotting chip showing the phosphorylation pathway protein expression in normal hRIFs (NC), HAP+hRIFs (HAP), and HAP+THY1 knockdown hRIFs (HAP+si‐THY1) groups, and the phosphorylation level was illustrated. (H, I) WB validation of p/t‐GSK3α/β (S21/S9) and p/t‐HSP27 (S82) in NC, HAP, and HAP+si‐THY1 groups; *n*  =  3. (J‐M) WB analysis of p/t‐GSK3α/β, p/t‐β‐catenin, p/t‐HSP27 and FASLG in HAP‐treated hRIFs with si‐THY1 and either GSK3α/β inhibitor LY2090314 (20 nM) or HSP27 inhibitor J2 (10 µM); *n*  =  3. (N) The relative mRNA expression of FASLG was quantified by q‐PCR in hRIFs following treatment with HAP, or in combination with selective inhibitors of β‐catenin (IWR‐1‐endo; 5 µM), NF‐κB (BAY 11–7082; 5 µM), mTOR (Rapamycin; 50 µM), and CREB (666‐15; 0.5 µM); *n*  =  3. (O) TOPflash and FOPflash reporter assays in HAP‐treated hRIFs; *n*  =  3. (P) TOPflash reporter assay in HAP‐treated hRIFs with si‐ctrl or si‐THY1; *n*  =  3. (Q) TOPflash reporter assay in HAP‐treated hRIFs with si‐THY1 and LY2090314; *n*  =  3. (R, S) WB analysis of p/t‐β‐catenin and FASLG in HAP‐treated hRIFs with si‐THY1 and β‐catenin agonist SKL2001 (20 µM); *n*  =  3. (T, U) WB analysis of p/t‐β‐catenin and FASLG in HAP‐treated hRIFs with p‐THY1 and β‐catenin inhibitor IWR‐1‐endo; *n*  =  3. (V) Schematic diagram of the predicted FASLG promoter region bound by TCF7L2, LEF1, and TCF7. (W) Agarose gel electrophoresis of PCR products from input and chromatin immunoprecipitation (ChIP); *n*  =  3. PBS, predicted binding sites; PC, positive control. (X) ChIP combined with q‐PCR analysis of TCF7L2, LEF1, and TCF7 binding to the FASLG promoter; *n*  =  3. (Y) Schematic diagram of the pGL3‐basic luciferase reporter vector containing either the wild‐type (TCF7L2‐PBS‐wt) or mutant (TCF7L2‐PBS‐mut) sequence of TCF7L2 binding sites in the FASLG promoter region. hRIFs were co‐transfected with either p‐ctrl or p‐TCF7L2, and either pGL3‐TCF7L2‐PBS‐wt or pGL3‐TCF7L2‐PBS‐mut, and the relative luciferase activity (firefly/renilla) was determined. TSS, transcript start site; *n*  =  3. (Z) Schematic diagram of the pGL3‐basic luciferase reporter vector containing either the wild‐type (LEF1‐PBS‐wt) or mutant (LEF1‐PBS‐mut) sequence of LEF1 binding sites in the FASLG promoter region. hRIFs were co‐transfected with p‐ctrl or p‐LEF1, and either pGL3‐LEF1‐PBS‐wt or pGL3‐LEF1‐PBS‐mut, and the relative luciferase activity was determined; *n*  =  3.

We next investigated the molecular mechanism by which HAP crystals induce FASLG expression in hRIFs. The surface of HAP crystals is abundant in hydroxyl groups, phosphate groups, and calcium ions, enabling them to interact with various proteins through coordination bonding, electrostatic interactions, and hydrogen bonding [34]. Initially, we analyzed the interaction between HAP crystals and hRIFs using scanning electron microscopy (SEM). The results showed that HAP crystals adhered to the surface of the hRIFs membrane, and the hRIFs maintained a normal cell morphology (Figure 5A), suggesting that HAP crystals may interact with membrane proteins to influence the cellular phenotype. We then co‐incubated HAP crystals with membrane protein lysates from hRIFs to isolate HAP crystal‐bound proteins (Figure 5B; Figure S7A), followed by MS analysis (Figure S7B,C). SiO_2_ crystals of the same particle size were used as a control to exclude non‐specific adsorption from the crystal surface [35]. Compared to the SiO_2_ crystal group, 317 potential proteins were specifically bound to HAP crystals (Figure 5C; Tables S3 and S4). Subsequently, knockdown of the top 10 most abundant membrane proteins revealed that silencing THY1 significantly suppressed FASLG of hRIFs induced by HAP crystals (Figure 5D,E; Figure S7D–M). HAP co‐precipitation and WB validated the specific binding of THY1 to HAP crystals, with osteopontin (OPN) [36] and GAPDH as positive and negative controls, respectively (Figure S8A,B).

Given that THY1 is a GPI‐anchored protein lacking an intracellular domain, it transmits signals by associating with transmembrane coreceptors that recruit intracellular kinases and initiate phosphorylation cascades [37, 38]. To systematically explore these downstream events, we utilized a human phospho‐kinase antibody array to perform an unbiased screen of the signaling network (Figure S8C–F). The array results showed that p‐GSK3α/β (Ser21/9) and p‐HSP27 (S82) levels were markedly altered in hRIFs following HAP induction, and this effect was significantly attenuated by silencing THY1 (Figure 5F,G; Table S5), which was further verified by WB (Figure 5H,I; Figure S8G,H). To explore whether FASLG is downstream of GSK3α/β (Ser21/9) or p‐HSP27 (S82) regulated by HAP crystals interacting with THY1, HAP‐induced hRIFs with si‐THY1 were treated with the highly selective GSK3α/β inhibitor LY2090314 or HSP27 inhibitor J2. Intriguingly, GSK3α/β (Ser21/9), but not p‐HSP27 (S82), was the key mediator through which HAP crystals interacted with THY1 to upregulate FASLG (Figure 5J–M). To further delineate the regulatory mechanism, we also assessed the phosphorylation status of the activating sites of GSK3α/β (Tyr279/Tyr216). We observed no significant changes in these activating tyrosine residues among the NC, HAP+si‐ctrl, and HAP+si‐THY1 groups (Figure S8I), confirming that THY1 regulates GSK3 kinase activity primarily through the modulation of inhibitory serine phosphorylation rather than activating tyrosine sites. Moreover, pharmacological modulation of other array‐identified candidates, including WNK1 inhibition, eNOS activation, and Lyn inhibition, failed to affect FASLG expression, further supporting the specificity of the GSK3α/β axis (Figure S8J–L). GSK3α/β is known to regulate a broad spectrum of downstream substrates associated with cell survival and death, including β‐catenin, CREB, NF‐κB, mTOR, STAT3, STAT6, c‐Jun, p53, and p70 S6 Kinase [39, 40, 41]. The phospho‐kinase array profiling indicated that while p‐CREB and p‐β‐catenin exhibited alterations, other canonical targets such as p‐STAT3, p‐STAT6, p‐c‐Jun, p‐p53, and p‐p70 S6 Kinase showed no significant changes (Figure 5G; Table S5). Consequently, we focused our functional screening on the array‐identified candidates β‐catenin and CREB. To ensure comprehensive coverage, we also included the canonical GSK3α/β effectors mTOR [42, 43] and NF‐κB [44], which were absent from the array pane. Utilizing selective inhibitors, we found that β‐catenin served as the downstream of GSK3α/β (Ser21/9) to regulate FASLG mRNA (Figure 5N), but not NF‐κB, mTOR, and CREB (Figure S8M).

Moreover, the TOPFlash luciferase reporter assay was performed to assess the functional involvement of β‐catenin in this regulatory pathway. HAP treatment significantly increased TOPFlash activity in hRIFs (Figure 5O), whereas si‐THY1 treatment attenuated this activation (Figure 5P). Notably, the GSK3α/β inhibitor LY2090314 was able to restore and further enhance TOPFlash activity following si‐THY1 treatment (Figure 5Q). Moreover, HAP‐induced hRIFs with si‐THY1 reduced FASLG expression, while treatment with the β‐catenin agonist SKL2001 partially restored its levels (Figure 5R,S). Crucially, we found that specific inhibition of β‐catenin effectively reversed HAP‐induced FASLG upregulation, restoring expression to over 90% of the basal levels observed in controls (Figure S8N). Consistent with this, HAP‐induced hRIFs with p‐THY1 increased FASLG expression, which was partially suppressed by the β‐catenin inhibitor IWR‐1‐endo (Figure 5T,U). These findings suggested that β‐catenin served as a downstream factor of the HAP‐THY1‐GSK3α/β signaling pathway to regulate FASLG expression. To elucidate the mechanism governing β‐catenin stability downstream of the THY1‐GSKα/β axis, we analyzed its ubiquitination status. HAP treatment markedly decreased the levels of ubiquitinated β‐catenin compared to the NC group, indicating protein stabilization. Importantly, silencing THY1 reversed this effect, leading to a robust increase in ubiquitinated β‐catenin, whereas co‐treatment with the GSK3α/β inhibitor LY2090314 abolished this ubiquitination (Figure S8O). Furthermore, biochemical fractionation assays demonstrated that THY1 silencing significantly reduced the nuclear accumulation of β‐catenin (Figure S8P). Consistent with this, IF analysis visually confirmed that while the HAP‐treated group displayed high nuclear and low cytoplasmic β‐catenin intensity, THY1 silencing sequestered β‐catenin in the cytoplasm, preventing its nuclear translocation (Figure S8Q).

Given that β‐catenin lacks an intrinsic DNA‐binding domain and relies on the TCF/LEF family as its predominant nuclear partner to regulate downstream gene transcription [45, 46], and observing that FASLG mRNA was suppressed by inhibition of the β‐catenin pathway (Figure 5N), we employed the JASPAR database to predict the potential binding sites of β‐catenin‐TCF/LEF transcriptional complex in FASLG promoter. Setting a threshold of Score > 9.0 (Table S6), the candidate binding sites of TCF7L2, LEF1, and TCF7 were selected for experimental verification (Figure 5V). Chromatin immunoprecipitation (ChIP) combined with quantitative PCR (q‐PCR) confirmed that TCF7L2 and LEF1 directly bind the FASLG promoter, with an anti‐RNA polymerase II antibody serving as a positive control (Figure 5W,X). Notably, further analysis revealed that this physical occupancy is dynamically regulated: HAP stimulation significantly enriched the binding of both factors to the promoter, resulting in a >3‐fold increase for TCF7L2 and a >4‐fold increase for LEF1 compared to controls (Figure S8R). Furthermore, the dual‐luciferase reporter assay showed that TCF7L2 and LEF1 could interact with the predicted FASLG promoter region to activate its transcription (Figure 5Y,Z). Consistently, FASLG expression at both mRNA and protein levels was upregulated by HAP treatment in hRIFs, and these effects were significantly attenuated by silencing TCF7L2 or LEF1 (Figure S8S–V). To definitively prove that the phenotype requires the physical formation of the β‐catenin/TCF/LEF complex rather than just β‐catenin nuclear localization, we utilized iCRT3, a specific inhibitor that disrupts the β‐catenin‐TCF/LEF interaction. iCRT3 treatment almost completely abolished HAP‐induced FASLG upregulation, restoring expression to levels comparable to the negative control (Figure S8W). Collectively, these data confirm that HAP‐induced signaling recruits the β‐catenin/TCF7L2/LEF1 complex to the FASLG promoter to directly drive gene expression.

### 2.6 HAP Crystals Disturbed the Interaction of THY1 with SFRP1 and NDP to Activate GSK3α/β–β‐Catenin–FASLG Axis

**FIGURE 6:**
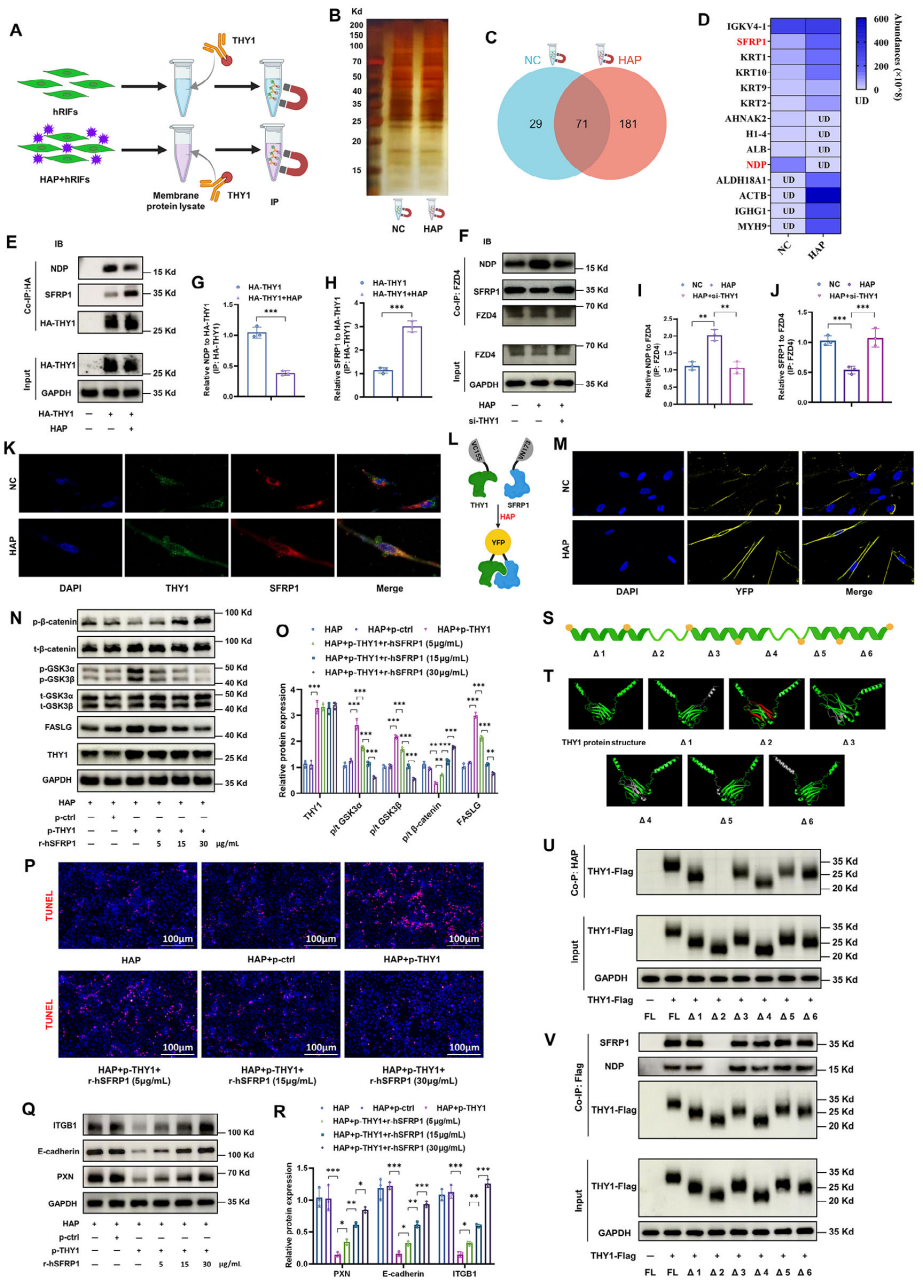
HAP crystals disturbed the interaction of THY1 with SFRP1 and NDP to activate GSK3α/β–β‐catenin–FASLG axis. (A) Schematic diagram showing membrane proteins from normal and HAP‐treated hRIFs were extracted and subjected to immunoprecipitation (IP) with a THY1 antibody. (B) SDS‐PAGE separation and silver staining analysis of IP products. (C) Venn diagram of IP products identified by MS. (D) Heatmap showing the top 10 proteins that bind to THY1 under normal and HAP‐treated conditions; UD, undetected. (E‐H) hRIFs were transfected with THY1 plasmid with an HA tag (HA‐THY1) and treated with HAP crystals, followed by IP‐WB to detect the interaction of SFRP1 and NDP with THY1; *n*  =  3. (F–J) hRIFs with si‐THY1 were treated with HAP crystals, and SFRP1 and NDP were determined in the anti‐FZD4 IP products; *n*  =  3. (K) Confocal microscopy analysis of THY1 (green) and SFRP1 (red) colocalization in hRIFs with or without HAP treatment; *n*  =  3. (L, M) Schematic diagram of Bimolecular Fluorescence Complementation (BiFC) assay for detecting THY1–SFRP1 interaction, and Yellow Fluorescent Protein (YFP) signal was captured by a confocal microscopy; *n*  =  3. (N, O) hRIFs transfected with p‐ctrl or p‐THY1 plasmids were treated with HAP crystals and recombinant SFRP1 protein (r‐hSFRP1) at 5, 15, and 30 ng mL^−1^ for 3 days, and protein expressions were determined by WB; *n*  =  3. (P) hRIFs transfected with p‐ctrl or p‐THY1 plasmids were treated with HAP crystals and r‐hSFRP1, then co‐cultured with hRPSECs for 3 days, and TUNEL staining showing apoptosis of hRPSECs; *n*  =  3. (Q, R) The adhesion molecules (PXN, E‐cadherin, and ITGB1) of hRPSECs were determined by WB; *n*  =  3. (S) Schematic diagram of the full‐length THY1 protein amino acid sequence, with a series of Delta1‐6 segment designed to be deleted in constructed THY1 plasmids. (T) Full‐length 3D structure of the THY1 protein with Delta 1–6 segments highlighted in silver or red. (U) hRIFs were transfected with plasmids carrying FLAG‐tagged full‐length (FL) or truncates, followed by WB to detect THY1‐Flag in HAP crystal precipitation, and (V) Co‐IP to determine interactions of THY1, SFRP1, and NDP.

Given that THY1 is a membrane protein and HAP binds to THY1, we further explored whether the interaction of HAP crystals and THY1 modulates the downstream GSK3α/β–β‐catenin pathway via altering the affinity of THY1‐binding proteins. Subsequently, we extracted membrane proteins from hRIFs treated with or without HAP crystals, followed by immunoprecipitation (IP) to identify those potential proteins binding to THY1 (Figure 6A,B). IP coupled with MS revealed that HAP crystals significantly altered the profile of proteins binding to THY1, as illustrated by that 25.3% of proteins were detected in both groups (Figure 6C; Figure S9A,B; Tables S7 and S8). Among the top 10 abundant proteins in each group, NDP and SFRP1 caught our attention, since NDP is a non‐canonical Wnt ligand binding Frizzled class receptor (FZD) to activate β‐catenin signaling pathway, whereas SFRP1 contains a cysteine‐rich domain similar to the Wnt‐binding site of FZD to inhibit it [45, 47].

Intriguingly, MS initially showed that NDP was largely reduced, while SFRP1 was dramatically increased in THY1‐immunoprecipitation (THY1‐IP) of HAP‐treated hRIFs, compared to the control group (Figure 6D), and Co‐IP coupled with WB further verified the result (Figure 6E–H). Previous studies have demonstrated that NDP specifically binds to FZD4 to activate β‐catenin–dependent canonical Wnt signaling [48], while SFRP1 can also interact with FZD4 to suppress downstream signaling [49]. Therefore, we performed Co‐IP of anti‐FZD4 and found that HAP‐treatment enhanced the binding between NDP and FZD4, while HAP‐treatment inhibited the binding between SFRP1 and FZD4 in hRIFs, and this effect was abolished by silencing of THY1 (Figure 6F–J). These results indicated that THY1 functioned as a molecular switch in the GSK3α/β–β‐catenin signaling pathway by regulating the interactions among NDP, SFRP1, and FZD4. Co‐staining of THY1 and SFRP1 further verified the interaction dynamics of THY1 and SFRP1 in response to HAP stimulation, as illustrated by HAP crystals promoted the interaction between THY1 and SFRP1 (Figure 6K; Figure S9C). Additionally, we employed a Bimolecular Fluorescence Complementation (BiFC) assay using THY1‐VC155 and SFRP1‐VN173 constructs (Figure 6L). Under normal conditions, hRIFs exhibited minimal Yellow Fluorescent Protein (YFP) fluorescence, indicating limited interaction between THY1 and SFRP1. In contrast, HAP treatment markedly increased YFP fluorescence intensity, suggesting that HAP promotes the interaction between THY1 and SFRP1 in hRIFs (Figure 6M; Figure S9D–F).

To validate whether THY1 competitively interacts with SFRP1 to indirectly activate the β‐catenin pathway, THY1 was over‐expressed in HAP‐treated hRIFs, which further activated GSK3α/β–β‐catenin pathway and upregulated FASLG expression, leading to increased anoikis of hRPSECs, whereas the effect of THY1 over‐expression was abolished by r‐hSFRP1 in a concentration‐dependent manner (Figure 6N–R; Figure S9G). Conversely, the addition of recombinant NDP (r‐NDP) further potentiated the activation of the GSK3α/β–β‐catenin axis and FASLG expression (Figure S9H,I). To determine the HAP binding site on THY1, a series of THY1 deletion constructs were made (Figure 6S,T). HAP co‐precipitation showed that the mutant with deletion of Delta2 segment lost the ability to bind to HAP crystals, indicating that the Delta2 segment of THY1 was responsible for its binding to HAP crystals (Figure 6U). Furthermore, Co‐IP revealed that the THY1 protein lacking the Delta2 segment markedly impaired its binding to NDP and SFRP1, suggesting that the Delta2 segment of THY1 is critical for its interaction with both NDP and SFRP1 (Figure 6V).

### 2.7 GSK3α/β–β‐Catenin–FASLG Axis was Activated in RP and Able to Predict CaOx Stone Recurrence in RP Patients

**FIGURE 7:**
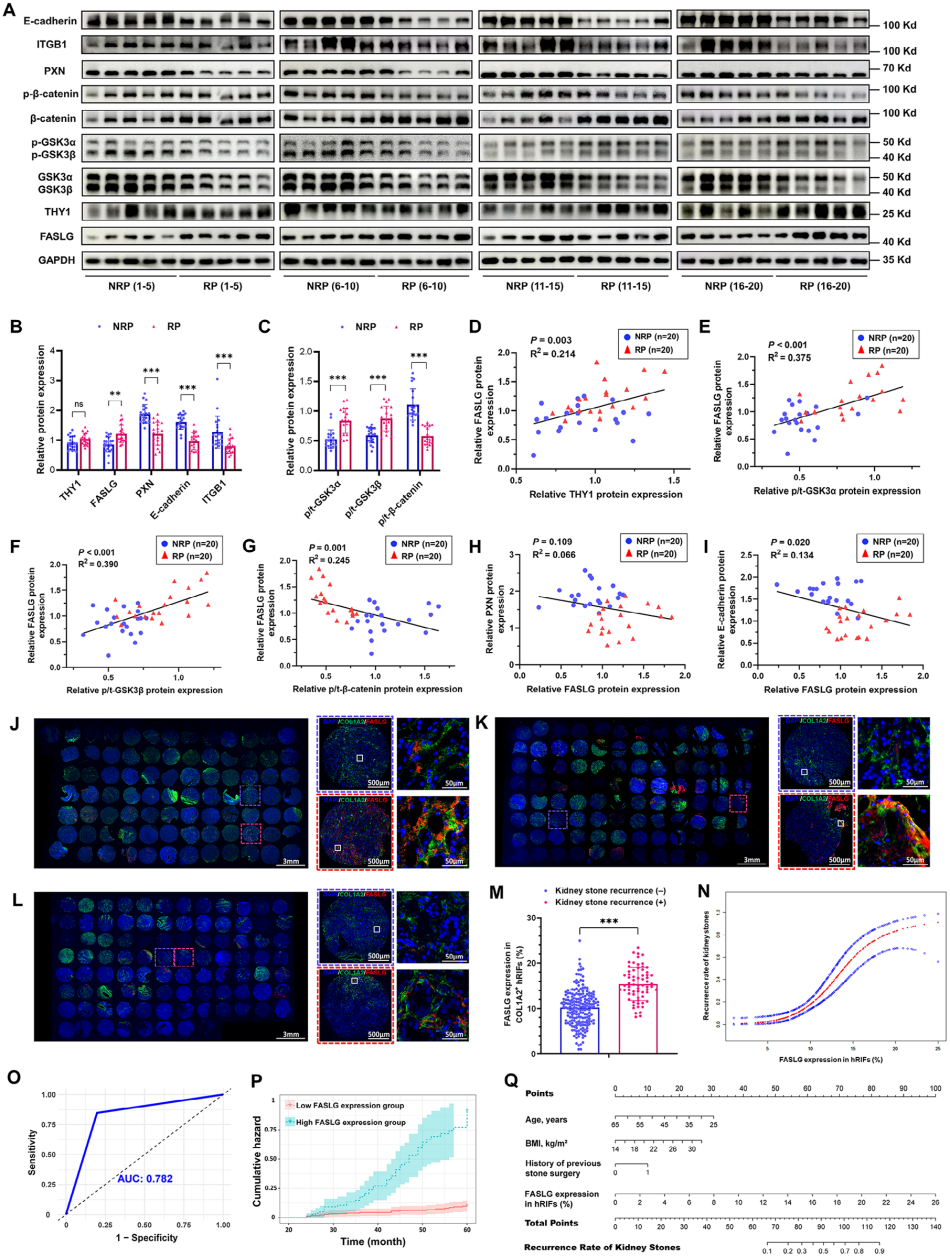
GSK3α/β–β‐catenin–FASLG axis was activated in RP and associated with CaOx stone recurrence. (A‐C) THY1, p/t‐GSK3α/β, p/t‐β‐catenin, FASLG, PXN, E‐cadherin, and ITGB1 were determined by WB in NRP and RP tissues, and WB bands were quantified using ImageJ and normalized to GAPDH; *n* = 20 per group. (D‐G) Correlation analysis of FASLG expression with (D) THY1, (E) p/t‐GSK3α, (F) p/t‐GSK3β, and (G) p/t‐β‐catenin levels. (H‐I) Correlation analysis between FASLG and adhesion‐related proteins (H) PXN and (I) E‐cadherin. (J–L) IF staining of tissue microarrays (TMAs) comprising a total of 270 renal papillary tissue cores from patients with or without kidney stone recurrence. Each panel shows the overall view of an entire TMA slide stained for DAPI (blue), COL1A2 (green), and FASLG (red), alongside representative images of non‐recurrence (blue dashed boxes) and recurrence patients (red dashed boxes). (M) Quantification of FASLG fluorescence intensity in hRIFs between non‐recurrence (*n* = 197) and recurrence (*n* = 66) groups. 7 renal papillary tissue cores were excluded due to unsatisfied quality of IF staining. (N) Smooth curve fitting illustrating the association between FASLG expression in hRIFs and kidney stone recurrence. The red line represents the fitted curve, and the blue shaded area indicates the 95% confidence interval. (O) ROC curve showing the predictive performance of FASLG expression specifically in hRIFs for kidney stone recurrence (AUC = 0.782). (P) Kaplan–Meier survival analysis of kidney stone recurrence in patients stratified by FASLG expression in hRIFs (%) using the optimal cut‐off value determined by the Youden Index method. (Q) Nomogram for predicting the recurrence rate of kidney stones, incorporating significant variables identified through binary logistic regression analysis.

To investigate whether the THY1–GSK3α/β–β‐catenin signaling axis is involved in molecular alterations associated with RP tissues, we assessed the expression levels of key pathway components and adhesion‐related molecules in clinical specimens (Figure 7A–C). While THY1 expression showed no significant difference between RP and NRP tissues, we observed a marked increase in the p/t‐GSK3α/β ratio and a significant decrease in the p/t‐β‐catenin ratio in RP tissues (Figure 7A–C), suggesting activation of the GSK3α/β–β‐catenin signaling pathway in RP. Moreover, FASLG expression was significantly increased in RP tissues (Figure 7A–C), consistent with its role in promoting apoptosis and anoikis. Furthermore, the expression of PXN, E‐cadherin, and ITGB1 was significantly reduced in RP tissues (Figure 7A–C), suggesting a disruption in cell–cell and cell–matrix adhesion, which may facilitate the detachment of hRPSECs and the exposure of RP. Linear regression analysis revealed that FASLG expression was positively correlated with THY1 and p/t‐GSK3α/β levels and inversely correlated with p/t‐β‐catenin (Figure 7D–G), suggesting a potential link between FASLG upregulation and activation of the THY1–GSK3α/β–β‐catenin signaling pathway. Additional correlations among THY1, p/t‐GSK3α/β, and p/t‐β‐catenin further support the involvement of this regulatory axis (Figure S10). Moreover, FASLG expression showed a significant negative correlation with adhesion molecules including PXN and E‐cadherin (Figure 7H,I), implying that elevated FASLG may contribute to impaired cell adhesion within the RP microenvironment.

To further clarify the clinical relevance of FASLG in the context of renal papilla injury and stone recurrence, we performed IF staining on tissue microarrays (TMAs) containing 270 RP tissue cores from 270 CaOx stone patients who completed a 5‐year follow‐up after PCNL (Figure 7J–L). Of these, 7 cores were excluded due to poor staining quality or tissue loss during TMAs processing, leaving 263 patients for downstream analysis (Table S9). FASLG was predominantly localized in hRIFs, and its expression was markedly elevated in tissues from patients with stone recurrence over a 5‐year follow‐up period (Figure 7M). Smooth curve fitting revealed a positive association between the proportion of FASLG‐expressing hRIFs and recurrence events (Figure 7N). ROC analysis demonstrated that FASLG expression in hRIFs exhibited robust predictive power for stone recurrence (AUC = 0.782) (Figure 7O). Kaplan–Meier survival analysis based on the optimal cutoff value (determined by Youden Index from the ROC curve) showed that patients with higher FASLG expression in hRIFs had significantly higher recurrence rates (Figure 7P). Furthermore, multivariable logistic regression analysis showed that age, BMI, previous stone surgery, and FASLG expression in hRIFs were statistically significant predictors of recurrence (Table S10). A nomogram was subsequently constructed to estimate individual recurrence risk, with FASLG expression contributing a substantial portion of the predictive score (Figure 7Q).

### 2.8 Benarthin Targeted THY1 to Disrupt THY1–SFRP1 Interaction, Thereby Inhibiting the GSK3α/β–β‐Catenin–FASLG Axis and Alleviating Anoikis In Vitro and In Vivo

**FIGURE 8:**
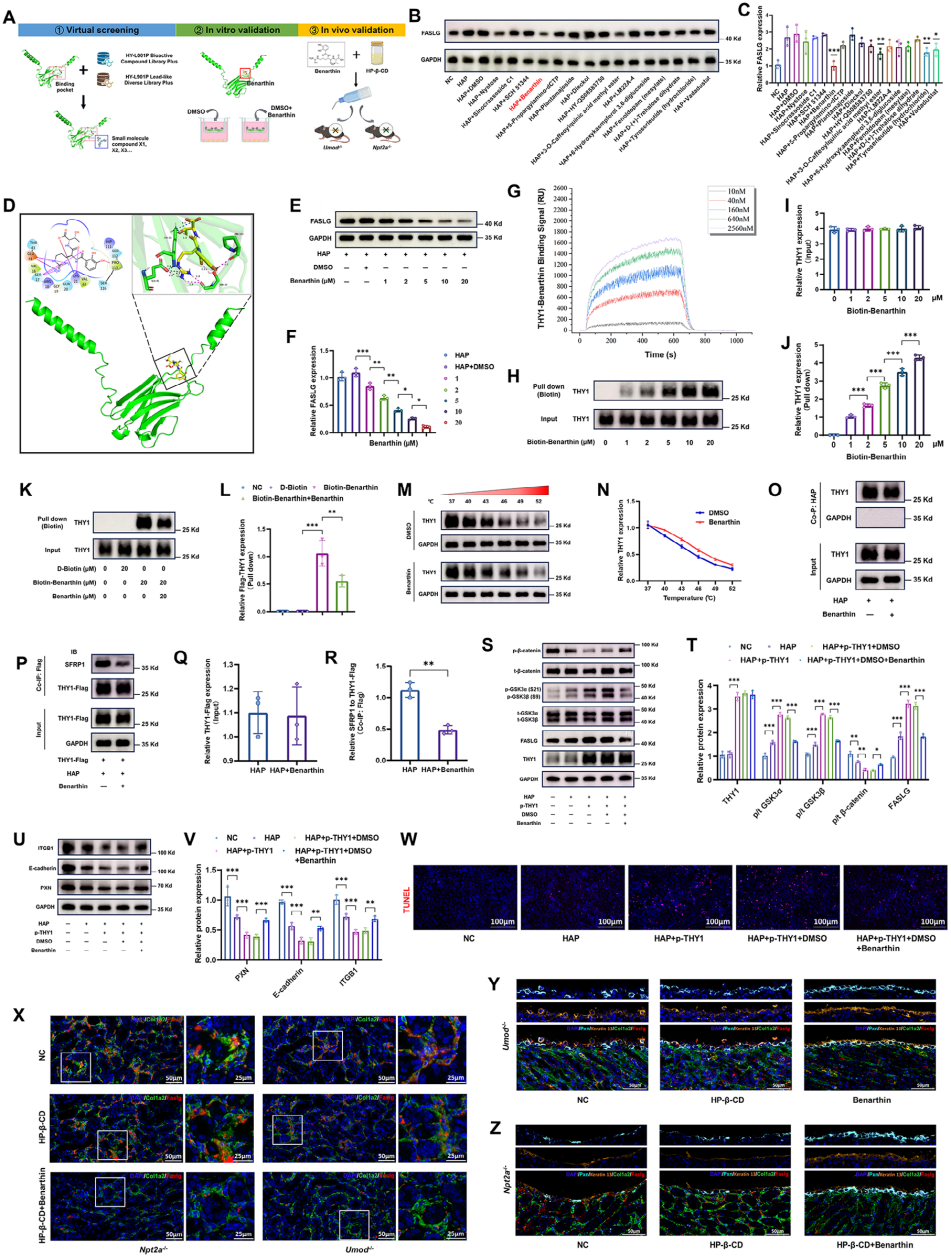
Benarthin inhibited THY1‐SFRP1 binding and GSK3α/β–β‐catenin–FASLG signaling to inhibit anoikis of hRPSECs. (A) Schematic diagram of the experimental design. A virtual screening was conducted using the HY‐L001P and HY‐L901P chemical libraries to identify potential compounds with high binding affinity to the THY1 pocket region. The candidate compounds were selected for further functional experiments in vitro and in RP mouse models. (B, C) These 15 top‐ranked compounds with docking scores were co‐incubated with HAP and hRIFs, followed by WB analysis of FASLG expression; *n*  =  3, *p < 0.05, **p < 0.01, ***p < 0.001 vs. the HAP+DMSO group. (D) Molecular structure of Benarthin and its interaction with THY1. (E, F) WB analysis of FASLG expression in hRIFs treated with HAP and various concentrations of Benarthin (1, 2, 5, 10, and 20 µM); *n*  =  3. (G) Kinetic binding of Benarthin to THY1 was assessed at concentrations of 10, 40, 160, 640, and 2560 nM using Surface Plasmon Resonance (SPR). (H‐J) Cell lysates of hRIFs were incubated with a serial concentration of Biotin‐Benarthin, followed by pulling‐down with streptavidin magnetic beads and WB analysis of THY1; *n*  =  3. (K, L) hRIFs were treated with D‐Biotin, Biotin‐Benarthin, or Biotin‐Benarthin+Benarthin, and Cell lysates of hRIFs were subjected to pulling‐down with streptavidin magnetic beads, followed by WB analysis of THY1; *n* =  3. (M, N) hRIFs were treated with Benarthin, followed by thermal shift assay (CETSA) with heat treatment at 37, 40, 43, 46, 49, and 52°C, followed by WB analysis of THY1; *n* =  3. (O) hRIFs were treated with Benarthin, followed by WB analysis of THY1 in HAP crystal precipitation. (P‐R) hRIFs expressing THY1‐Flag were treated with Benarthin, followed by anti‐Flag IP and WB analysis of THY1‐Flag and SFRP1; *n* =  3. (S, T) WB analysis of signaling pathway proteins in hRIFs treated with HAP, p‐THY1, DMSO, or Benarthin; *n* =  3. (U, V) hRIFs transfected with p‐THY1 plasmids were treated with HAP crystals and Benarthin, then co‐cultured with hRPSECs for 3 days, and the adhesion molecules (PXN, E‐cadherin, and ITGB1) of hRPSECs were determined by WB; *n* =  3; (W) the apoptosis of hRPSECs was determined by TUNEL staining; *n* =  3. (X) IF staining of renal papillary sections for Col1a2‐labeled RIFs (green), and Faslg (red) in *Umod* ^−/−^ and *Npt2a* ^−/−^ mice, which were orally administered water (NC), solubilizing agent (2‐Hydroxypropyl‐β‐cyclodextrin; HP‐β‐CD) or HP‐β‐CD+Benarthin; *n* =  5. (Y, Z) IF staining of renal papillary sections for Pxn (cyan), Keratin‐13 (brown), Col1a2‐labeled RIFs (green), and Faslg (red) from *Umod* ^−/−^ and *Npt2a* ^−/−^ mice, which were orally administered water (NC), HP‐β‐CD or HP‐β‐CD+Benarthin; *n*  =  5.

Given that THY1 played a key role in upregulating FASLG in HAP‐induced hRIFs, and virtual screening provides a high‐throughput strategy to identify protein structure‐based interacting compounds as potential (pro)drugs [50], we performed a THY1 structure‐based virtual screening of small molecule libraries (HY‐L001P and HY‐L901P), which included 107,015 bioactive compounds. The compounds were ranked according to their binding affinity to THY1, and 15 top‐ranked compounds were selected for subsequent experimental screening (Figure 8A; Figure S11 and Table S11). Among these 15 compounds, Benarthin showed the strongest inhibition of FASLG in HAP‐treated hRIFs (Figure 8B,C), and we further found that Benarthin suppressed FASLG in a dose‑dependent manner (Figure 8E,F). Molecular docking analysis indicated that Benarthin bound to the THY1 pocket via seven hydrogen bonds (with PRO113, GLU41, LYS21, and SER17) and two salt bridges involving LYS21 and GLU41(Figure 8D). Surface plasmon resonance (SPR) analysis confirmed high‑affinity binding to THY1 (KD = 5.13E‐08 M) (Figure 8G). Additionally, Biotin pull‐down assays showed the concentration‐dependent binding of Biotin‐Benarthin to endogenous THY1 in hRIFs (Figure 8H–J), and the addition of free Benarthin in hRIFs significantly inhibited the binding of THY1 to Biotin‐Benarthin (Figure 8K,L). Moreover, cellular thermal shift assays (CETSA) demonstrated that Benarthin increased THY1 thermal stability (Figure 8M,N). These findings collectively suggested a specific and direct binding between Benarthin and the THY1 protein.

We further found that Benarthin did not affect the binding of HAP crystals to THY1 (Figure 8O), but inhibited the interaction between THY1 and SFRP1 (Figure 8P–R). Furthermore, Benarthin abolished the activation of the GSK3α/β–β‐catenin pathway and the upregulation of FASLG induced by HAP crystals (Figure 8S,T), which in turn increased the expression of adhesion molecules in hRPSECs, including PXN, E‐cadherin, and ITGB1, thereby alleviating anoikis (Figure 8U,V). Consistently, TUNEL staining confirmed that Benarthin treatment significantly reduced apoptosis in hRPSECs (Figure 8W). To further validate the effects of Benarthin in vivo, Benarthin was administered in drinking water ad libitum to *Umod* ^−/−^ and *Npt2a* ^−/−^ mice. Compared to the NC and solubilizing agent groups, Benarthin treatment markedly reduced Faslg expression in Col1a2‐labeled RIFs in both mouse RP models (Figure 8X), with acceptable biosafety (Figure S12). Furthermore, IF staining showed that Benarthin treatment increased Pxn and enhanced urothelial retention in both *Umod* ^−/−^ and *Npt2a* ^−/−^ mice (Figure 8Y,Z). Together, these findings demonstrated that Benarthin directly targets THY1 to disrupt its interaction with SFRP1, thereby inhibiting the GSK3α/β–β‐catenin–FASLG axis and alleviating RPSECs anoikis both in vitro and in vivo, which supported the potential of Benarthin as a targeted agent against RPSECs anoikis induced by FASLG from HAP‐treated RIFs.

## 3 Discussion

**FIGURE 9:**
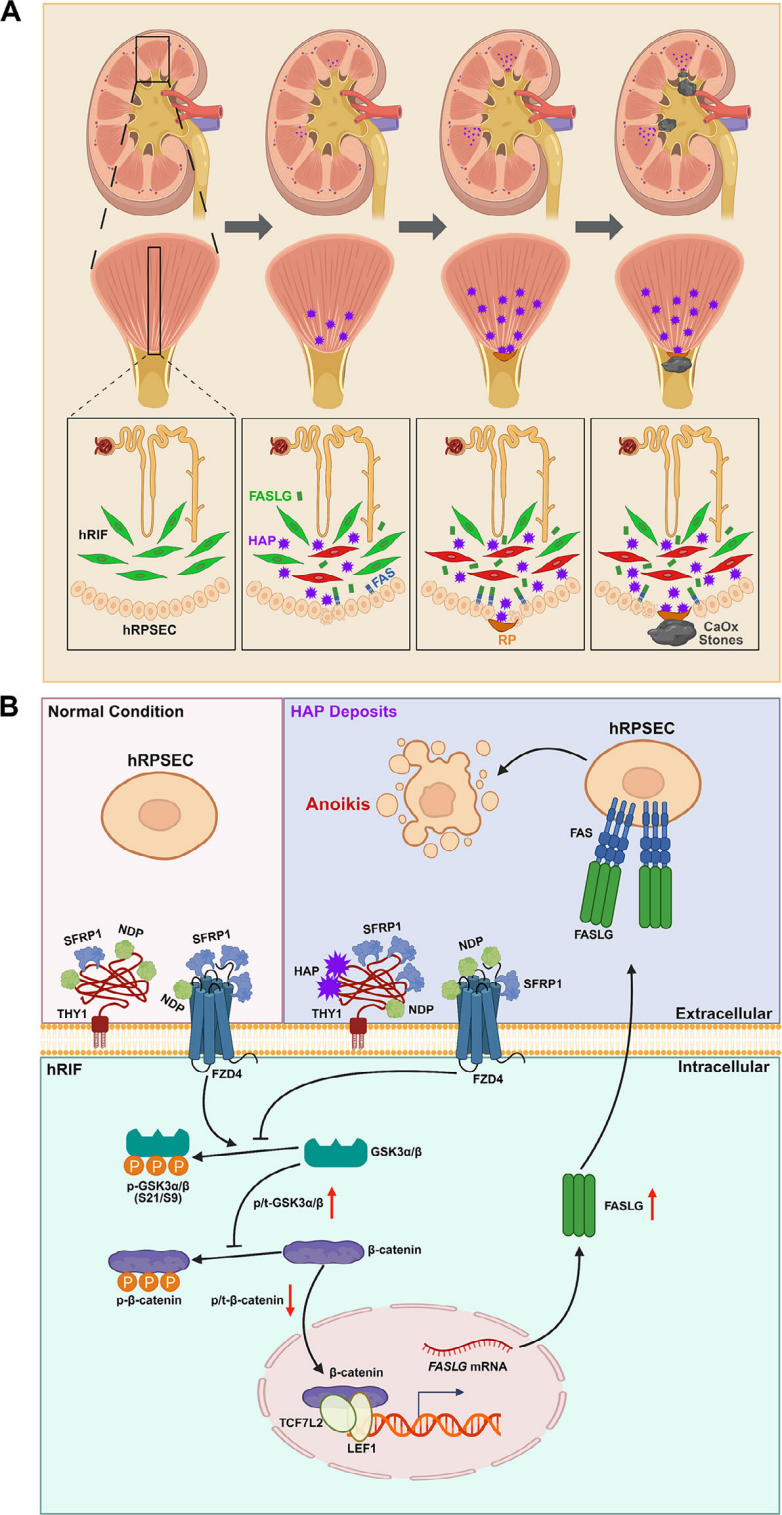
A schematic illustration of the mechanism by which HAP promotes the exposure of RP via hRIF‐mediated urothelial anoikis, creating a nidus for CaOx stone formation. (A) The formation of CaOx stones begins with the deposition of HAP crystals in the renal interstitium surrounding hRIFs. This process induces the upregulation of FASLG in hRIFs. The secreted FASLG binds to its receptor, FAS, on the surface of hRPSECs, leading to epithelial injury and detachment. Concurrently, HAP crystals aggregate and form RP in the subepithelial region. Ultimately, the compromised epithelial barrier exposes the underlying RP to urine, providing a nidus for the attachment and growth of CaOx crystals, culminating in the formation of kidney stones. (B) At the molecular level, HAP crystals bind to THY1 on the hRIF surface, acting as a critical molecular switch. In the presence of HAP, THY1 preferentially binds to and sequesters the secreted inhibitor SFRP1. This sequestration prevents SFRP1 from antagonizing the FZD4 receptor. Consequently, the ligand NDP is able to bind more effectively to FZD4, leading to the activation of its downstream signaling. This cascade activation inhibits the activity of GSK3α/β, thereby reducing the phosphorylation of β‐catenin and preventing its proteasomal degradation. This results in the accumulation of non‐phosphorylated, active β‐catenin in the cytoplasm and its subsequent translocation into the nucleus, where it forms a complex with TCF7L2/LEF1 transcription factors to directly promote the transcription of the *FASLG* gene. The resulting increase in secreted, hRIF‐derived FASLG then drives anoikis in adjacent hRPSECs.

Our study delineated the molecular mechanism responsible for the loss of renal papillary urothelial integrity, a critical prerequisite for the adhesion of CaOx stones to underlying RP (Figure 9A,B). Here, interstitial HAP crystals are found to activate a fibroblast‐driven THY1–FASLG signaling axis, which acts as a paracrine trigger for urothelial anoikis and subsequent plaque exposure. The clinical significance of this cascade is underscored by our finding that FASLG expression independently predicts stone recurrence. Importantly, this insight enabled the identification of Benarthin, a targeted small molecule that effectively preserves urothelial barrier function.

A key finding of our study is the cell‐type‐specific response to HAP stimulation that HAP induced FASLG expression in hRIFs but had a negligible effect on hRPSECs. This profound specificity is underpinned by a unique convergence of molecular machinery and cellular anatomy. We identified the HAP receptor, THY1, which is known to be highly and selectively expressed on renal fibroblasts but is largely absent on tubular epithelia [51, 52]. Furthermore, HAP crystals were extensively deposited throughout the renal papillary interstitium [53], creating an intimate proximity with hRIFs. Established histology confirms that in non‐inflammatory interstitium, hRIFs were the dominant cell type, vastly outnumbering the immune cells like macrophages [8]. This unique anatomical and molecular setup provides the foundation for a paracrine signaling model to clarify how the urothelium is damaged and shed prior to any direct physical contact with the deeply embedded HAP crystals.

The key effector we identified in the loss of renal papillary urothelium, FASLG, is a classic pro‐apoptotic factor that induces cell death by binding to its FAS receptor [54, 55]. In this context, it triggered anoikis, a form of programmed cell death initiated when epithelial cells lose their anchorage to the basement membrane [56, 57]. Our in vitro experiments demonstrated that HAP‐treated hRIFs induce anoikis of hRPSECs, which was corroborated by a histological observation that an inverse relationship between the extent of renal interstitial HAP deposition and the integrity of the overlying urothelium. Moreover, as HAP deposits increased, interstitial FASLG expression escalated while the epithelial adhesion marker concurrently diminished. These findings supported our proposed paracrine destruction model to partially explain the progressive loss of epithelial integrity caused by the renal interstitial HAP deposition. Of note, the molecular etiology of this event is different from the canonical anoikis machinery. Physiologically, urothelial survival depends on integrin‐mediated anchorage, which sustains pro‐survival signaling, particularly the Focal Adhesion Kinase (FAK)–PI3K/Akt axis [57, 58]. Classical anoikis is therefore characterized by the withdrawal of these essential survival cues following mechanical detachment. Distinct from this deprivation model, our study reveals that the urothelium is targeted by FASLG secreted from HAP‐stimulated fibroblasts. This indicates that the THY1‐FASLG axis operates not merely through the failure of homeostatic support, but as a dominant pro‐apoptotic signaling cascade that drives epithelial destruction.

Another key finding of our study is this remarkable target‐cell specificity: the FASLG signal selectively destroys the papillary urothelium while leaving the adjacent tubular epithelia largely intact. First, the urothelium is intrinsically primed for this apoptotic signal, since the FAS receptor is constitutively expressed in healthy urothelial tissue [59, 60], a well‐documented feature that allows for the rapid elimination of compromised cells to maintain barrier integrity. In contrast, FAS receptor is expressed in human renal tubule at very low levels [61, 62] and reportedly undetectable by flow cytometry in mice [54]. Second, the renal tubular epithelium possesses a remarkable regenerative capacity, driven by the plasticity of its own resident cells [63, 64]. After damage, surviving tubular cells themselves act as facultative progenitors, proliferating to rapidly replace lost cells and restore tubular integrity [63]. Furthermore, recent findings suggest that damaged tubular cells may not be shed but instead persist by transitioning into a stable, undifferentiated state [22], whereas the urothelium is a remarkably quiescent tissue [65, 66]. This inherent quiescence, coupled with its reliance on a limited progenitor pool for repair, results in a sluggish regenerative response, rendering the tissue exceptionally vulnerable to widespread and synchronous cell loss [67]. Third, the pelvis acts as a basin, leading to prolonged, high‐concentration contact of the signal with the urothelium. In contrast, the rapid, continuous flow of fluid through the nephron limits the contact time and effective dose of the signal on tubular epithelium [68, 69].

Far from being inert mineral deposits, HAP crystals are now recognized as potent bioactive effectors that engage with host cells to drive pathology. Extensive prior work has established that at the bio‐mineral interface, HAP crystals elicit profound cytotoxic and pro‐inflammatory insults in diverse cell types, including renal tubular epithelia [70, 71] and VSMCs [72, 73]. Beyond inducing overt apoptosis and necrosis, HAP crystals can also trigger pathological phenotypic switching, most notably driving the osteogenic differentiation of VSMCs [74], a key event in vascular calcification. A large body of work has established that CaOx crystals can inflict a cascade of cytotoxic insults on renal epithelial cells, including oxidative stress and membrane rupture, thereby promoting a vicious cycle of injury and crystal retention that fuels stone growth [75, 76]. However, a fundamental paradox emerges when attempting to position these CaOx‐centric findings as the explanation for the genesis of idiopathic stone disease. Several decades of seminal work have established that the initiating lesion is not CaOx, but rather the extensive HAP deposition deep within the papillary interstitium [8, 13]. This fundamental histopathology reveals a critical spatiotemporal disconnect: CaOx crystals are a secondary, luminal deposit that attaches only after the primary interstitial HAP has matured and the overlying urothelial barrier has been breached. It is precisely this knowledge gap that our study confronts and resolves. By modeling the interaction between interstitial HAP and the resident hRIFs it directly encounters, we therefore define the primary pathogenic event: a fibroblast‐driven destruction of the urothelial barrier, which culminates in the critical exposure of the subepithelial RP to the urinary milieu.

Our prior work revealed that hRIFs shaped an osteogenic‐like microenvironment and trigger interstitial HAP deposition [23]. Integrating our current study, these results indicated a pathogenic feedback loop as the core driver for RP progression, as demonstrated by that fibroblast‐driven mineralization leads to HAP deposition, which in turn activates fibroblasts to drive anoikis of urothelium. Intriguingly, our findings echoed what has been found in the progression of atherosclerotic plaques. On one hand, this model of active mineralization mirrors vascular calcification [77]. In the vessel wall, VSMCs undergo a profound phenotypic switch into osteoblast‐like cells [77, 78], serving as the primary sites for HAP nucleation [79, 80]. On the other hand, hRIFs‐derived FASLG induced anoikis in the overlying urothelium, which is conceptually analogous to atherosclerotic plaque rupture. In vulnerable atherosclerotic plaques, the stability of the protective fibrous cap is actively undermined through a complex interplay between resident and recruited cells. Activated macrophages and phenotypically switched VSMCs, functioning as “aggressor” cells, deploy a multifactorial strategy to dismantle this barrier. Primarily, they secrete pro‐apoptotic members of the TNF superfamily, which directly trigger apoptosis in both the cap's VSMCs and the overlying endothelial cells, thereby compromising the barrier's cellular integrity [81, 82]. In parallel with this direct cellular attack, these aggressor cells release a cocktail of destructive enzymes, most notably matrix metalloproteinases (MMPs). These enzymes not only degrade the collagenous framework of the cap, leading to its physical weakening [83], but also critically disrupt the cell‐matrix adhesions essential for cell survival, thereby inducing apoptosis [84]. Given upregulated MMP7 and MMP9 in RP tissues revealed by a spatial molecular landscape study [22], further studies are warranted to investigate MMPs’ role in the anoikis of renal papillary urothelium.

Benarthin was first identified in the early 1990s as a natural product from Streptomyces xanthophaeus and characterized as a potent inhibitor of pyroglutamyl peptidase [85]. Foundational studies subsequently defined its chemical structure and, through structure‐activity relationship analyses, pinpointed the core pharmacophore essential for this function: the N‐terminal catechol group and the central L‐arginine residue [86, 87]. Research has re‐identified Benarthin as a catechol‐type siderophore involved in bacterial iron acquisition [88]. Furthermore, it is now understood to function as a fundamental monomer for assembling larger, more potent siderophores such as Streptobactin [89]. Our research dramatically expands the biological profile of this molecule. We have identified a novel and therapeutically relevant function for Benarthin: targeting the cell surface protein THY1 on RIFs to potently suppress HAP‐induced FASLG expression. This newly discovered activity is underpinned by a highly stable interaction, as our docking simulations revealed a dense network of seven hydrogen bonds and two salt bridges anchoring the compound to THY1. Crucially, beyond its molecular efficacy, our study provides the first comprehensive evidence of Benarthin's safety profile in a mammalian model. We demonstrated that long‐term administration elicited no pathological changes in major organs. Furthermore, serum biochemical markers remained within physiological ranges across both acute (12–96 h) and chronic phases (3 m), indicating excellent tolerability. Collectively, the novel mechanism, high binding affinity, and robust safety profile position Benarthin as a promising lead compound. Moving forward, the translational path will prioritize optimizing its pharmacokinetic properties, particularly its renal distribution and oral bioavailability, to facilitate its development as a potential therapy for kidney stone prevention.

We acknowledge several limitations that provide avenues for future research. First, our analyses could not resolve the potential heterogeneity of RIFs, leaving it unclear whether a specific subpopulation drives the observed FASLG expression. Future single‐cell and spatial transcriptomics are required to pinpoint the precise cellular source of this signal. Second, while our 3D co‐culture system revealed key cellular interactions, it did not encompass the full complexity of the renal microenvironment, which includes crosstalk with other resident cells. Third, regarding the mechanistic dissection, we acknowledge that as a GPI‐anchored protein, THY1 signaling likely involves upstream non‐kinase initiators, such as lipid raft reorganization or calcium influx [90]. Nevertheless, our finding that specific inhibition of the GSK3‐β‐catenin axis reversed over 90% of the HAP‐induced phenotype indicates that these diverse initiating signals ultimately converge upon this canonical kinase cascade to drive pathology. Fourth, although β‐catenin is known to cooperate with diverse transcription factors (e.g., FOXO, SOX family members) under stress conditions, we did not exhaustively screen every potential partner. Nevertheless, the complete suppression of FASLG expression following specific disruption of the β‐catenin‐TCF/LEF interaction (via iCRT3) confirms that the TCF/LEF complex serves as the key functional partner in this specific context. Fifth, while our genetically modified mouse model successfully mimicked key aspects of renal interstitial calcification, inherent anatomical and physiological differences between mice and humans limit the direct extrapolation of our findings. Sixth, the predictive power of our model was constrained by the modest sample size and the 5‐year follow‐up period of our cohort, which may not fully capture long‐term recurrence patterns. Its robustness and generalizability must therefore be validated in a larger, multi‐center longitudinal study. Finally, while Benarthin demonstrated significant efficacy and an excellent safety profile in our animal models, we acknowledge that detailed pharmacokinetic properties and metabolic stability were not fully characterized in the current study. Future investigations focusing on the comprehensive pharmacological profiling and potential off‐target effects are essential to pave the way for its clinical translation.

## 4 Conclusions

Our study demonstrated a previously unidentified pathological feature of RP that renal interstitial HAP deposits indirectly compromise the urothelial barrier to facilitate the exposure of RP to urine. Mechanistically, HAP crystals activated the THY1–SFRP1/NDP–GSK3α/β–β‐catenin signaling axis in hRIFs, inducing hRIFs‐derived FASLG to drive anoikis of hRPSECs, which ultimately disrupts epithelial integrity and creates a nidus for the subsequent adhesion of CaOx crystals. Coherently, FASLG expression of hRIFs within RP tissues served as a robust biomarker for predicting CaOx stone recurrence. Moreover, Benarthin, a small‐molecule compound, is identified to effectively block this pathological process by directly targeting THY1, thereby protecting epithelial integrity. Collectively, these findings identify a novel pharmacological target and offer a new therapeutic strategy for the prevention of recurrent CaOx stones.

## 5 Methods

### 5.1 Sex as a Biological Variable

Our study examined samples from both male and female patients, as well as both male and female animals in our experimental models. Similar findings are reported for both sexes.

### 5.2 Clinical Samples

Prior to sample collection, informed consent was obtained from each participant, and the study protocol was approved by the Xiangya hospital's Ethics Committee (Approval No. 201603035; 202103089).

To obtain intact renal papillae for enough molecular data, human renal papilla samples were obtained from patients undergoing nephrectomy for renal malignancies at Xiangya Hospital between April 2021 and March 2024. Participants were enrolled based on the following criteria: 1) preserved renal function; 2) absence or only minimal hydronephrosis; 3) no evidence of renal atrophy on computed tomography (CT); and 4) renal papillae located at least 3 cm from the tumor margin were available for excision. Samples were classified into two groups. NRP were defined by a negative history of urolithiasis and the absence of calcium deposition in the papillary interstitium, as confirmed by Von Kossa staining. RP‐positive tissues, on the other hand, were collected from patients with CaOx nephrolithiasis or a documented history of CaOx stone formation, and the presence of interstitial calcium deposits was verified by histological staining. 26 pairs of NRP and RP specimens from 52 patients with renal malignancies were included for analysis, and the clinical information was detailed in Table S12.

To analyze RP‐associated risk factors for recurrence in CaOx stone patients following percutaneous nephrolithotomy (PCNL), RP tissues were obtained with biopsy forceps from CaOx stone formers during PCNL as performed in our previous study [91]. 270 RP tissues from 270 patients with both stone‐free status following PCNL and 5‐year follow‐up information were included for constructing tissue microarrays (TMAs). The stone‐free status following PCNL was defined as no residue fragments >3 mm examined by Kidney‐Ureter‐Bladder Radiography (KUB)/CT, as described in our previous study [92]. Stone recurrence was defined as newly detected calculi ≥5 mm on ultrasound/KUB/CT in patients with previously stone‐free status after PCNL [93]. The clinical information of patients included for final analysis was provided in Table S9.

### 5.3 Laser Capture Microdissection (LCM)

For each RP tissue, 15‐µm‐thick FFPE sections were obtained and HAP deposits were visualized with Von‐Kossa staining, and then mounted on a PEN membrane slide specialized for laser microdissection. The calcified and non‐calcified regions were micro‐dissected using a CELLCUT PLUS Laser Microdissection System (Nikon ECLIPSE Ti2, Japan). To collected sufficient tissues for downstream MS, 20–25 sections from a single RP sample were isolated.

### 5.4 Cell Isolation and Culture

Based on our previously studies [23, 91], hRIFs were obtained from the renal medulla and papillae. In brief, minced renal tissues were dissociated with 1 mg mL^−1^ Collagenase II (Sigma–Aldrich, USA) and subsequently filtered through a 70 µm cell strainer. The retained tissue fragments were cultured for 10–14 days to expand the renal interstitial fibroblasts. The initial mixed cell population exhibited both a cobblestone morphology, indicative of epithelial cells, and a spindle‐shaped morphology, representative of fibroblasts. The cells were serially passaged until a pure, 100% confluent fibroblast culture was achieved, leveraging their superior adherence and growth advantage [94, 95]. CLCNKA^+^ hTECs and hRVECs were isolated from renal papillary tissue using MicroBeads (Miltenyi Biotec, Germany) coated with specific antibodies: anti‐CLCNKA for CLCNKA^+^ hTECs and anti‐CD34 for hRVECs. CLCNKA^+^ hTECs were included because previous studies have reported calcium deposits in regions adjacent to the thin ascending limb of Henle's loop, which is marked by CLCNKA expression [28]. This spatial correlation suggests a potential involvement of these cells in the local response to crystal deposition, warranting their inclusion in our investigation. Considering the different feature of the urothelium lining the pelvis and the epithelium lining the papilla [96], we isolated the surface epithelial cells lining the papilla by scraping the renal papillary mucosa hRPSECs [97]. Cell identity was confirmed via IF with the corresponding marker. The purity of hRIFs were identified with the positive expression of COL1A2 and Vimentin, and the negative expression of E‐cadherin based on previous studies (Figure S3C) [91, 98]. hRPSECs were identified by positive E‐cadherin and Keratin‐13 (Figure S3D), CLCNKA^+^ hTECs by the positive CLCNKA (Figure S3E), and hRVECs by the positive CD34 staining (Figure S3F). All primary cells between passages 3 and 6 were used for downstream experiments. Cells were maintained in DMEM (Servicebio, China) supplemented with 10% fetal bovine serum (FBS, Beyotime, China), 100 U mL^−1^ penicillin, and 100 µg mL^−1^ streptomycin (Servicebio, China) in a humidified incubator at 37 °C with 5% CO_2_.

### 5.5 3D Co‐Culture of hRIFs and hRPSECs

hRIFs were first transfected with plasmids encoding GFP for fluorescent labeling. For gene knockdown experiments, the hRIFs were subsequently infected with lentiviruses carrying either a control short hairpin RNA (Len‐sh‐ctrl) or shRNA targeting FASLG (Len‐sh‐FASLG). Matrigel (Corning, USA) was kept on ice to maintain its liquid state and then mixed with the indicated hRIFs in suspension. The mixture was plated and incubated at 37°C for 30–60 min to allow solidification. hRPSECs transfected with a Cherry fluorescent protein‐expressing plasmid were then seeded on top of the solidified gel. After 3 days of co‐culture, the gels were gently rinsed with PBS to remove non‐adherent hRPSECs. HAP crystals were labeled with OSTEO680 (OsteoSense 680EX, Revvity, USA). 3D confocal fluorescence imaging (Zeiss LSM 900 with Airyscan 2, Germany) was performed to visualize cell localization and adhesion within the matrix.

### 5.6 Transwell Co‐Culture of hRIFs and hRPSECs

hRIFs were seeded in the lower chamber of a 0.4 µm pore size Transwell insert (Corning, USA) and divided into four groups: untreated (NC), HAP‐treated, HAP + control shRNA (Len‐sh‐ctrl), and HAP + FASLG‐targeting shRNA (Len‐sh‐FASLG). After the hRIFs reached approximately 50–60% confluence, hRPSECs were seeded into the upper chamber of the Transwell insert, allowing for indirect co‐culture without direct cell‐cell contact. The co‐culture system was maintained for 3 days under standard conditions (37°C, 5% CO_2_). Following co‐culture, the Transwell membranes were carefully removed, fixed in 4% paraformaldehyde, and embedded in paraffin. Sections were prepared using a microtome, followed by hematoxylin and eosin (H&E) staining. The number and adhesion status of hRPSECs on the upper membrane surface were examined under a light microscope.

### 5.7 Co‐Immunoprecipitation (Co‐IP)

Magnetic protein A/G beads (MCE, USA) were conjugated with the target‐specific antibody at room temperature for 1 h. Cell lysates were incubated with the antibody‐bead complex overnight at 4°C. After washing with lysis buffer, the bound proteins were analyzed by immunoblotting or mass spectrometry, as described in Supporting Materials.

### 5.8 Co‐Precipitation of HAP Crystals and Proteins

Co‐precipitation (Co‐P) of HAP crystals and proteins was performed according to a previous study where CaOx crystal‐binding membrane proteins were investigated [99]. Briefly, membrane proteins of hRIFs were extracted using the Cell Membrane and Cytoplasmic Protein Extraction Kit. (Beyotime, China), followed by incubation with HAP crystals (diameter: 500 nm) in Tris‐HCl buffer (pH = 7.4; 100 mM NaCl; 1% PMSF) at 4°C overnight. Meanwhile, SiO_2_ crystals with similar diameter were used as the negative control to determine the unspecific‐binding proteins. The crystal‐protein co‐precipitations were collected by a centrifugation (3000 rpm; 4°C) for 5 min and the unbound membrane proteins were discarded. Thereafter, the crystal‐protein co‐precipitations were washed 3 times with 4 mM EDTA in PBS. The crystal‐binding proteins were then eluted by Laemmli buffer, followed by separation using sodium dodecyl sulfate‐polyacrylamide gel electrophoresis (SDS‐PAGE) for mass spectrometry (Thermo Scientific, USA) or WB.

### 5.9 Chromatin Immunoprecipitation

Chromatin immunoprecipitation (ChIP) were conducted using the EpiQuik ChIP Kit (Epigentek, USA), following previously validated procedures [100]. The enriched DNA fragments were analyzed by quantitative PCR (q‐PCR) and confirmed by electrophoresis on 1% agarose gels. Anti‐RNA polymerase II and primers targeting the GAPDH promoter served as positive controls, while normal mouse IgG and H_2_O were used as negative controls for antibodies and specific primers. Primer sequences for amplification of FASLG promoter regions containing predicted TCF7L2 or LEF1 binding motifs were listed in Table S13.

### 5.10 Bimolecular Fluorescence Complementation

Bimolecular fluorescence complementation (BiFC) was performed as described in a previous study [101]. Briefly, hRIFs were co‐transfected with pBiFC‐THY1‐VC155 and pBiFC‐SFRP1‐VN173 plasmids (Addgene, USA) using the lip3000 reagent (Invitrogen, USA). The negative control was set as the co‐transfection of pBiFC‐THY1‐VC155 and pBiFC‐VN173 plasmids or pBiFC‐VC155 and pBiFC‐SFRP1‐VN173 plasmids (Addgene, USA). After transfection 24 h, hRIFs were treated with or without HAP crystals, and fluorescence signals of YFP were captured by a confocal laser scanning microscopy (Zeiss LSM 900 with Airyscan 2, Germany).

### 5.11 Immunofluorescence

Immunofluorescence (IF) was performed on ECM, hRIFs, and paraffin‐embedded human and mouse renal tissues using previously optimized protocols [23, 100]. For tissue sections, heat‐mediated antigen retrieval and peroxidase blocking were followed by sequential multiplex labeling based on tyramide signal amplification (TSA). Primary antibodies and HRP‐conjugated secondary antibodies were applied in cycles, with antibody stripping between rounds. Fluorophore‐conjugated tyramide reagents were used for target visualization, and nuclei were counterstained with DAPI. Imaging and colocalization analysis were carried out using high‐resolution slide scanning and confocal microscopy.

### 5.12 Animals

Animal experiments were approved by the Institutional Experimental Animal Committee of Central South University and Xiangya hospital (Approval No. CSU‐2022‐0454, XY20240303001). *Umod* ^+/–^ mice [30, 31] and *Npt2a* ^+/–^ mice [32, 33] were reported by several studies to establish mouse models of RP. For *Umod* ^+/–^ mice, the Umod gene (NCBI Reference Sequence: NM_009470) is located on mouse chromosome 7, and eleven exons are identified, within which exon 3–7 were selected as target site to be deleted. For *Npt2a* ^+/–^ mice, the Slc34a1 gene (NCBI Reference Sequence: NM_011392) is located on mouse chromosome 13, and thirteen exons are identified, within which exon 9–10 were selected as target site to be deleted. For *Faslg* ^+/flox^ mice, the Fasl gene (NCBI Reference Sequence: NM_010177.4) is located on mouse chromosome 1. The gene consists of four exons, with the ATG start codon in exon 1 and the TAA stop codon in exon 4. Exon 3–4 was selected as the CKO region. Deletion of this region induces a frameshift mutation, leading to the loss of function of the mouse Fasl gene. To engineer the targeting vector, 5' and 3' homologous arms flanking the CKO region were generated by PCR using the BAC clone RP23‐373P17 as a template. Cas9 protein, guide RNAs, and the targeting vector containing the exon flanked by loxP sites were co‐injected into fertilized C57BL/6J eggs for CKO mouse production. The resulting F0 pups were genotyped by PCR followed by sequencing analysis to confirm correct integration. *Col1a2‐CreERT* ^tg/+^ mice, expressing tamoxifen‐inducible Cre recombinase under the pro‐α2(I) collagen (Col1a2) promoter, were used to achieve conditional gene deletion of RIFs upon tamoxifen administration, as described in our previous study [23]. All mice were purchased from Cyagen Biosciences Co., Ltd. (China). The detail mating strategy was provided in Supporting Materials. Mice were genotyped by PCR amplification of DNA extracted from tail clippings (Table S14).

### 5.13 Virtual Screening

For virtual screening of compounds interacting with THY1, 2D structures of 107 015 compounds from two MCE libraries (HY‐L001P Bioactive Compound Library Plus and HY‐L901P Lead‐like Diverse Library Plus) and 3D structure of THY1 (AlphaFold ID: AF‐P04216‐F1, 1–161) were submitted to Schrödinger’ docking module level for molecular docking, including High Throughput Virtual Screening (HTVS) module; Standard Precision (SP) module; Extra Precision (XP) module; molecular mechanics with generalized born surface area (MMGBSA) and ligand strain energy calculations. Protein ligand interaction fingerprints (PLIF) and structure diversity was also performed. The illustration of Molecular docking structures was generated using PyMOL software (Schrödinger, LLC, USA). The detail process of virtual screening was provided in Supporting Methods.

### 5.14 Surface Plasmon Resonance Assay

Surface Plasmon Resonance (SPR) was utilized to assess the interaction between THY1 and Benarthin. Briefly, a biomolecular microarray chip was integrated with a plastic flow channel for loading samples. Recombinant human THY1 protein was prepared in PBS containing 0.1% Tween 20 (pH 7.4) as the running buffer, and 10 mM glycine‐HCl solution (pH 2.0) served as the regeneration buffer. The binding kinetics were assessed by injecting the protein solution at a flow rate of 0.5 µL min^−1^ for 10 min (association phase), followed by running buffer for 6 min (dissociation phase). Subsequently, the regeneration buffer was flowed at 2 µL min^−1^ for 200 s. All binding parameters were recorded and processed using the manufacturer's SPR data analysis platform (Plexera SPR Data Analysis Module).

### 5.15 Statistical Analysis

All experiments were independently repeated a minimum of three times. Categorical data were evaluated using either the Chi‐square test or Fisher's exact test, depending on the data distribution and sample size. Continuous variables were expressed as the mean ± standard deviation (SD) and compared using either one‐way analysis of variance (ANOVA) or an unpaired Student's t‐test, as appropriate. Correlations between two continuous or ordinal variables were determined using Spearman's rank correlation coefficient. Statistical significance was defined as a two‐sided *p* value ≤ 0.05. All statistical tests were conducted using GraphPad Prism version 9 (USA) and R version 4.3.1. In all analyses, significance levels are denoted as follows: *p* < 0.05 (*), *p* < 0.01 (**), and *p* < 0.001 (***).

### 5.16 Study Approval

This study was conducted in accordance with ethical guidelines and was approved by the Institutional Experimental Animal Committee of Central South University (Approval No. CSU‐2022‐0022; CSU‐2022‐0454) and Xiangya Hospital Ethics Committee (Approval No. 201603035; 202103089; XY20240303001). Prior to surgery, all patients provided written informed consent for participation in the study, including the collection of biological samples and the anonymized use of clinical data for research and publication purposes.

## Author Contributions

**Z.Z**., **M.L**., and **H.C**. conceived the study; **M.L**., **M.W**., **M.G**., **Y.L**., **M.Y**., **Z.L**., and **Y.W**. performed the biochemical experiments; **M.L**., **M.G**., **Z.L**., **Y.C**., **J.C**., and **C.H**. performed the animal experiments. **M.L**., **Z.C**., and **F.Z**. performed the pathological experiments; **M.L**., **Z.Z**., and **H.C**. analyzed the data; **M.L**. and **Z.Z**. wrote the manuscript; **H.C**. and **F.Z**. revised the manuscript; **Z.Z**., and **H.C**. supervised this work. All authors discussed the results and approved the final manuscript.

## Conflicts of Interest

The authors declare no conflicts of interest.

## Supporting information




**Supporting File 1**: advs75114‐sup‐0001‐TablesS1.xlsx.

**Supporting File 2**: advs75114‐sup‐0002‐SuppMat.docx.

## Data Availability

The datasets supporting the findings of this study are provided within the article and its supplementary materials. Any further data or materials are available from the corresponding authors upon reasonable request.
