# Supplementary material for: FASLG Derived from Fibroblasts in Hydroxyapatite‐Rich Microenvironment Induces Urothelial Anoikis to Trigger Randall's Plaque Exposure
Source: Adv Sci (Weinh). 2026 Apr 2;13(34):e21605. doi: 10.1002/advs.202521605 (PMC13285130; doi:10.1002/advs.202521605)
Supplement: Supplementary file 2 — Supporting File 2: advs75114‐sup‐0002‐SuppMat.docx. [file ADVS-13-e21605-s001.docx]

**Supplementary materials for “FASLG derived from Fibroblasts in Hydroxyapatite-Rich Microenvironment induces Urothelial Anoikis to Trigger Randall's Plaque Exposure”**

**Supplementary Methods**

**Cell transfection**

A recombinant lentivirus targeting FASLG (Len-sh-FASLG; GenePharma, China) was used to achieve stable knockdown of FASLG. siRNAs targeting FAS and THY1, a THY1 overexpression plasmid (p-THY1), and a custom siRNA library (RiboBio, China) targeting membrane-associated proteins (including CD59, THY1, CD63, LAMP1, PODXL, CD44, ITGB1, ANPEP, BSG, and NPTN) were transfected into hRIFs using Lipofectamine 3000 (Invitrogen, USA). Additionally, hRIFs were transfected with reconstituted plasmids encoding HA-tagged or Flag-tagged THY1 (GenePharma, China) using Lipofectamine 3000. All si-RNA and sh-RNA target sequences are provided in **Table S15**.

**Reverse transcription and qRT-PCR**

Total RNA was isolated from hRIFs using the SteadyPure Rapid RNA Extraction Kit (AG, China). The obtained RNA was then reverse transcribed into complementary DNA (cDNA) using the Evo M-MLV reverse transcription premix kit (AG, China). Quantitative real-time PCR (qRT-PCR) was subsequently conducted with a fluorescence-based qPCR system (Eppendorf, Germany), utilizing the SYBR Green Pro Taq HS Premix qPCR Kit II (AG, China). Gene expression levels were normalized against GAPDH and analyzed using the 2^−ΔΔCt^ method. Primer details are listed in **Table S16** of the Supporting Information.

**Western Blot (WB)**

WB was performed as previously described^[1,2]^. Protein signals were detected by a chemiluminescence imaging system (Amersham, UK). Information on primary and secondary antibodies is available in **Table S17** of the Supplementary Information. Band intensities were quantified using ImageJ and normalized to GAPDH, with results expressed as fold change relative to the control group.

**Gene Set Enrichment Analysis (GSEA)**

GSEA is a computational method that determines whether a predefined set of genes shows statistically significant, concordant differences between two biological states by analyzing their distribution in a ranked list of genes^[3]^. For the specific analysis of anoikis, a custom gene set was constructed. This set was generated by integrating genes related to apoptosis, cell-cell adhesion, and focal adhesion. The gene list was compiled based on previously reported literature^[4]^ and the GSEA website, resulting in a non-redundant collection of 258 genes (**Table S2**) used for the enrichment analysis.

**Reactive oxygen species (ROS) detection**

Intracellular ROS levels were measured using the ROS Assay Kit (Beyotime, China). After removing the culture medium, 1 mL of 10 μM DCFH-DA working solution was added to the cells, followed by incubation at 37 °C for 20 minutes in a humidified incubator. Cells were then washed three times with serum-free medium to thoroughly remove any extracellular DCFH-DA. ROS levels were observed using a fluorescence microscope (Leica TCS SP8 X, Germany) or quantified by flow cytometry (NL-3000 Full Spectrum Flow Cytometer, Cytek, USA) with excitation at 488 nm and emission at 525 nm.

**TUNEL assay for apoptosis detection**

Apoptosis was assessed using the One Step TUNEL Apoptosis Assay Kit (Beyotime, China). Cells were washed once with PBS and fixed with 4% paraformaldehyde for 30 minutes. After washing with PBS, the cells were permeabilized with PBS containing 0.3% Triton X-100 at room temperature for 5 minutes. Subsequently, 100 μL of TUNEL detection solution was added to the cells, which were then incubated at 37°C in the dark for 60 minutes. Cells were washed three times with PBS, followed by mounting with anti-fade mounting medium. Fluorescence microscopy (Leica TCS SP8 X, Germany) was used to observe apoptotic cells.

**Cell cycle analysis**

Cell cycle distribution was analyzed using the Cell Cycle and Apoptosis Analysis Kit (Beyotime, China). Cells were digested with trypsin, centrifuged at 1000g for 3-5 minutes, and fixed in ice-cold 70% ethanol at 4°C for 2 hours. After centrifugation, cells were stained with 0.5 mL propidium iodide (PI) solution and incubated at 37°C for 30 minutes in the dark. Cell cycle analysis was performed by a NL-3000 Full Spectrum Flow Cytometer (Cytek, USA), detecting red fluorescence at 488 nm excitation.

**Scanning and transmission electron microscopy (SEM and TEM)**

As previously described^[5]^, SEM and TEM imaging were conducted using the Quanta-200 and Tecnai G2 F20 microscopes (FEI, USA), respectively. For immunogold labeling, ultrathin sections placed on nickel grids were blocked with 1% bovine serum albumin and incubated with specific primary antibodies; normal IgG was used as a negative control. Gold-conjugated secondary antibodies (Servicebio, China) were applied to visualize antigen-antibody complexes. After labeling, sections were counterstained using 2% uranyl acetate dissolved in ethanol, followed by gentle rinsing with 70% ethanol and distilled water. The samples were then dried and imaged using TEM. Positive signals were identified by the presence of 10 nm dark gold particles indicating antigen expression.

**Dual-luciferase reporter assay**

Dual-luciferase reporter assay was performed as described in our previous study^[2]^. Briefly, a *FASLG* promoter region containing the predicted TCF7L2 binding sites or the corresponding mutant sites was constructed to the pGL3-basic luciferase reporter vector (GeneChem, China), respectively. The TCF7L2 plasmid (p-TCF7L2) was co-transfected with the luciferase reporter vector in hRIFs with Lipo2000 (Invitrogen, USA) for 2 days, and the dual-luciferase activity (firefly/renilla luciferase) was assessed in the dual‐Luciferase Reporter assay system (Promega, USA). Similarly, we performed the dual-luciferase reporter assay of LEF1 and TCF7 on their putative binding sites in *FASLG* promoter region.

**Liquid Chromatography-Tandem Mass Spectrometry (LC-MS/MS)**

Proteomics screening was performed by LC-MS/MS^[6]^. Briefly, after digestion with trypsin, the resulting peptides were separated by Ultimate 3000 RSLCnano system (ThermoFisher, USA), and MS was accomplished using Q Exactive Plus hybrid quadrupole-Orbitrap mass spectrometer (ThermoFisher, USA). The peptide database was referred to UniProt Human, and data were analyzed by Proteome Discover 2.5 software (ThermoFisher, USA), with trypsin specificity set as ≤2 missed cleavages.

**Flow cytometry**

hRPSEC apoptosis was assessed using an Annexin V-FITC/PI Apoptosis Detection Kit (Bioss, China). Cells were harvested, washed twice with cold PBS, and a suspension of 1-5 × 10⁵ cells was prepared in 100 µL of 1× binding buffer. The suspension was incubated with 5 µL of Annexin V-FITC and 5 µL of PI for 15 minutes in the dark at room temperature. Immediately, 400 µL of binding buffer was added prior to analysis. The stained samples were analyzed within one hour on a NL-3000 Full Spectrum Flow Cytometer (Cytek, USA). Using a 488 nm laser for excitation, the fluorescence emission of FITC (Annexin V) was detected through a 525 nm bandpass filter (plotted as the x-axis), and the fluorescence of PI was detected through a red fluorescence filter. The cell population was thereby divided into three main groups for analysis: viable cells (FITC⁻/PI⁻), early apoptotic cells (FITC⁺/PI⁻), and late apoptotic/necrotic cells (FITC⁺/PI⁺).

**Von Kossa and H&E staining**

Calcium deposits in the renal papillae were stained using the Von Kossa Kit (Solaribo, China) according to the manufacturer’s protocol, followed by counterstaining with hematoxylin and eosin (H&E). For histological analysis, tissue sections were stained with H&E using commercially available kits (Solaribo, China), following the manufacturer's instructions.

**Mating strategy of mice**

To activate the inducible Cre-ERT system, mice were injected intraperitoneally with tamoxifen (75 mg/kg in corn oil (Sigma, USA) every other day for 5 times, beginning at weaning (3 weeks of age). To introduce the *Col1a2-CreERT*^tg/+^ and *Faslg*^flox/flox^ alleles, *Col1a2-CreERT*^tg/+^ males were first crossed with *Faslg*^flox/flox^ females to obtain *Col1a2-CreERT*^tg/+^; *Faslg*^+/flox^ offspring, which were then bred with *Faslg*^flox/flox^ mice to produce *Col1a2-CreERT*^tg/+^; *Faslg*^flox/flox^ mice. Subsequently, *Umod*^–/–^ mice were crossed with *Col1a2-CreERT*^tg/+^; *Faslg*^flox/flox^ mice to obtain *Umod*^+/–^; *Col1a2-CreERT*^tg/+^; *Faslg*^+/flox^ offspring, followed by multiple crosses to obtain *Umod*^–/–^; *Faslg*^flox/flox^ mice and *Umod*^–/–^; *Col1a2-CreERT*^tg/+^; *Faslg*^flox/flox^ mice. Similarly, *Npt2a*^–/–^; *Col1a2-CreERT*^tg/+^; *Faslg*^flox/flox^ mice and *Npt2a*^–/–^; *Faslg*^flox/flox^ were obtained by the breeding strategy applied in the *Npt2a*^–/–^ background. To prevent undesired germline recombination of the floxed Faslg allele, the *Col1a2-CreERT*^tg/+^ transgene was always transmitted through the male germline.

**Virtual screening**

The THY1 protein structure was processed using the Protein Preparation Wizard module, including hydrogen addition followed by energy minimization (OPLS2005 force field, RMSD convergence threshold of 0.3 Å). A receptor grid file was generated by Schrödinger software with box size set to 20Å × 20Å × 20Å. Thereafter, virtual screening was accomplished by Schrödinger's docking workflow module. 107,015 compounds were firstly screened by High Throughput Virtual Screening (HTVS) Module in the Glide program. The compounds with top-ranked 20% scores of HY-L001P Library and top-ranked 15% scores of HY-L901P Library were selected for the second standard precision (SP) module. Next, top 20%-ranked compounds of HY-L001P Library and top 15%-ranked compounds of HY-L901P Library were included for extra precision (XP) module. Subsequently, molecular mechanics with generalized born surface area (MMGBSA) were calculated for top 20%-ranked compounds of HY-L001P Library and top 15%-ranked compounds of HY-L901P Library screened by XP modules, followed by the calculation of ligand strain energy for these compounds with MMGBSA < -30kcal/mol. Next, the compounds with ligand strain energy < 5kcal/mol were selected for protein ligand interaction fingerprints (PLIF) and structure diversity analysis.

**Administration of Benarthin in mice**

Considering that our in vitro experiment showed a significantly inhibitory effect on anoikis of hRPSECs with 20μM, and a previous study indicated a low toxicity after intravenous injection at 100 mg/kg of Benarthin to mice^[7]^, 100 mg/kg of Benarthin was administered to mice in the current study. Given that Umod^-/–^ mice and Npt2a^-/–^ mice exhibited marked renal interstitial calcium deposition within renal papillae at 6 months of age, and loss of renal papillary epithelium was observed at 9 months of age, the administration of Benarthin began at 6 months of age and continued for 3 months, and mice were sacrificed at 9 months of age. Because of long-term administration, Benarthin (MCE HY-117738, USA) was administered in drinking water ad libitum at 100 mg/kg/day, as water consumption was measured for 14 days prior to administration. The Benarthin solution was prepared weekly by dissolving Benarthin at the respective doses to autoclaved water containing 2% solubilizing agent (2-Hydroxypropyl-β-cyclodextrin; HP-β-CD; MCE HY-101103, USA), and HPLC was utilized to confirm the stability of Benarthin solution.

**Supplementary Results**

**Virtual screening for compounds interacting with THY1**

A receptor grid file was generated centered on the top-ranked binding site (Site1) predicted by Schrödinger software (**Figure S10B**), with surrounding residues VAL22, HIS111, and ILE115 defining the binding pocket (**Figure S10C**). Given HY-L001P Library collected 27,015 bioactive small-molecule compounds, including natural products, novel compounds, approved drugs, and clinical-stage compounds, and HY-L901P collected 80,000 drug-like compounds, the above two libraries were selected to virtual screening for compounds interacting with THY1, as performed in previous studies^[8,9]^. Virtual screening identified 1,770 compounds (**Table S18, S19**) with rich structural diversity (**Figure S10D, E**), which potentially interacted with THY1. Meanwhile, we found a negative correlation of molecular weight with binding free energy (ΔG) (**Figure S10F-I**), and protein ligand interaction fingerprints (PLIF) analysis suggested that the key residues mediating binding in THY1 protein included VAL222, HIS111, and ILE115, echoing the surrounding residues that define the binding pocket (**Figure S10J, K**).

**Biosafety of Benarthin**

To evaluate the biosafety of Benarthin *in vivo*, 6-month-old wt mice received Benarthin in drinking water ad libitum at 100 mg/kg/day for 3 months, and H&E staining was performed to assess histopathological change of five major mouse organs, including lung, heart, liver, spleen, and kidney (**Figure S11A-C**). The result showed intact tissue structures in all organs, with no signs of tissue damage or inflammatory cell infiltration, indicating the acceptable biosafety of Benarthin.

**Supplementary Figures**

**
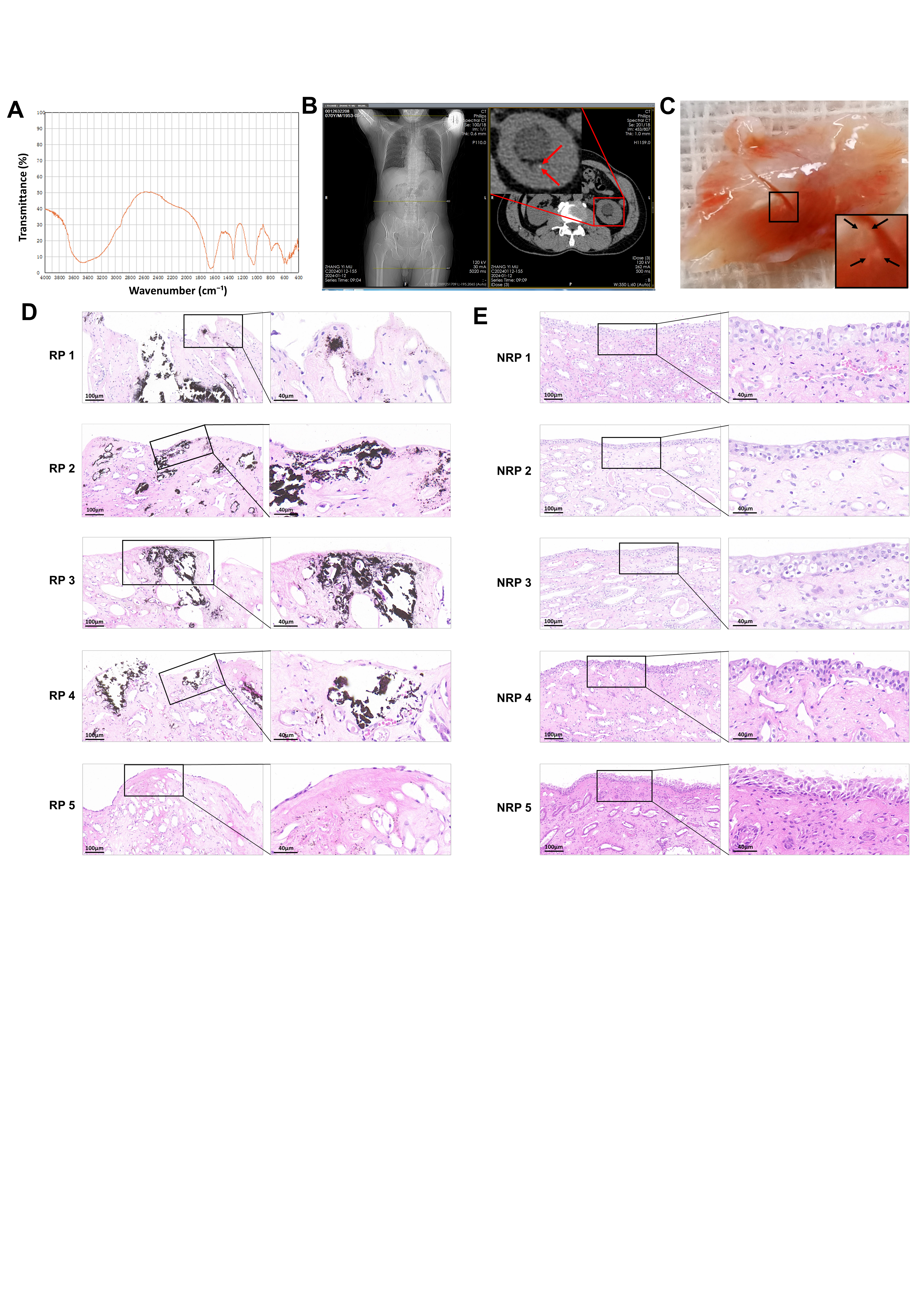
**

**Figure S1 A)** Fourier-transform infrared microspectroscopy spectrum of a kidney stone specimen from a patient. **B)** A non-contrast CT scan showed a renal papilla with RP (red arrows) in a patient with kidney stones. **C)** An intact renal papilla with RP (black arrows) from a nephrectomy specimen of a renal cancer patient. **D)** Von-Kossa staining showing RP with calcium deposits beneath hRPSECs (n = 5). **E)** Von-Kossa staining of NRP (n = 5).


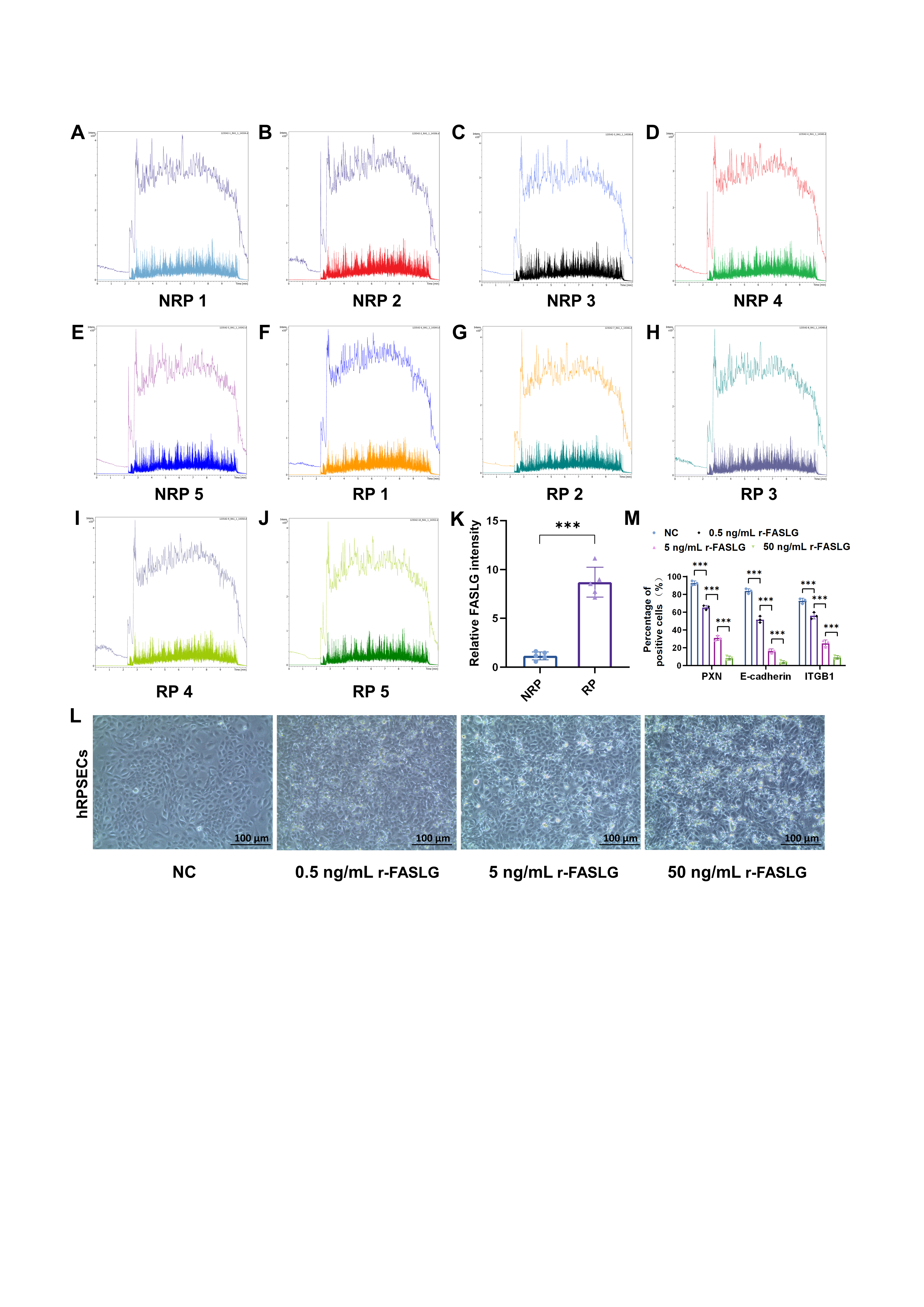


**Figure S2 A-J)** Representative base peak chromatograms from the LC-MS/MS analysis. In each panel, the upper trace represents the MS1 base peak intensity over the chromatographic gradient, reflecting the overall peptide elution profile. The lower trace shows the corresponding MS2 scan events, indicating the points at which peptides were selected for fragmentation and identification. **K)** Quantification of FASLG immunofluorescence intensity in NRP and RP tissues (n = 5 per group). **L)** Light microscopy showing the morphology of hRPSECs treated with r-FASLG at concentrations of 0, 0.5, 5, and 50 ng/mL for 3 days. **M)** hRPSECs were treated with r-FASLG at concentrations of 0, 0.5, 5, and 50 ng/mL for 3 days, followed by quantitative analysis of adhesion molecule expression (PXN, E-cadherin, and ITGB1).

**
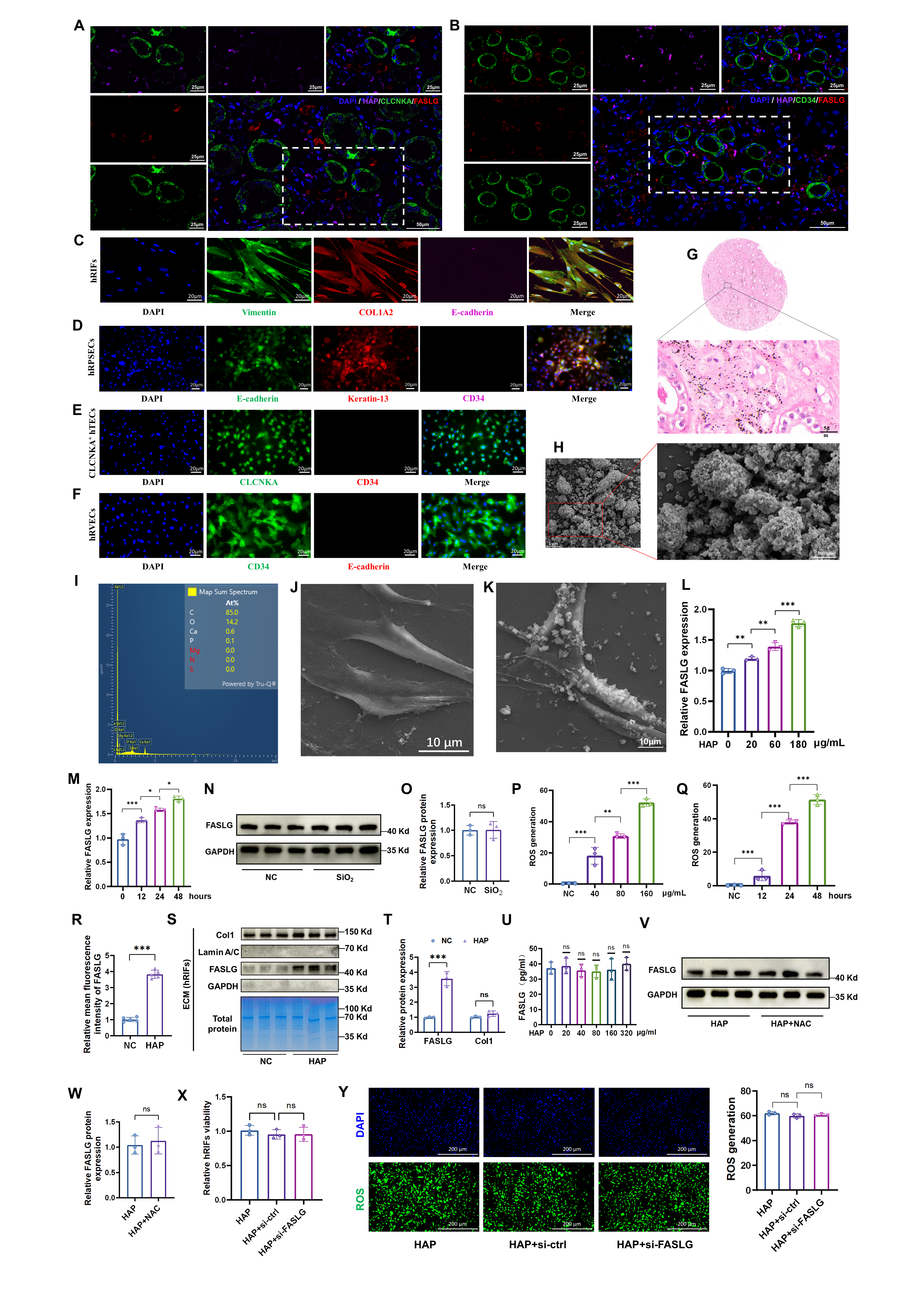
**

**Figure S3 A)** IF detection of HAP crystals distribution (OSTEO680, purple) in RP tissue, along with the expression of FASLG (red) in CLCNKA-labeled hTECs (green). **B)** IF detection of HAP crystals distribution (OSTEO680, purple) in RP tissue, along with the expression of FASLG (red) in CD34-labeled hRVECs (green). **C)** hRIFs were isolated from NRP tissues and identified by IF with Vimentin (green) and COL1A2 (red). **D)** hRPSECs were isolated from NRP tissues and identified by IF with E- cadherin (green) and Keratin-13 (red). **E)** CLCNKA^+^ hTECs were isolated from NRP tissues and identified by IF with CLCNKA (green). **F)** hRVECs were isolated from NRP tissues and identified by IF with CD34 (green). **G)** The size of HAP crystals observed in RP tissues. **H, I)** SEM and X-ray EDS determined the elemental component of HAP crystals. **J, K)** SEM observation of hRIFs and hRIFs co-cultured with HAP crystals. **L)** Quantification of FASLG protein expression in hRIFs treated with HAP crystals at increasing concentrations (0, 40, 80, and 160 μg/mL) for 3 days, n = 3. **M)** Quantification of FASLG protein expression in hRIFs treated with 80 μg/mL HAP crystals for 0, 12, 24, and 48 hours, n = 3. **N, O)** WB analysis of FASLG protein levels in hRIFs following treatment with SiO_2_ crystals for 48 hours, n=3. **P)** Quantification of intracellular ROS levels in hRIFs treated with HAP crystals at concentrations of 0, 40, 80, and 160 μg/mL for 3 days, n = 3. **Q)** Quantification of intracellular ROS levels in hRIFs treated with 80 μg/mL HAP crystals for 0, 12, 24, and 48 hours, n = 3. **R)** Quantification of FASLG immunofluorescence intensity in RP tissue sections. **S, T)** Representative WB analysis of proteins extracted from the decellularized ECM of hRIFs following HAP treatment. Collagen Type I (Col1) served as an ECM-specific loading control. The absence of GAPDH (cytosolic marker) and Lamin A/C (nuclear marker) confirmed the purity of the extracted ECM. Total protein loading was visualized by Coomassie Brilliant Blue staining, n=3. **U)** ELISA analysis of FASLG protein levels in the culture supernatants of hRPSECs treated with increasing concentrations of HAP crystals (0, 20, 40, 80, 160, 320 μg/mL). **V, W)** WB analysis of FASLG expression in hRIFs treated with HAP crystals (80 μg/mL) in the presence or absence of the ROS scavenger N-acetylcysteine (NAC) for 48 hours, n=3. **X)** CCK-8 assay assessing the relative viability of hRIFs treated with HAP crystals (80 μg/mL) alone, or transfected with negative control siRNA (si-ctrl) or FASLG-targeting siRNA (si-FASLG) under HAP stimulation for 3 days, n=3. **Y)** Representative fluorescence images of intracellular ROS levels (green) in hRIFs treated with HAP crystals (80 μg/mL) alone, or transfected with si-ctrl or si-FASLG under HAP (80 μg/mL) stimulation for 3 days, n=3.

**
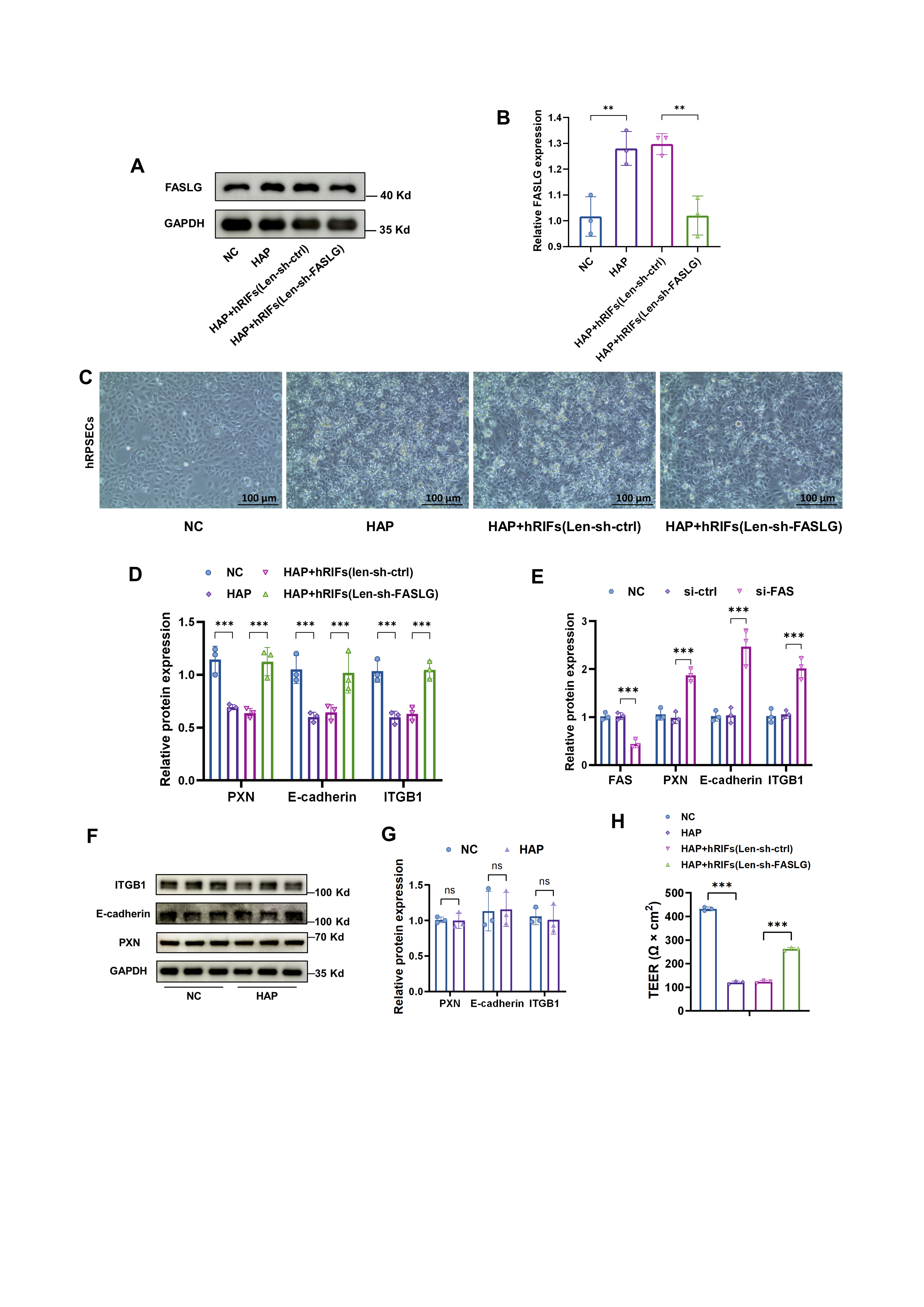
**

**Figure S4 A, B)** WB analysis of FASLG expression in hRIFs, HAP-treated hRIFs, HAP+hRIFs (Len-sh-ctrl), and HAP+hRIFs (Len-sh-FASLG); n = 3. **C)** Light microscopy showing the morphology of hRPSECs co-cultured for 3 days with hRIFs, HAP-treated hRIFs, HAP+hRIFs (Len-sh-ctrl), and HAP+hRIFs (Len-sh-FASLG). **D)** Quantification of adhesion molecule expression (PXN, E-cadherin, and ITGB1) in hRPSECs after co-culture for 3 days with hRIFs, HAP-treated hRIFs, HAP+hRIFs (Len-sh-ctrl), or HAP+hRIFs (Len-sh-FASLG); n = 3. **E)** Quantification of adhesion molecule expression (PXN, E-cadherin, and ITGB1) in hRPSECs transfected with si-FAS and co-cultured with HAP-treated hRIFs for 3 days, n = 3. **F, G)** WB analysis of adhesion-related proteins (ITGB1, E-cadherin, and PXN) in hRPSECs following direct stimulation with HAP crystals in the absence of hRIFs, n=3. **H)** Transepithelial Electrical Resistance (TEER) assay was performed to quantitatively assess the barrier function of hRPSECs in a Transwell co-culture system. hRPSECs were co-cultured with normal hRIFs (NC), HAP-treated hRIFs, or HAP-treated hRIFs transfected with control shRNA (Len-sh-ctrl) or FASLG-targeting shRNA (Len-sh-FASLG).

**
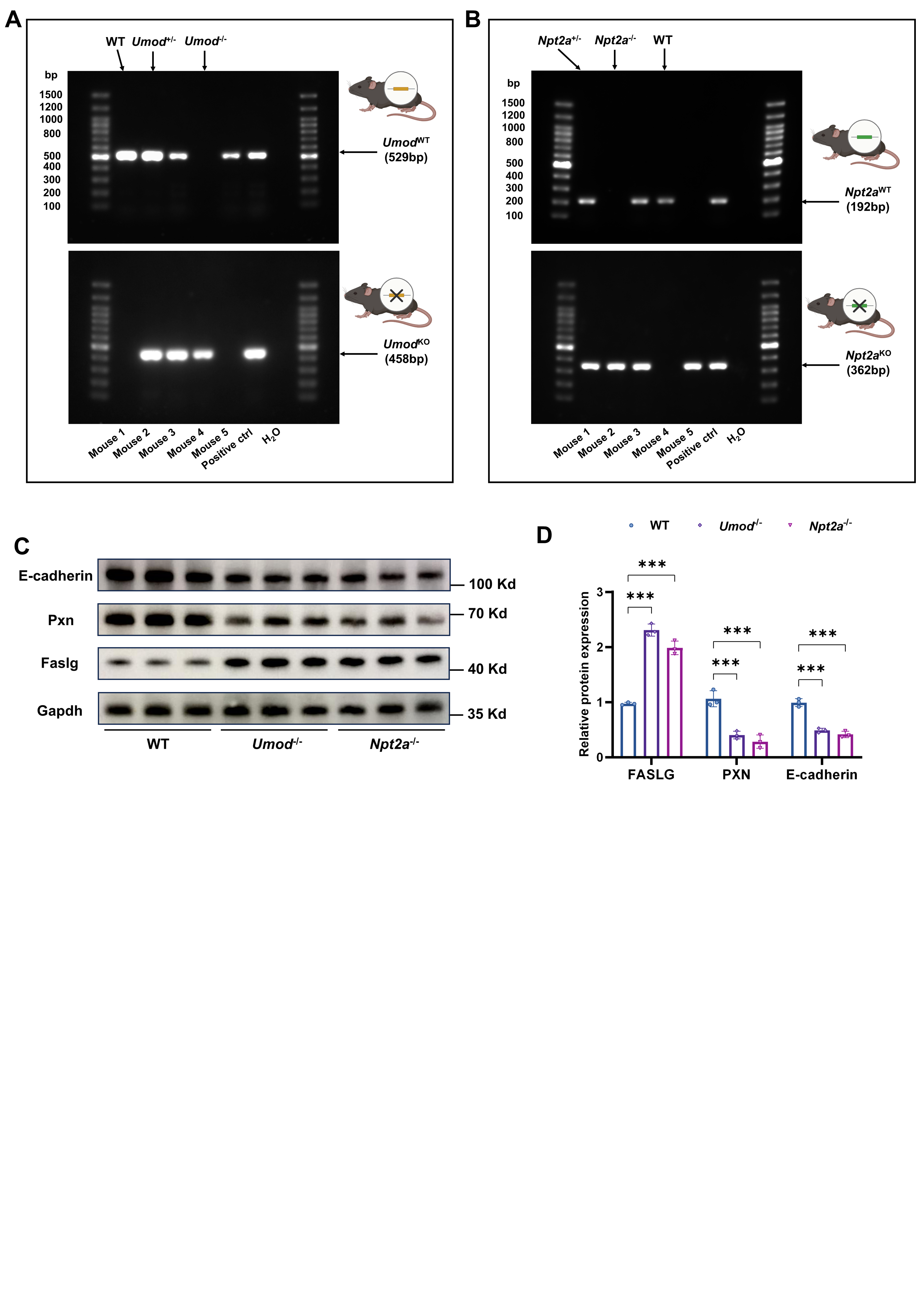
**

**Figure S5 A, B)** Genotyping of offspring from *Umod*^+/–^ intercross or *Npt2a*^+/–^ intercross. PCR products were resolved by agarose gel electrophoresis. Each lane represents an individual mouse. Positive controls and a water (H₂O) blank were included for reference. **C, D)** WB analysis of FASLG and adhesion molecules (PXN and E-cadherin) in renal papillary sections from WT, *Umod*^⁻/⁻^, and *Npt2a*^⁻/⁻^ mice (n = 3 per group).

**
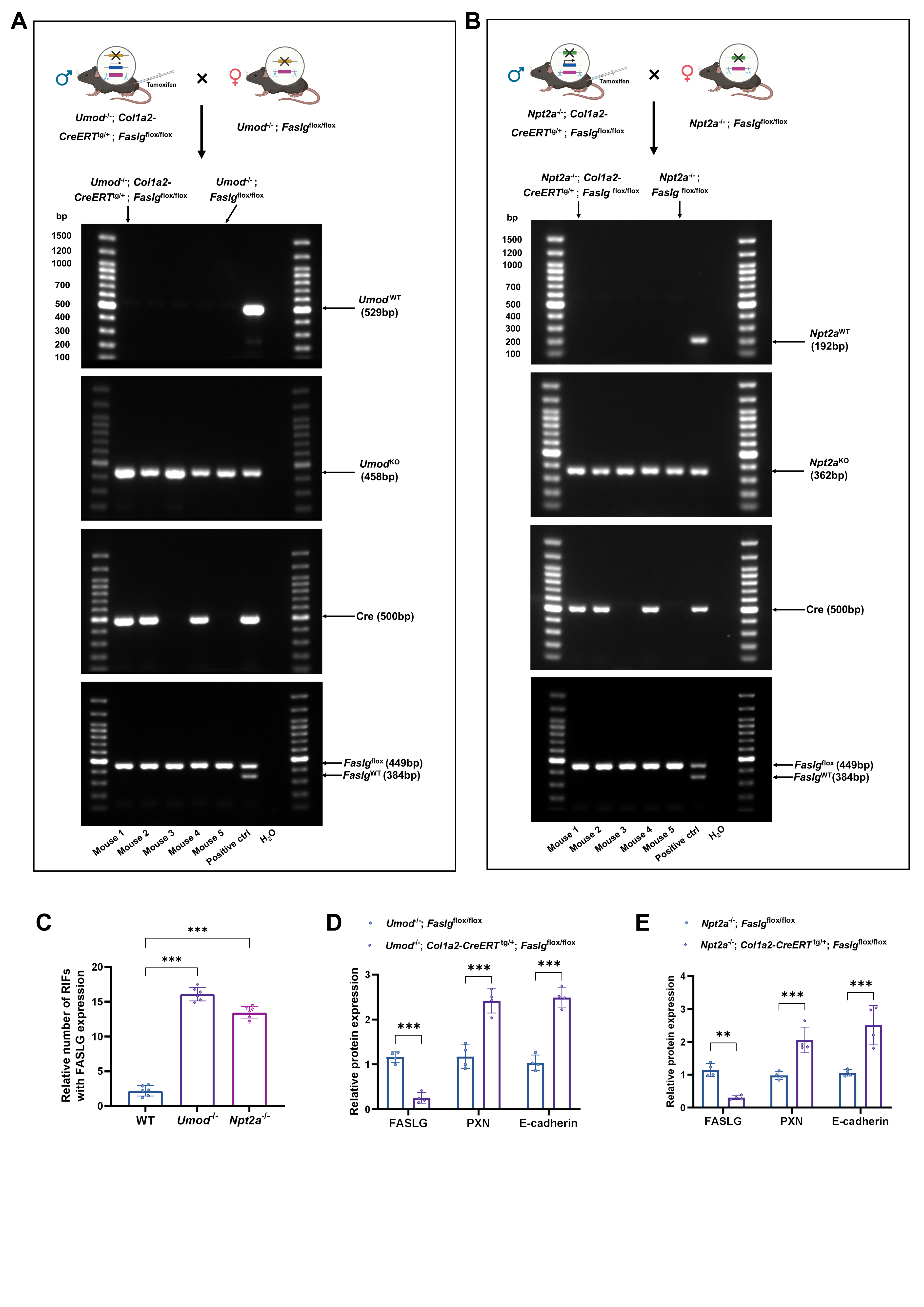
**

**Figure S6 A)** Offspring from the mating of a *Umod*^⁻/⁻^; *Col1a2-CreERT*^tg/+^; *Faslg*^flox/flox^ male with a *Umod*^⁻/⁻^; *Faslg*^flox/flox^ female were genotyped. **B)** Offspring from the mating of a *Npt2a*^⁻/⁻^; *Col1a2-CreERT*^tg/+^; *Faslg*^flox/flox^ male with a *Npt2a*^⁻/⁻^; *Faslg*^flox/flox^ female were genotyped. PCR products were resolved by agarose gel electrophoresis. Each lane represents an individual mouse. Positive controls and a water (H₂O) blank were included for reference. **C)** Relative number of hRIFs with FASLG expression in WT, *Umod*^⁻/⁻^, and *Npt2a*^⁻/⁻^ mice (n = 6 per group). **D, E)** Quantification of FASLG and adhesion molecule (PXN and E-cadherin) expression levels in renal papillary sections from *Faslg^Col1a2^* CKO mice with *Umod*^⁻/⁻^ or *Npt2a*^⁻/⁻^ background, and the corresponding controls without Cre (n = 4 per group).

**
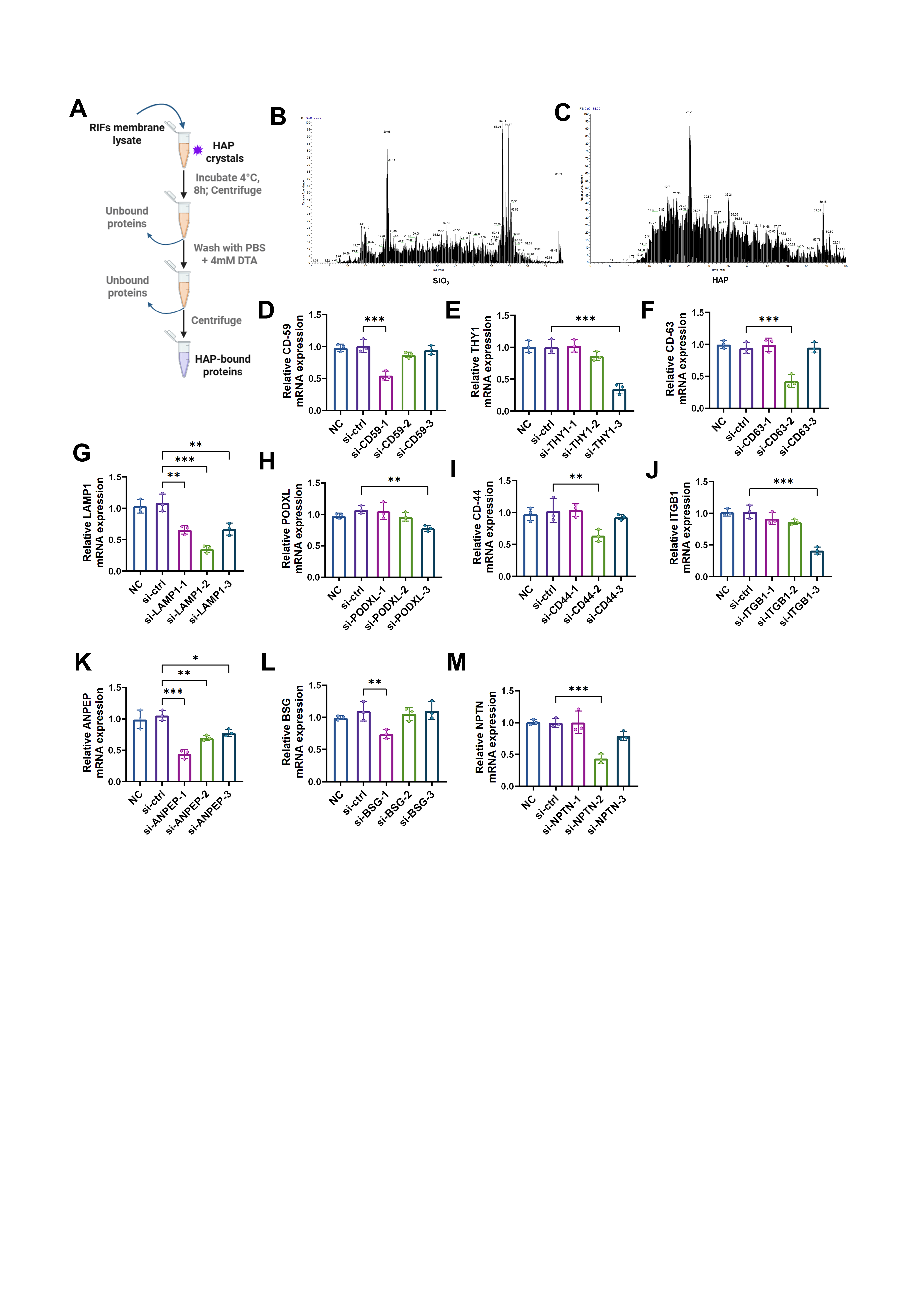
**

**Figure S7 A)** Schematic diagram illustrating the co-precipitation of HAP crystals and membrane proteins. **B, C)** Total Ion Chromatogram (TIC) of protein profiling from hRIFs membrane proteins bound to SiO2 and HAP crystals. **D-M)** qRT-PCR determined the efficiency of siRNA transfections to silence genes encoding those top 10 ranking membrane proteins. The siRNA with the highest efficiency was selected for the following experiments, n=3.

**
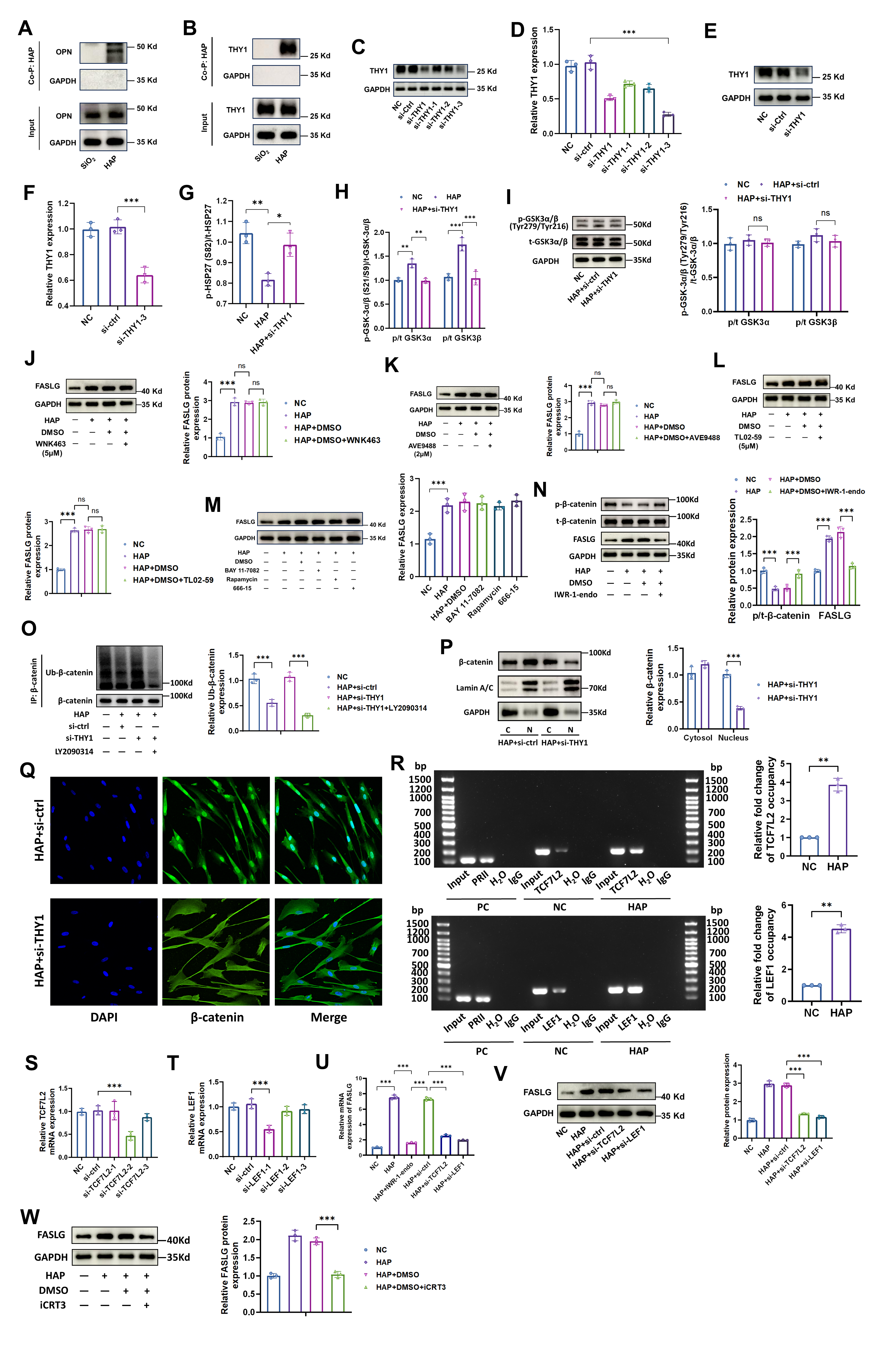
**

**Figure S8 A, B)** hRIFs were treated with SiO_2_ or HAP, followed by pull-down using HAP and WB detection of osteopontin (OPN) or THY1. **C, D)** WB analysis of THY1 knockdown in hRIFs transfected with three different siRNA sequences, n = 3. **E, F)** WB validation of the most effective THY1 knockdown siRNAs in hRIFs, n = 3. **G, H)** Quantification of p-GSK3α/β (Ser21/Ser9) and p-HSP27 (Ser82) expression levels, normalized to total GSK3α/β and HSP27 respectively, in NC, HAP, and HAP+si-THY1 groups, n = 3. **I)** WB analysis of p-GSK3α/β at activating tyrosine sites (Tyr279/Tyr216) and total GSK3α/β in hRIFs transfected with si-ctrl or si-THY1 followed by HAP treatment (80μg/mL), n=3. **J-L)** hRIFs were treated with HAP crystals in the presence or absence of specific modulators for candidate pathways identified in the phospho-kinase array: the WNK1 inhibitor WNK463 (5μM), the eNOS activator AVE9488 (2μM), or the Lyn inhibitor TL02-59 (5μM), followed by WB analysis of FASLG expression, n = 3. **M)** WB analysis of FASLG in hRIFs treated with HAP, selective inhibitors of NF-κB (BAY 11-7082; 5μM), mTOR (Rapamycin; 50μM), and CREB (666-15; 0.5μM), n = 3. **N)** Relative expression of FASLG in hRIFs induced by HAP crystals (80μg/mL) in the presence or absence of the specific β-catenin inhibitor IWR-1-endo (5μM), n=3. **O)** hRIFs were treated with HAP crystals and transfected with si-ctrl or si-THY1, with or without the GSK3α/β inhibitor LY2090314. Lysates were immunoprecipitated with an anti-β-catenin antibody and immunoblotted with an anti-ubiquitin antibody, n=3. **P)** WB analysis of β-catenin distribution in the cytoplasmic (C) and nuclear (N) fractions of hRIFs treated with HAP and transfected with si-ctrl or si-THY1. GAPDH and Lamin A/C were used as loading controls for the cytoplasmic and nuclear fractions, respectively, n=3. **Q)** Representative IF images showing the subcellular localization of β-catenin (green) in hRIFs treated with HAP and transfected with si-ctrl or si-THY1. **R)** Analysis of TCF7L2 and LEF1 occupancy at the FASLG promoter in hRIFs treated with or without HAP crystals. Representative agarose gel electrophoresis images of ChIP-qPCR products validating the physical binding. ChIP-qPCR quantitative analysis showing the relative fold enrichment of TCF7L2 and LEF1 in the HAP-treated group compared to the NC group. **S, T)** Validation of siRNA knockdown efficiency for TCF7L2 and LEF1 in hRIFs determined by q-PCR, n=3. **U)** FASLG mRNA expression was determined in hRIFs treated with HAP or in combination with the β-catenin inhibitor IWR-1-endo (5μM), si-ctrl, si-TCF7L2, or si-LEF1, n = 3. **V)** WB analysis of FASLG protein expression in HAP-treated hRIFs following transfection with si-ctrl, si-TCF7L2, or si-LEF1, n=3. **W)** WB analysis of FASLG protein expression in hRIFs induced by HAP crystals (80μg/mL) in the presence or absence of iCRT3 (50μM), n=3.

**
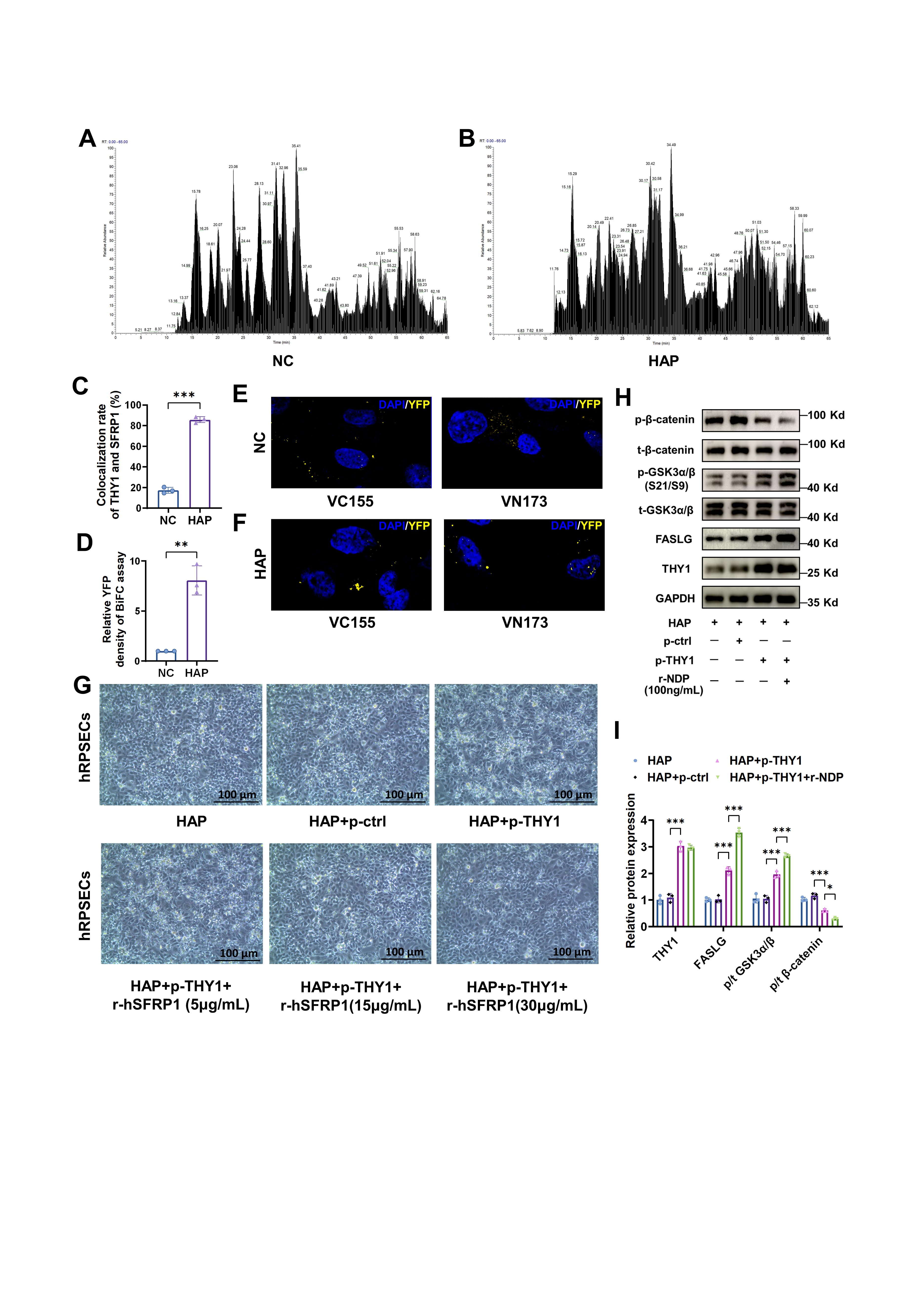
**

**Figure S9 A, B)** MS-based TICs of protein extracts from normal and HAP-treated hRIFs. **C)** The colocalization rate (%) of THY1 (green) and SFRP1 (red) was quantified based on confocal microscopy images of hRIFs treated with or without HAP crystals. **D)** The relative mean fluorescence intensity of the YFP signal was quantified to assess the physical interaction between THY1 and SFRP1 in hRIFs treated with or without HAP crystals. **E, F)** Representative confocal images of negative controls in hRIFs. hRIFs were co-transfected with either THY1-VC155 and an empty VN173 vector, or SFRP1-VN173 and an empty VC155 vector, and YFP signal was captured by a confocal microscopy. **G)** hRIFs transfected with p-ctrl or p-THY1 plasmids were treated with HAP crystals and r-hSFRP1, then co-cultured with hRPSECs for 3 days, and light microscopy showing the morphology of hRPSECs. **H, I)** hRIFs transfected with p-ctrl or p-THY1 plasmids were treated with HAP crystals and recombinant NDP protein (r-NDP) at 100 ng/ml for 3 days, and protein expressions were determined by WB; n = 3.

**
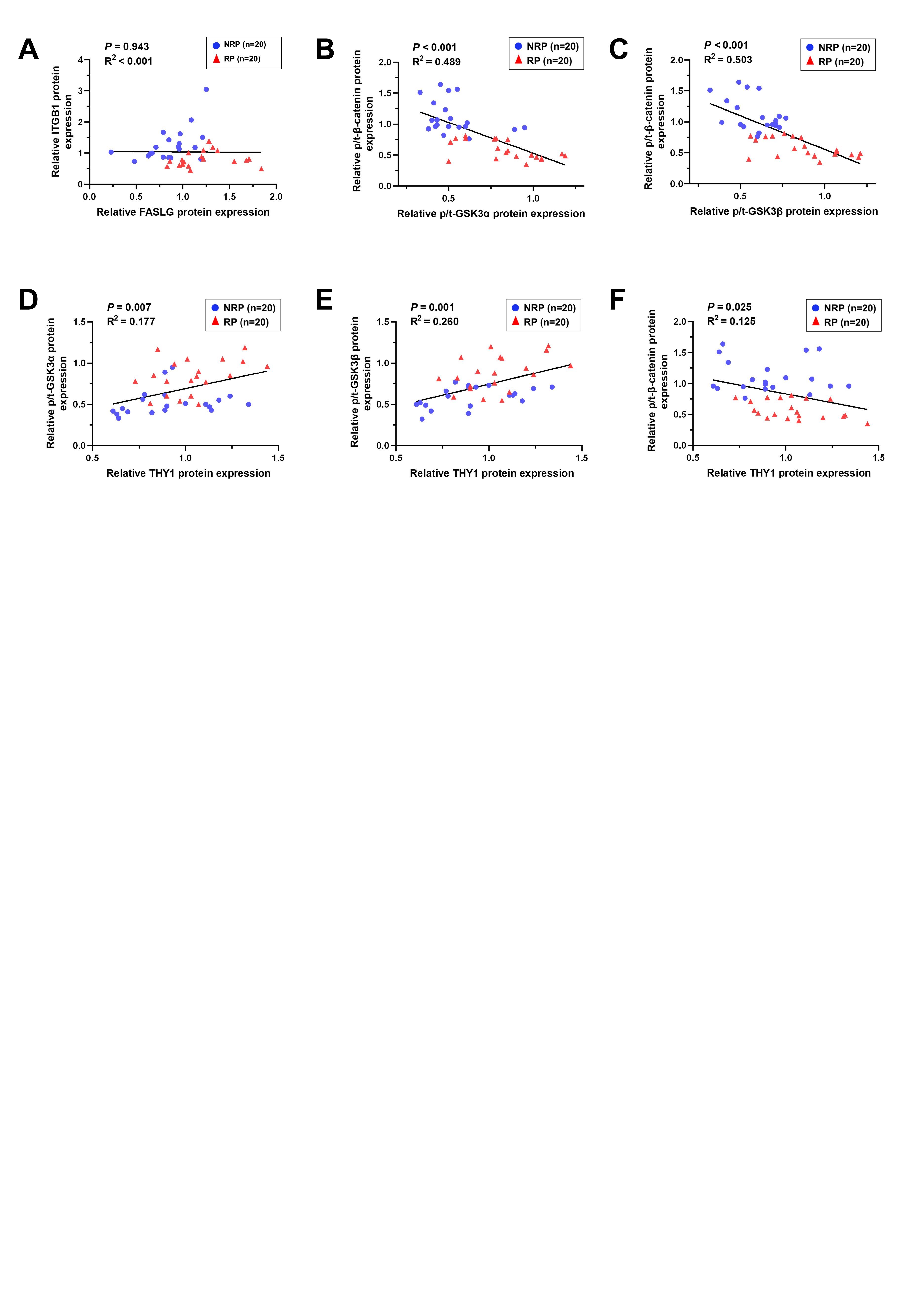
**

**Figure S10 A)** Correlation analysis between FASLG and ITGB1 levels. **B, C)** Correlation analysis between (B) p/t-GSK3α, (C) p/t-GSK3β and p/t-β-catenin levels. **D-F)** Correlation analysis between (D) p/t GSK3α, (E) p/t GSK3β, (F) p/t β-catenin and THY1.

**
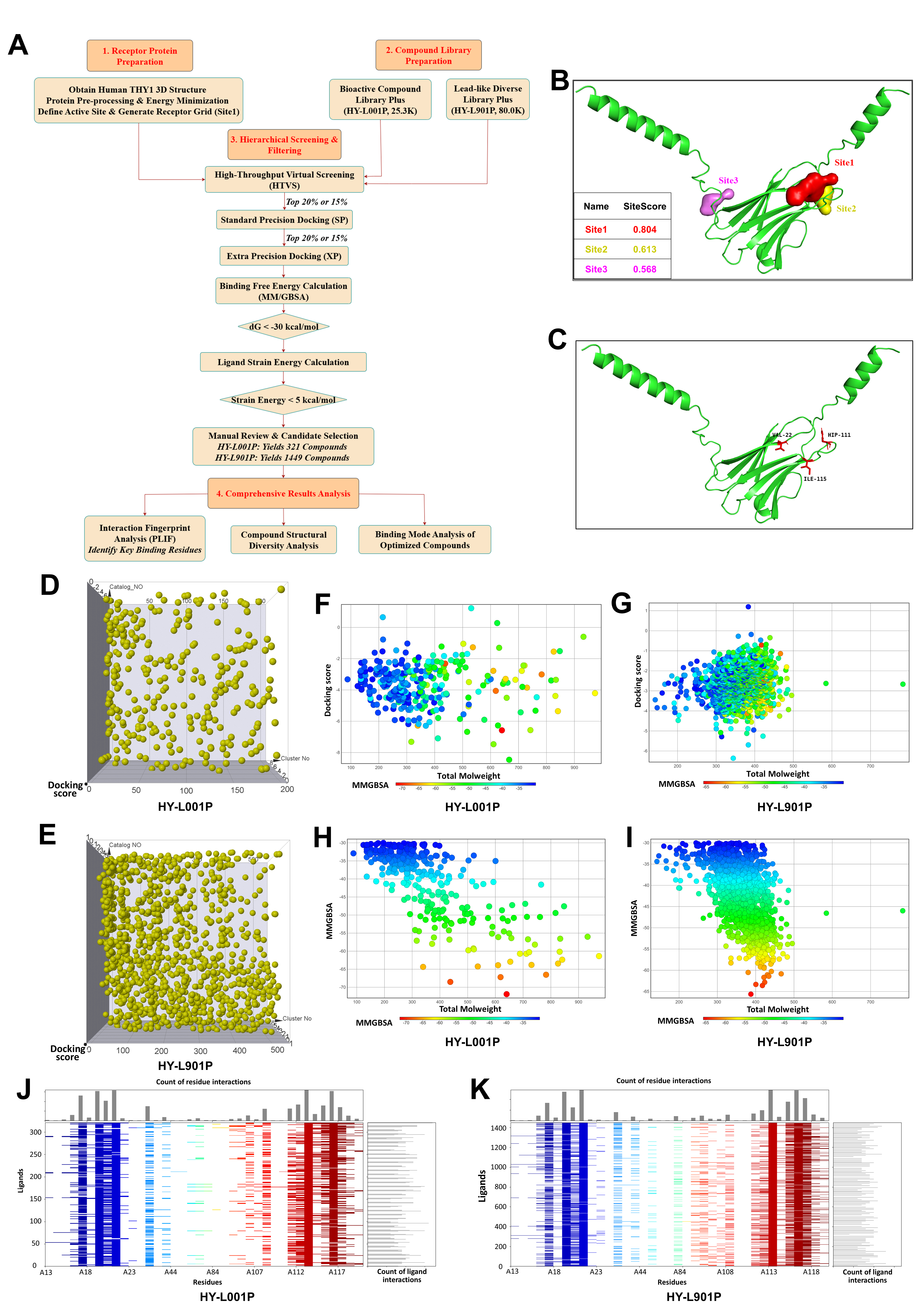
**

**Figure S11** Virtual screening strategy and analysis of hit compounds for THY1. **A)** Workflow of the virtual screening process for identifying THY1 binders. **B)** The top three potential binding sites predicted on the human THY1 protein structure and their corresponding SiteScores. **C)** Visualization of the Site1 binding pocket with key residues (VAL22, HIS111, ILE115) used for docking grid generation. **D, E)** Structural diversity analysis of hit compounds from the (D) HY-L001P bioactive compound library and (E) HY-L901P drug-like library. **F, G)** Docking score versus molecular weight for hits from the HY-L001P and HY-L901P libraries, respectively. **H, I)** MM/GBSA binding free energy (ΔG) versus molecular weight for hits from the same two libraries, respectively. **J, K)** Protein-Ligand Interaction Fingerprint (PLIF) summary for hit compounds from the (J) HY-L001P and (K) HY-L901P libraries. The top histograms show the interaction frequency for each THY1 residue.


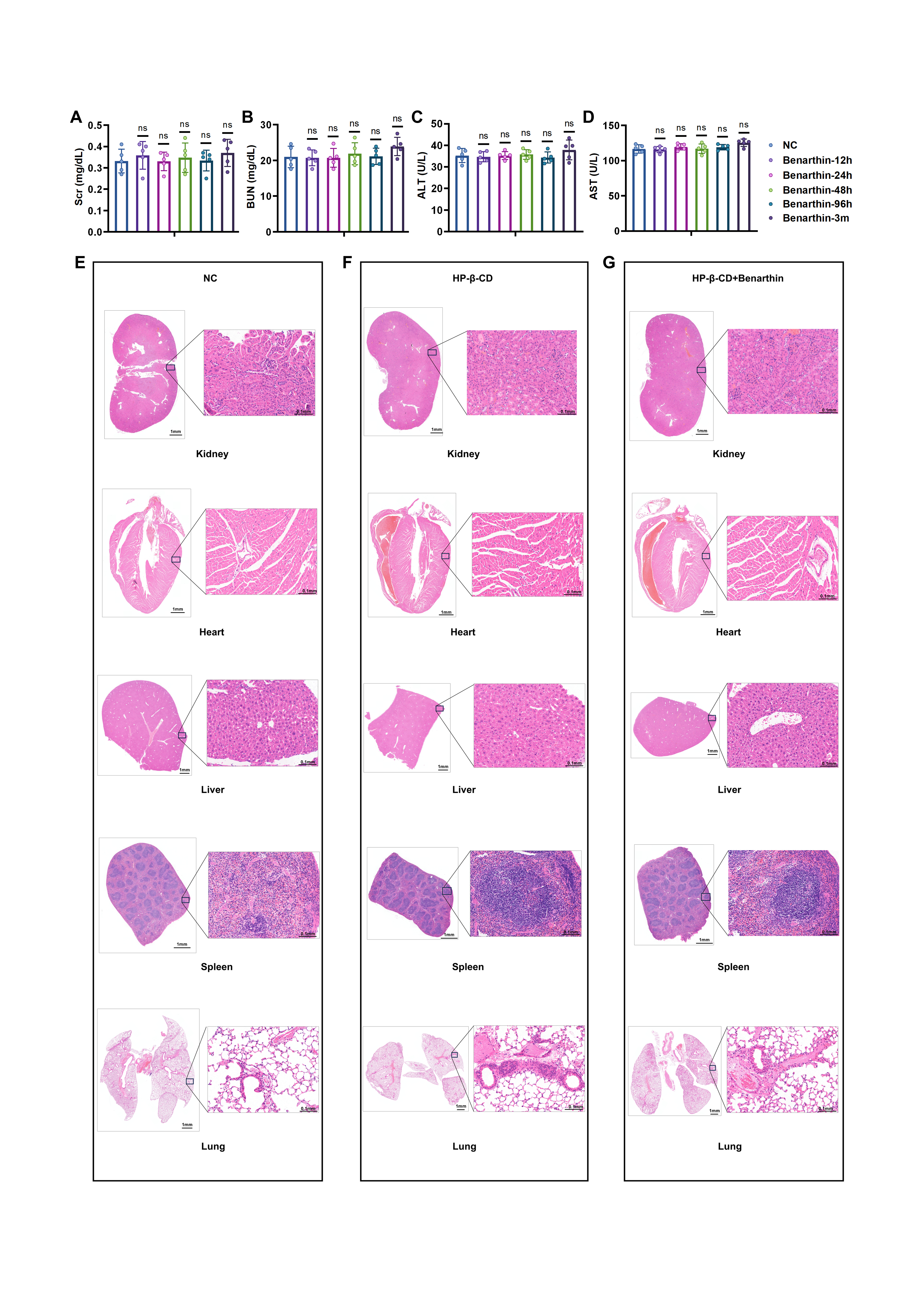


**Figure S12 A-D)** Comprehensive time-course analysis of serum biochemical markers in mice administered Benarthin. Serum samples were collected at acute time points (12, 24, 48, and 96 hours) and a chronic time point (3 months) to assess renal function (A: Serum Creatinine, Scr; B: Blood Urea Nitrogen, BUN) and hepatic function (C: Alanine Aminotransferase, ALT; D: Aspartate Aminotransferase, AST). **E-G)** Histological analysis of major organs from mice treated with Benarthin. Representative hematoxylin and eosin (H&E) stained sections of kidney, heart, liver, spleen, and lung from mice treated for 3 months with (A) normal control (NC), (B) HP-β-CD, or (C) HP-β-CD+Benarthin (100 mg/kg/day).

**Supplementary Tables**

**Table S1** List of proteins identified and quantified by mass spectrometry in RP and NRP tissues (Excel file).

**Table S2** List of the anoikis-related genes used for Gene Set Enrichment Analysis (Excel file).

**Table S3** List of proteins identified by mass spectrometry from hRIFs membrane lysates bound to HAP crystals (Excel file).

**Table S4** List of proteins identified by mass spectrometry from hRIFs membrane lysates bound to SiO₂ crystals (Excel file).

**Table S6** Predicted binding sites for TCF7L2, LEF1 and TCF7 in the *FASLG* Promoter.

| **TFs** | **Motif** | **Strand of *FASLG*** | **Score** | **Matched sequence** | **Motif Site** | **Motif web** |
| --- | --- | --- | --- | --- | --- | --- |
| TCF7L2 | MA0523.2 | + | 9.4 | ACATGAAAA | TSS -1756bp | https://jaspar.elixir.no/matrix/MA0523.2/ |
| LEF1 | MA0768.1 | + | 9.5 | AAACATGAAAAAAAT | TSS -1758bp | https://jaspar.elixir.no/matrix/MA0768.1/ |
| TCF7 | MA0769.2 | + | 9.8 | TTCTTTGAGAA | TSS -108bp | https://jaspar.elixir.no/matrix/MA0769.2/ |

**Table S7** List of THY1-interacting membrane proteins identified by IP-MS in control hRIFs. (Excel file).

**Table S8** List of THY1-interacting membrane proteins identified by IP-MS in HAP-treated hRIFs. (Excel file).

**Table S9** Baseline clinical characteristics of 263 CaOx stone patients undergoing PCNL, stratified by stone recurrence status during 5-year follow-up.

| **Characteristics** | **Total (n=263)** | **Kidney stone recurrence** | |
| --- | --- | --- | --- |
|  |  | **No (n=197)** | **Yes (n=66)** |
| Sex (%) |  |  |  |
| Female | 100 (38.02%) | 80 (40.61%) | 20 (30.30%) |
| Male | 163 (61.98%) | 117 (59.39%) | 46 (69.70%) |
| Age, years, mean (SD) | 50.3 (9.4) | 51.4 (9.5) | 47.1 (8.0) |
| BMI, kg/m^2^, mean (SD) | 23.4 (3.0) | 23.1 (3.0) | 24.3 (3.0) |
| T2DM, n (%) |  |  |  |
| No | 233 (88.59%) | 175 (88.83%) | 58 (87.88%) |
| Yes | 30 (11.41%) | 22 (11.17%) | 8 (12.12%) |
| Hypertension, n (%) |  |  |  |
| No | 198 (75.29%) | 153 (77.66%) | 45 (68.18%) |
| Yes | 65 (24.71%) | 44 (22.34%) | 21 (31.82%) |
| Hyperlipemia, n (%) |  |  |  |
| No | 235 (89.35%) | 179 (90.86%) | 56 (84.85%) |
| Yes | 28 (10.65%) | 18 (9.14%) | 10 (15.15%) |
| History of previous stone surgery, n (%) |  |  |  |
| No | 191 (72.62%) | 148 (75.13%) | 43 (65.15%) |
| Yes | 72 (27.38%) | 49 (24.87%) | 23 (34.85%) |
| Serum creatinine, μmol/L, mean (SD) | 100.7 (62.2) | 98.0 (57.8) | 108.8 (73.9) |
| Serum calcium, mmol/L, mean (SD) | 2.29 (0.11) | 2.29 (0.11) | 2.29 (0.09) |
| Serum phosphate, mmol/L, mean (SD) | 1.17 (0.19) | 1.17 (0.20) | 1.17 (0.17) |
| Serum PTH, pg/ml, mean (SD) | 40.34 (9.75) | 40.17 (9.96) | 40.87 (9.11) |
| Urinary calcium-to-creatinine ratio, mean (SD) | 0.213 (0.078) | 0.208 (0.079) | 0.227 (0.077) |
| Urinary phosphate-to-creatinine ratio, mean (SD) | 0.212 (0.074) | 0.212 (0.070) | 0.212 (0.084) |
| Urinary oxalate-to-creatinine ratio, mean (SD) | 0.074 (0.040) | 0.075 (0.040) | 0.072 (0.039) |
| Urinary citrate-to-creatinine ratio, mean (SD) | 0.278 (0.116) | 0.278 (0.116) | 0.278 (0.115) |
| FASLG expression in hRIFs (%), mean (SD) | 11.49 (4.50) | 10.18 (3.97) | 15.39 (3.65) |

SD, standard deviation; BMI, body mass index; T2DM, type 2 diabetes mellitus

**Table S10** Multivariate logistic regression analysis of risk factors for recurrence in patients with CaOx stones.

| **Risk factors** | **OR (95% CI)** | **P value** |
| --- | --- | --- |
| Sex, (female/male) | 1.62 (0.74-3.52) | 0.227 |
| Age, years | 0.91 (0.87-0.95) | < 0.001 |
| BMI, kg/m^2^ | 1.15 (1.01-1.30) | 0.030 |
| Hypertension, (yes/no) | 1.75 (0.74-4.18) | 0.205 |
| Hyperlipidemia, (yes/no) | 2.30 (0.77-6.90) | 0.137 |
| History of previous stone surgery, (yes/no) | 2.65 (1.20-5.88) | 0.016 |
| Hypercalciuria, (yes/no) | 0.87 (0.36-2.08) | 0.752 |
| FASLG expression in hRIFs, % | 1.41 (1.27-1.56) | < 0.001 |

BMI, body mass index

**Table S11** Docking information for the top 15 compounds from the molecular docking virtual screening against THY1.

| **Cat. No.** | **Name** | **Molecular Weight** | **Docking Score** | **Ligand Efficiency (kcal/mol)** | **Hydrogen Bond Acceptors** | **Hydrogen Bond Donors** | **Rotatable Bonds** | **Binding Free Energy** | **Ligand Strain Energy** |
| --- | --- | --- | --- | --- | --- | --- | --- | --- | --- |
| HY-N1499 | Nystose | 666.590 | -8.462 | -7.905 | 21 | 14 | 13 | -50.79 | 3.440 |
| HY-N9898 | Sinocrassoside C1 | 610.531 | -7.470 | -0.390 | 16 | 10 | 6 | -56.11 | 1.120 |
| HY-12656 | SCH 51344 | 316.363 | -7.315 | 0.836 | 5 | 3 | 7 | -57.11 | 1.345 |
| HY-117738 | Benarthin | 411.418 | -7.095 | -3.553 | 5 | 5 | 11 | -43.77 | 3.679 |
| HY-132142 | 5-Propargylamino-dCTP | 516.194 | -6.678 | -3.901 | 10 | 2 | 9 | -52.14 | 3.745 |
| HY-N0031 | Plantamajoside | 640.601 | -6.580 | -0.612 | 16 | 10 | 12 | -71.92 | 2.074 |
| HY-147059 | Dieckol | 742.566 | -6.569 | 6.150 | 18 | 11 | 6 | -58.19 | 2.332 |
| HY-N4168 | 3-O-Caffeoylquinic acid methyl ester | 368.344 | -6.289 | -0.272 | 9 | 5 | 6 | -37.82 | 0.909 |
| HY-100673 | LM22A-4 | 339.351 | -6.282 | -2.164 | 6 | 6 | 9 | -64.37 | 4.227 |
| HY-125323 | 6-Hydroxykaempferol 3,6-diglucoside | 626.530 | -6.270 | -1.278 | 17 | 11 | 7 | -50.94 | 4.596 |
| HY-B0735A | Fenoldopam (mesylate) | 305.764 | -6.185 | 1.069 | 2 | 3 | 1 | -31.44 | 0.020 |
| HY-N1132A | D-(+)-Trehalose dihydrate | 342.303 | -6.177 | -4.260 | 11 | 8 | 4 | -37.96 | 0.208 |
| HY-106263B | Tyroserleutide (hydrochloride) | 381.432 | -6.052 | -1.670 | 4 | 5 | 10 | -39.06 | 4.312 |
| HY-101277 | Vadadustat | 305.700 | -5.952 | 0.228 | 1 | 2 | 4 | -37.29 | 2.176 |
| HY-QS08583750 |  | 344.313 | -6.364 | -5.761 | 11.0 | 9.0 | 8.0 | -38.38 | 2.965 |

**Table S12** The characteristics of included 52 patients undergoing nephrectomy due to renal cancers.

| **Characteristics** | **NRP group (n=26)** | **RP group (n=26)** | ***P* value** |
| --- | --- | --- | --- |
| Age, years, mean (SD) | 54.9 (7.2) | 51.7 (7.6) | 0.125 |
| Gender, female/male | 8/18 | 10/16 | 0.771 |
| BMI, kg/m^2^, mean (SD) | 23.7 (5.4) | 24.6 (5.1) | 0.539 |
| Comorbidities, n (%) |  |  |  |
| Hypertension | 6 (23.1%) | 8 (30.8%) | 0.755 |
| T2DM | 4 (15.4%) | 6 (23.1%) | 0.725 |
| Tumor size, cm, mean (SD) | 4.6±0.8 | 4.4±0.7 | 0.342 |

NRP, normal renal papillae; RP, renal papillae with Randall’s plaques; SD, standard deviation; BMI, body mass index; T2DM, type 2 diabetes mellitus

**Table S13** Primer sequences designed for CHIP-qPCR.

| **Target regions** | **Forward primer 5′–3′** | **Reverse primer 5′–3′** |
| --- | --- | --- |
| TCF7L2 binding sites in *FASLG* promoter (210bp) | GTGTGGTACAGGTGAGTGGG | TCCTCTGGCCTCACATACCA |
| LEF1 binding sites in *FASLG* promoter (210bp) | GTGTGGTACAGGTGAGTGGG | TCCTCTGGCCTCACATACCA |
| TCF7 binding sites in *FASLG* promoter (320bp) | GGGGTTTGCTCTGAGCTTCT | CACTGCTGTCCACCCAGTAG |
| RNA Polymerase II binding sites in *GAPDH* promoter (positive control; 110bp) | Involved in the kit | Involved in the kit |

**Table S14** Primer sequences designed for mouse genotyping.

| **Gene** | **Forward primer 5′–3′** | **Reverse primer 5′–3′** |
| --- | --- | --- |
| *Umod* (WT: 529bp) | GTGATGTTTCTGGTGGGACAAGG | ATTCAGAACACCGTCCTGCG |
| *Umod* (KO: 458bp) | GTGATGTTTCTGGTGGGACAAGG | CAGAGGGTGGAAATTGGGTGAAC |
| *Npt2a* (WT: 192 bp) | TCATGGAGACAACGGAAGCC | GTCCCGTTCCCAAGATGTGT |
| *Npt2a* (KO: 362 bp) | AAGGGCCCCTCAGCTACTAT | ATATCCAGCCCCCTCATCCA |
| *Faslg* (WT: 384bp; flox: 449bp) | CAGTCTTGCAACAACCAGCC | CCTTGCGGTCATGAGGTCTT |
| Col1a2-CreERT2 (500bp) | GATTGGTCTCGTCTGGCGCT | GGCTAGTGGGCGCATGTA |

**Table S15** Targeted sequences were designed for gene silencing or overexpression.

| **Targeted Gene** | **Reconstituted lentivirus or siRNA** | **Targeted sequences (5’-3’)** |
| --- | --- | --- |
| *FASLG* | Len-sh1-*FASLG* | GCAGTGTTCAATCTTACCAGT |
|  | Len-sh2-*FASLG* | ACTGGGCTGTACTTTGTATAT |
|  | Len-sh3-*FASLG* | GCCCAGAAGGCCTGGTCAAAG |
| *FAS* | si-*FAS*-1 | GACACUAAGUCAAGUUAAAGG |
|  | si-*FAS*-2 | CAAGAAUGACAAUGUCCAAGA |
|  | si-*FAS*-3 | AGCAGAACAGAAAGUUCAACU |
| *THY1* | si-*THY1*-1 | GAACCAACTTCACCAGCAA |
|  | si-*THY1*-2 | GCAGTTCACCCATCCAGTA |
|  | si-*THY1*-3 | ACATGAAGGTCCTCTACTT |
| *CD59* | si-*CD59*-1 | GTGTCTCATTACCAAAGCT |
|  | si-*CD59*-2 | GGGACATCCTTATCAGAGA |
|  | si-*CD59*-3 | GGTTACAAGTGTATAACAA |
| *CD63* | si-*CD63*-1 | GCTGCCTCGTGAAGAGTAT |
|  | si-*CD63*-2 | CTTCGATCCTGGACAGGAT |
|  | si-*CD63*-3 | GATGGAGAATTACCCGAAA |
| *LAMP1* | si-*LAMP1*-1 | ACATGAAGGTCCTCTACTT |
|  | si-*LAMP1*-2 | GAATCCAGTTGAATACAAT |
|  | si-*LAMP1*-3 | CCTTCTCAGTGAACTACGA |
| *PODXL* | si-*PODXL*-1 | ACATGACCATCTTATGAAA |
|  | si-*PODXL*-2 | GGACTCATCTAACAAAACA |
|  | si-*PODXL*-3 | AGACCGTGGTCGTCAAAGA |
| *CD44* | si-*CD44*-1 | GCAGTCAACAGTCGAAGAA |
|  | si-*CD44*-2 | CTCTGAGCATCGGATTTGA |
|  | si-*CD44*-3 | GACCTCTGCAAGGCTTTCA |
| *ITGB1* | si-*ITGB1*-1 | CCAGCCCATTTAGCTACAA |
|  | si-*ITGB1*-2 | CCCTAAGTCAGCAGTAGGA |
|  | si-*ITGB1*-3 | CAACGGACAGATCTGCAAT |
| *ANPEP* | si-*ANPEP*-1 | GGAAGAAGCTTTTTAACGA |
|  | si-*ANPEP*-2 | GGAGGAAGATTCAGACTCA |
|  | si-*ANPEP*-3 | GGGCACAGATCATTAATGA |
| *BSG* | si-*BSG*-1 | TGGGCCTGGTACAAGATCA |
|  | si-*BSG*-2 | CCATCATCTTCATCTACGA |
|  | si-*BSG*-3 | GCAGCACCAGAATGACAAA |
| *NPTN* | si-*NPTN*-1 | GACTGGATATGGCGCAAGA |
|  | si-*NPTN*-2 | CAAGGATTGTCACCAGTGA |
|  | si-*NPTN*-3 | CCAACAATCACAAAGATAA |
| *TCF7L2* | si-*TCF7L2*-1 | CGAAAGUUUCCGAGACAAAUC |
|  | si-*TCF7L2*-2 | CAGAGAAGAGCAAGCGAAAUA |
|  | si-*TCF7L2*-3 | CCGAAAGUUUCCGAGACAAAU |
| *LEF1* | si-*LEF1*-1 | GGUCUGCAAGAGACAAUUAUG |
|  | si-*LEF1*-2 | CAUCAGAUGUCAACUCCAAAC |
|  | si-*LEF1*-3 | GCGAUUUAGCUGACAUCAAGU |

**Table S16** Primer sequences designed for qRT-PCR.

| **Gene** | **Forward primer 5′–3′** | **Reverse primer 5′–3′** |
| --- | --- | --- |
| *GAPDH* | AACGTGTCAGTGGTGGACCTG | AGTGGGTGTCGCTGTTGAAGT |
| *FASLG* | TGCCTTGGTAGGATTGGGC | GCTGGTAGACTCTCGGAGTTC |

**Table S17** The details of antibodies used in the current study.

| **Antibody** | **Host** | **Company (Cat No.)** |
| --- | --- | --- |
| Anti-mouse-GAPDH | Mouse | Proteintech, USA (60004-1-Ig) |
| Anti-Vimentin | Mouse | Abcam, UK (ab8069) |
| Anti-Vimentin | Rabbit | CST, USA (#5741) |
| Anti-COL1A2 | Mouse | Proteintech, USA (66761-1-Ig) |
| Anti-α-SMA | Rabbit | Proteintech, USA (14395-1-AP) |
| Anti-CLCNKA | Rabbit | Proteintech, USA (14402-1-AP) |
| Anti-E-cadherin | Mouse | Abcam, UK (ab40772) |
| Anti-CD34 | Rabbit | Abcam, UK (ab81289) |
| Anti-PXN | Rabbit | Proteintech, USA (10029-1-Ig) |
| Anti-ITGB1 | Rabbit | Abcam, UK (ab52971) |
| Anti-FASLG | Rabbit | Abcam, UK (ab134401) |
| Anti-FASLG | Mouse | Abcam, UK (ab303666) |
| Anti-FAS | Rabbit | Abcam, UK (ab133619) |
| Anti-UMOD | Rabbit | Proteintech, USA (11911-1-AP) |
| Anti-NPT2A | Rabbit | Proteintech, USA (26783-1-AP) |
| Anti-Keratin-13 | Rabbit | Proteintech, USA (10164-2-AP) |
| Anti-THY1 | Mouse | Proteintech, USA (66766-1-Ig) |
| Anti-GSK3α/β | Rabbit | CST, USA (#5676) |
| Anti-p-GSK3α/β (Ser21/9) | Rabbit | CST, USA (#8566) |
| Anti-HSP27 | Rabbit | CST, USA (#2442) |
| Anti-p-HSP27(Ser82) | Rabbit | CST, USA (#2401) |
| Anti-β-catenin | Rabbit | Proteintech, USA (51067-2-AP) |
| Anti-p-β-catenin | Rabbit | Proteintech, USA (28853-1-AP) |
| Anti-OPN | Rabbit | Abcam, UK (ab214050) |
| Anti-NDP | Rabbit | Abcam, UK (ab185715) |
| Anti-SFRP1 | Rabbit | Abcam, UK (ab126613) |
| Anti-FZD4 | Mouse | SCBT, USA (sc-293454) |
| Anti-Cre Recombinase | Rabbit | CST, USA (#15036) |
| HRP-conjugated anti-Rabbit IgG | Goat | Proteintech, USA (SA00001-2) |
| HRP-conjugated anti-Mouse IgG | Goat | Proteintech, USA (SA00001-1) |
| Anti-Mouse IgG H&L (Alexa Fluor®488) | Goat | Abcam, UK (ab150113) |
| Anti-Rabbit IgG H&L (Alexa Fluor®488) | Goat | Abcam, UK (ab150077) |
| Anti-Mouse IgG H&L (Alexa Fluor®647) | Goat | Abcam, UK (ab150115) |
| Anti-Rabbit IgG H&L (Alexa Fluor®647) | Goat | Abcam, UK (ab150083) |

**Table S18** List of 321 candidate compounds identified from the HY-L001P Library by virtual screening against THY1 (Excel file).

**Table S19** List of 1,449 candidate compounds identified from the HY-L901P Library by virtual screening against THY1 (Excel file).

**References**

[1] M. Liu, Z. Liu, F. Huang, H. Chen, Z. Yang, Z. Zhu, *Urolithiasis* **2024**, *52*, 122.

[2] Z. Zhu, F. Huang, Y. Jiang, S. Ruan, M. Liu, Y. Zhang, Y. Li, J. Chen, Y. Cui, Z. Chen, H. Chen, F. Zeng, *Mol Med* **2022**, *28*, 162.

[3] A. Subramanian, P. Tamayo, V. K. Mootha, S. Mukherjee, B. L. Ebert, M. A. Gillette, A. Paulovich, S. L. Pomeroy, T. R. Golub, E. S. Lander, J. P. Mesirov, *Proc Natl Acad Sci U S A* **2005**, *102*, 15545.

[4] A. Liberzon, C. Birger, H. Thorvaldsdóttir, M. Ghandi, J. P. Mesirov, P. Tamayo, *Cell Syst* **2015**, *1*, 417.

[5] Z. Zhu, F. Huang, W. Xia, H. Zeng, M. Gao, Y. Li, F. Zeng, C. He, J. Chen, Z. Chen, Y. Li, Y. Cui, H. Chen, *Front Cell Dev Biol* **2020**, *8*, 596363.

[6] J. R. Wiśniewski, A. Zougman, N. Nagaraj, M. Mann, *Nat Methods* **2009**, *6*, 359.

[7] T. Aoyagi, M. Hatsu, F. Kojima, C. Hayashi, M. Hamada, T. Takeuchi, *J. Antibiot.* **1992**, *45*, 1079.

[8] J. Yang, Y. Zhi, S. Wen, X. Pan, H. Wang, X. He, Y. Lu, Y. Zhu, Y. Chen, G. Shi, *J Nutr Biochem* **2023**, *111*, 109178.

[9] H. Liu, X. Li, C. Zhang, X. Hao, Y. Cao, Y. Wang, H. Zhuang, N. Yu, T. Huang, C. Liu, H. Cao, Z. Lu, J. Song, L. Liu, H. Wang, Z. Li, W. Tang, *Adv Sci (Weinh)* **2024**, *11*, e2402115.
